# *Mycobacterium tuberculosis* Inhibitors Based on Arylated Quinoline Carboxylic Acid Backbones with Anti-*Mtb* Gyrase Activity

**DOI:** 10.3390/ijms241411632

**Published:** 2023-07-19

**Authors:** Mark Tristan J. Quimque, Adrian D. Go, Justin Allen K. Lim, Warren S. Vidar, Allan Patrick G. Macabeo

**Affiliations:** 1 The Graduate School, University of Santo Tomas, España Blvd., Manila 1015, Philippines; marktristan.quimque.gs@ust.edu.ph; 2 Laboratory for Organic Reactivity, Discovery and Synthesis (Rm. 410), Research Center for the Natural and Applied Sciences, University of Santo Tomas, Espana Blvd., Manila 1015, Philippines; adrian_d_go@dlsu.edu.ph (A.D.G.); justin.allen.lim@gmail.com (J.A.K.L.); wsvidar@uncg.edu (W.S.V.); 3 Chemistry Department, College of Science and Mathematics, Mindanao State University-Iligan Institute of Technology, Tibanga, Iligan City 9200, Philippines

**Keywords:** *Mycobacterium tuberculosis* H_37_Rv, antitubercular, quinoline carboxylic acid, *Mtb* gyrase

## Abstract

New antitubercular agents with either a novel mode of action or novel mode of inhibition are urgently needed to overcome the threat of drug-resistant tuberculosis (TB). The present study profiles new arylated quinoline carboxylic acids (QCAs) having activity against replicating and non-replicating *Mycobacterium tuberculosis* (*Mtb*), the causative agent of TB. Thus, the synthesis, characterization, and in vitro screening (MABA and LORA) of 48 QCAs modified with alkyl, aryl, alkoxy, halogens, and nitro groups in the quinoline ring led to the discovery of two QCA derivatives, **7i** and **7m,** adorned with C-2 2-(naphthalen-2-yl)/C-6 1-butyl and C-2 22-(phenanthren-3-yl)/C-6 isopropyl, respectively, as the best *Mtb* inhibitors. DNA gyrase inhibition was shown to be exhibited by both, with QCA **7m** illustrating better activity up to a 1 μM test concentration. Finally, a docking model for both compounds with *Mtb* DNA gyrase was developed, and it showed a good correlation with in vitro results.

## 1. Introduction

Tuberculosis (TB) poses serious health risks to humans. Annually, this deadly infectious disease slowly affects millions of people worldwide causing illness and devastating fatalities. The global burden for TB incidences is accounted for by Southeast Asia at 41%, with Indonesia (14%) and the Philippines (12%) having the biggest increases in the number of diagnosed TB infections between 2019 and 2020 [1]. These are among the countries which had significant impacts on the worrisome global prevalence of the disease and were estimated to reach 10 million in 2012, with 1.3 million people dying from TB-related causes in 2022 [2]. The alarmingly high death rate is mostly due to poor diagnostic tools, the lack of universal healthcare, and the rising cases of strains resistant to most if the currently available anti-TB antibiotics. A strain of TB that is resistant to the two first-line medications (such as rifampicin and isoniazid) is known as a multidrug-resistant strain (MDR-TB). The extremely drug-resistant TB strain (XDR-TB), on the other hand, is a variety of DR-TB that is resistant to first-line medications, as well as fluoroquinolones and one of the second-line injectable anti-TB medications (such as amikacin, capreomycin, and kanamycin) [3].

The constant need to search for novel anti-TB antibiotics grows as more *Mycobacterium tuberculosis (Mtb)* strains become resistant. The creation of new synthetic or semi-synthetic medications, the discovery of structural variations (which is typically performed for chemical structure optimization), and the isolation of natural products are among the key avenues worth investigating [4,5,6,7]. The structural complexity of natural products allows for the investigation of a wide range of alternative scaffolds [8], resulting in the discovery of some of the most effective anti-TB antibiotics to date [9].

DNA gyrase, a type II DNA topoisomerase, has been a successful target for antitubercular drug therapy. DNA topoisomerases are classified as type I and type II, which generate transitory single- and double-stranded breaks in DNA, respectively. Each class possesses at least one enzyme, which is categorized as type IA, IB, IIA, or IIB depending on their functions. One instance of each type I and type II (DNA gyrase) topoisomerase is encoded by the *Mtb* genome. This is a crucial component of the drug development process because it offers a target amenable to inhibition studies. The enzyme DNA gyrase has the ability to cause negative supercoiling in DNA, which lessens strain as DNA is unwound. It is a heterotetrameric (A2B2) structure which contains two GyrA and GyrB subunits. While the GyrB subunit assists in the ATP hydrolysis, which supplies energy for DNA-replication processes, the GyrA subunit includes tyrosine at the active site essential for cleavage and relegation of DNA [10]. Synthetic quinoline derivatives have been used successfully as anti-tubercular drugs by inhibiting DNA gyrase [11]. For example, the fluoroquinolones are used to treat TB and target the GyrA subunit. However, *Mtb* resistance to these medications has reduced their effectiveness, indicating that the GyrB domain may prove to be a promising target for discovering novel anti-TB medicines [12].

Quinoline is a ubiquitous substructural core in a number of anti-tubercular natural products [13] and pharmaceuticals. This important class of *N*-bearing heterocyclic compound displays a broad spectrum of biological properties, such as anti-malarial, anticancer, antifungal, antibacterial, anti-obesity, anti-inflammatory, and anti-tuberculosis activities. While a range of pharmacological activities have been reported for this structural class [14], few studies have been devoted for the discovery of new anti-TB drugs in comparison to other classes. Moreover, 2-substituted quinoline alkaloids from the Philippine endemic Rutaceae plant *Lunasia amara* Blanco have been reported to display significant activity against *Mtb* H_37_Rv. Hence, in this context, the present study draws its inspiration from antitubercular natural products and synthetic quinoline alkaloids as part of our ongoing conquest of finding novel derivatives to mitigate TB. In this paper, we disclose the design, synthesis, and screening of new 2-arylquinoline 4-carboxylic acid (QCA) analogs as antitubercular agents with targeting properties on *Mtb* DNA gyrase.

## 2. Results and Discussion

### 2.1. Chemistry

#### 2.1.1. Synthesis Intermediate of Substituted Isatins

**Scheme 1:**
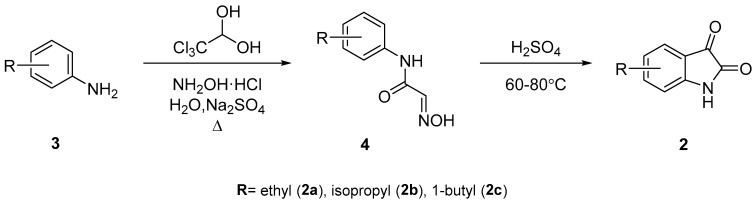
Synthesis of substituted isatins **2a–2c** via Sandmeyer reaction.

As a requisite for the synthesis of quinoline-4-carboxylic acids (**1**), an additional three new substituted isatins (5-ethylindoline-2,3-dione (**2a**), 5-isopropylindoline-2,3-dione (**2b**), and 5-butylindoline-2,3-dione (**2c**)) were prepared using Sandmeyer’s reaction. In this reaction, substituted aniline (**3**) reacts with chloral hydrate and hydroxylamine hydrochloride in aqueous sodium sulfate solution. The obtained isonitrosoacetenilide intermediate is further reacted with sulfuric acid to form isatin [15]. The synthesized substituted isatins were used in the next step for the synthesis of QCA (Scheme 1).

#### 2.1.2. Synthesis of Quinoline Carboxylic Acids 1

**Scheme 2:**
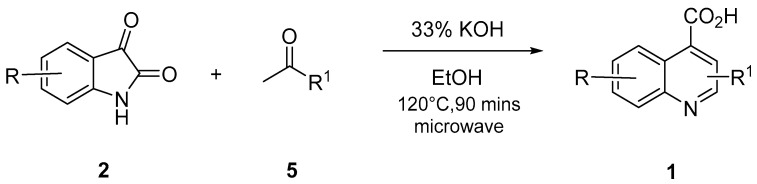
Synthesis of quinoline carboxylic acid derivatives (**1**) via Pfitzinger reaction.

The procedure outlining the synthesis of compounds **1a–1p**, **6a–6r,** and **7a–7n** is shown in Scheme 2. The reaction highlights a microwave-assisted Pfitzinger reaction using substituted isatins (**2**) and substituted ketones (**5**) as precursors to afford quinoline-4-carboxylic acid (QCA, **1**). The Pfitzinger reaction is a two-component reaction which mechanism shows the alkaline hydrolysis-driven ring-opening of the pyrrolidinone ring of isatin as an initial step to produce 2-(2-aminophenyl)-2-oxoacetic acid in situ. The keto-acid aniline intermediate reacts further with the enolate of ketone **5** to give an imine which is eventually converted to an enamine. The final cyclization occurs via hydrolysis of the enamine to give the desired substituted QCA **1** [16,17]. All synthesized quinoline carboxylic acid derivatives **1** were characterized using NMR and HR-ESIMS for structure characterization and/or verification.

### 2.2. Antitubercular Activity of QCAs 1

The quinoline carboxylic acid (QCA, **1**) derivatives were evaluated against *Mtb* H_37_Rv, using the in vitro colorimetric susceptibility Microplate Alamar Blue Assay (MABA). In this assay, resazurin, non-fluorescent blue dye, is reduced to the pinkish fluorescent dye resorufin by active cells. The potency of the test compounds is taken depending on the reduction of resazurin through quantitative measurements of each microtiter well [18]. Compounds exhibiting TB activity were subsequently subjected to an additional inhibitory assay to determine their effectiveness against non-replicating persistent (NRP) TB, using the low oxygen recovery assay (LORA). LORA employs a recombinant *Mtb* expressing luciferase as the reporter gene since its luminescent intensity is indicative of a cell’s overall metabolic activity in hypoxic conditions. The NRP of *Mtb* is a contributing factor to its virulence, as it generally develops antibiotic resistance to existing medications [19,20]. The viability of the cells is dependent on the luminosity of the luciferase after the normoxic “recovery” period of the test compounds [21]. The antitubercular activity of all synthetic QCAs is expressed as the minimum inhibitory concentration (MIC_90_), as µg/mL, which is defined as the lowest drug concentration needed for the visible growth inhibition of 90% of bacteria in the colony in relation to those in the drug-free controls [22]. Thus, an initial library of sixteen synthetic QCAs **1a–1p** functionalized with alkyl, aryl, and halogens at C-2, C-3, C-6, and C-8 were prepared. In general, most QCA derivatives (Table 1) exhibited poor anti-TB activity (MIC_90_ > 64 μg/mL). Compound **1h**, however, showed weak inhibition, with an MIC_90_ of 62.57 μg/mL, which led us to explore more active derivatives by further functionalizing a 2-([1,1’-biphenyl]-4-yl) QCA scaffold ushering to a subset of derivatives adorned with halogens and alkyl groups.

Eighteen additional derivatives based on 2-([1,1’-biphenyl]-4-yl) QCA **6a–6i** functionalized with halogens at C-6, C-7, or C-8 positions of the quinoline ring are shown in Table 2. Thus, the introduction of halogens at C-8 (**6b, 6d,** and **6f**) led to the loss of anti-TB activity, whereas the variation of halogens at C-6 (**6a, 6c, 6e**) gratifyingly showed a 2-fold increase in *M. tb.* H_37_Rv inhibition, with 6-chloro derivative **6a** as the most inhibitory compound. Among the halogen substitutions at C-6, iodo derivative **6g** exhibited a decrease in anti-TB activity (MIC_90_ > 64.0 μg/mL). Overall, the effective inhibition of the compounds against *M. tb.* H_37_Rv is influenced by the correct positioning of the halogen atoms in the benzene ring, i.e., at C-6. We also attempted to prepare additional derivatives modified with nitro at C-6 (**6h**) and methoxy at C-7 (**6i**); however, both showed poor potency toward *M. tb.* H_37_Rv. We also explored the structural activity by varying the C-2 aryl groups based on the most active compound **6a**.

However, this led to a significant drop in inhibition when the biphenyl group was replaced with 2-(naphthalen-2-yl), 2-(anthracen-2-yl) and 2-(phenanthren-3-yl) handles even by changing the position and type of halogen. Interestingly, the 2-(naphthalen-2-yl)- and 2-(phenanthren-3-yl)-modified 6-bromo derivatives **6q** and **6r** showed moderate anti-TB activity.

Subsequently, compounds **6a, 6c, 6e, 6q,** and **6r**, which showed anti-TB activity in MABA, were further tested against non-replicating *M. tb.*, using the LORA assay. The LORA assay is a high-capacity screening method that is used to assess the efficacy against the non-replicating persistent *Mtb* phenotype in low-oxygen conditions. Interestingly, the tested compounds, compounds **6a**, **6c**, **6q**, and **6r**, showed retention of inhibitory activity against non-replicating *M. tb*. However, the LORA evaluation of 6-bromo QCA modified with 2-([1,1’-biphenyl]-4-yl) **6e** resulted in a loss of activity.

A second generation of 14 compounds, i.e., **7a–7n**, founded on the structure of on 2-([1,1’-biphenyl]-4-yl) QCA with alkyl modifications at C-6 of the quinoline ring is shown in Table 3. We identified a promising *Mtb* activity on compound **7a**, where a methyl group at C-6 is incorporated. Based on this finding, we attempted to investigate the effect of lengthening the alkyl chain of the C-6 position and also varying the aryl groups at the C-2 position to the anti-TB activity. The 1-biphenyl series (**7a–7d**) exhibited good MABA activity, except for the C-6 ethyl-substituted QCA derivative, **7b**. The 2-naphthalene series (**7e–7i**) indicated poor activity irrespective of the alkyl substituent present, except however, with the 1-butyllated QCA **7i** derivative, which showed high inhibitory anti-TB activity against *M. tb.* H_37_Rv (MIC_90_ > 16 μg/mL). The 2-phenanthrene modified series (**7j, 7l–7n)**) showed similar potency featuring C-6 methyl and 1-butyl substituents. The ethyl substituted C-6 (**7l**) displayed the weakest activity (MIC_90_ = 51.3 μg/mL) among the 2-phenanthrenylated series. Finally, the isopropyl substituted C-6 also showed the highest activity, with MIC_90_ of >16 μg/mL. Based on the results, the pattern shows unfavorable activity when the C-6 position is ethylated. The overall trend indicates that, with the substitution of C-6 positions with 1-butyl, the effectiveness of the compound against *Mtb* is enhanced. Retention of LORA activity was observed on compounds **7a** and **7c**. The LORA activity of compound **7d** improved slightly from MIC_90_ > 32 to MIC_90_ = 19.84, whereas compounds **7j**, **7k**, and **7n** resulted in having a loss of inhibitory potency. The activity of hit compounds **7i** and **7m** was conserved in LORA.

### 2.3. Mtb DNA Gyrase Inhibitory Activity

DNA gyrase is a type II DNA topoisomerase found in bacteria, including mycobacteria. This enzyme is crucial for the replication and transcription functions of bacterial DNA, making it an interesting target for discovering antitubercular agents. Quinolones, which are an oxidized derivative of quinolines, are known to target type II topoisomerase (DNA gyrase and topoisomerase IV). Since *Mtb* does not possess topoisomerase IV, DNA gyrase is the key quinolone target [23]. This class of antitubercular compounds acts on DNA gyrase by inducing breaks on the DNA strand which subsequently result in cell lysis because of accumulated oxidative stress in the strand [24,25]. With this premise, the bioactive inhibitory quinoline carboxylic acid derivatives were investigated for their inhibitory potentials against DNA gyrase in vitro, using a DNA gyrase supercoiling assay. Based on the in vitro activity data in Table 3, compounds **7i** and **7m** were evaluated for their effectiveness in inhibiting DNA gyrase (Figure S55).

**Figure 1:**
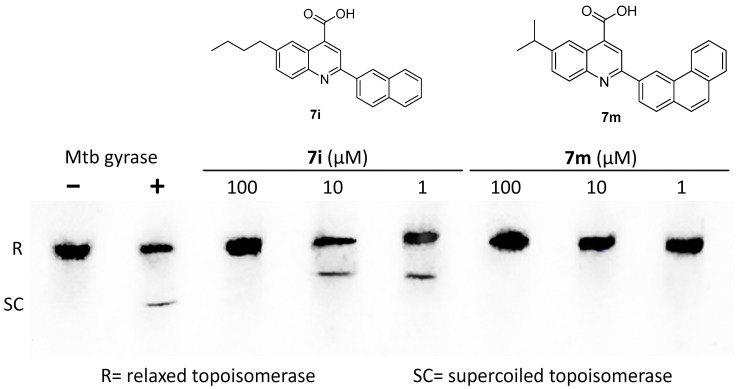
Agarose gel electrophoresis result showing inhibition of QCA **7i** and **7m** against *Mtb* DNA gyrase; (−) negative control without gyrase, and (+) positive control with gyrase.

The assay involves a gyrase to create supercoiled DNA from a circular DNA plasmid called pBR322, which is initially in a relaxed state. The supercoiled and relaxed versions of the plasmid can be distinguished by running an agarose gel using electrophoresis. Herein, three concentrations of compounds **7i** and **7m** were tested on their inhibitory activity against DNA gyrase (Figure 1). The target enzyme was only inhibited by QCA **7i** at a 100 µM concentration, while QCA **7m** inhibited *Mtb* DNA gyrase at 1.0 µM, as evidenced by an absence of supercoiled bands in their respective lanes. These suggest that the active compounds can inhibit the mycobacterial enzyme, which can be inferred to act as a potential antimycobacterial agent.

### 2.4. Molecular Docking of 7i and 7m onto Mtb DNA Gyrase

**Figure 2:**
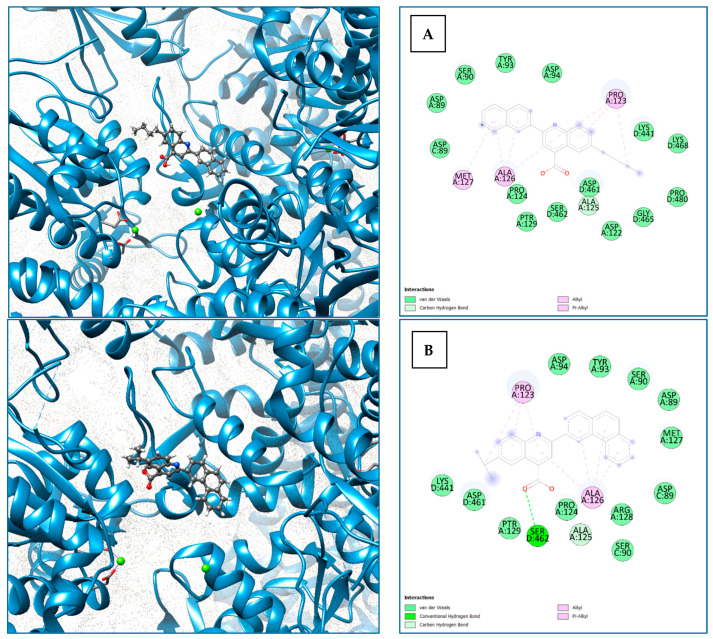
The 3D and 2D docking poses of (**A**) QCA **7i** and (**B**) **7m** at the binding site of *Mtb* Gyrase (PDB ID: 5BTN).

Both quinoline carboxylic acids **7i** and **7m** were subjected to in silico molecular docking analysis to assess the potential binding interactions of the in vitro active compounds with the fluoroquinolone-binding site of *Mtb* DNA gyrase (Table 4). After the docking between the two compounds, **7m** exhibited a stronger binding to the active site of the enzyme, with a binding energy of −8.0 kcal/mol (Figure 2). The quinoline core of the compound and its isopropyl substituent were stabilized by pi-alkyl interaction with Pro123. The 2-phenanthrene side chain had all three of its aromatic rings bound to Ala126 via pi-alkyl intermolecular forces. Moreover, the carboxylic acid moiety interacted through conventional hydrogen bonding (bond distance of 3.68 Å) with Ser462. Quinoline **7i**, on the other hand, was nestled to the enzyme’s active site through pi-alkyl/alkyl interplay with Pro123, Ala126, and Met127. A carbon–hydrogen bonding can also be observed between the 4-carboxylic acid group and Ala125.

Corroborating the results of both the in vitro and in silico analysis performed on the two hit quinoline compounds, we found that QCA **7m** had a better binding energy score than compound **7i** that was brought about by various pi-alkyl interactions with the amino acids in the enzyme, leading to the inhibition of *Mtb* DNA Gyrase even at a low concentration.

## 3. Materials and Methods

### 3.1. Materials and Instrumentations

All reagents and other materials were obtained from Sigma Aldrich (St. Louis, MO, USA) and Alfa Aesar (Heysham, Lancashire, UK) and used without additional purification, unless otherwise indicated. A JEOL 600 MHz ECZ600R NMR spectrometer (Kobe, Japan) was used to generate NMR spectra, with CDCl_3_, MeOH-*d_4_*, or DMSO-*d_6_* serving as solvents. A Thermo Fisher Q ExactiveTM Plus mass spectrometer (Thermo Fisher Scientific, Waltham, MA, USA) equipped with an electrospray ionization (ESI) source coupled with a Waters Acquity ultra-performance liquid chromatography (Waters Corporation, Milford, MA, USA) was used for mass spectroscopy. A 3 µL volume of each sample was injected and eluted through a reversed-phase column (BEH C18, 1.7 µm, 2.1 × 50 mm, Waters Corporation), using a binary solvent system consisting of water with 0.1% formic acid (solvent A) and acetonitrile with 0.1% formic acid (solvent B). Microwave-assisted reactions were performed using Biotage^®^ Initiator+ (Uppsala, Sweden). TLC spots were visualized under UV light (254 nm and 365 nm), followed by spraying with a vanillin–sulfuric acid or Dragendorff’s reagent. *Mtb* DNA gyrase inhibitions were tested with *Mtb* Gyrase (HIS) Supercoiling Kit 1 (Inspiralis, Norwich, UK) containing *Mtb* Gyrase Assay Buffer: 50 mM HEPES. KOH (pH 7.9), 6 mM magnesium acetate, 4 mM DTT, 1 mM ATP, 100 mM potassium glutamate, 2 mM spermidine, 0.05 mg/mL albumin, Dilution Buffer: 50 mM Tris.HCl (pH 7.9), 5 mM DTT, 30 % (*w*/*v*) glycerol, *Mtb* gyrase, and relaxed pBR322 plasmid. The electrophoresis system used was Mupid^®^-exu (Tokyo, Japan).

### 3.2. Synthesis

The synthetic approach for quinoline carboxylic acid derivatives in this study is outlined above, in Scheme 2. The starting isatin compounds, 5-ethylindoline-2,3-dione, 5-isopropylindoline-2,3-dione, and 5-butylindoline-2,3-dione, were synthesized using the method described in Section 3.2.1, and the rest of the isatins were obtained from commercial sources and used without additional purification.

#### 3.2.1. General Approach for the Synthesis of Substituted Isatins 2

A mixture of substituted aniline (0.05 mmol), hydroxylammonium chloride (0.15 mmol), Na_2_SO_4_ (0.35 mmol), hydrochloric acid (10 mmol), and chloral hydrate (0.06 mmol) in 50 mL water was stirred at 95 °C, over a 180 min period. The completion of this reaction was determined via TLC (1:1 petroleum ether/ethyl acetate). Next, the mixture was cooled at room temperature, filtered, and dried. The obtained precipitates were dissolved in concentrated sulfuric acid (20 mL), with vigorous stirring, at 50 °C. Then, the mixture was heated to 80 °C for 30 min. The reaction mixture was allowed to cool until room temperature, after which it was poured into ice. The solids were filtered and purified by dissolving them in 5% NaOH (100 mL), followed by the addition of 4M HCl (50 mL). The formed precipitates were filtered and air-dried to obtain isatin **2**, which was subsequently used for the method described in Section 3.2.2, without additional purification.

#### 3.2.2. General Approach for the Synthesis of QCA 1, 6, and 7

A mixture of substituted isatin **2** (1 mmol), ketone **5** (1.0 mmol), and 33% potassium hydroxide (0.1 mL per reactant mmol of isatin) in 2–10 mL ethanol was placed in a closed Teflon vessel and irradiated on a microwave instrument for 90 min at 120 °C. The reaction mixture was dried in vacuo and washed thrice with diethyl ether. The obtained aqueous layer was cooled at 0–10 °C, and glacial acetic acid was slowly added until a pH of 4 was attained. The precipitates that formed were filtered and washed with cold ethanol. The obtained precipitates were purified by recrystallization, using ethanol and water (V_EtOH_:V_water_ = 1:1), or by silica gel column chromatography, using dichloromethane (DCM) and methanol (MeOH) (V_DCM_:V_MeOH_ = 15:1), to yield compounds **1a–1p, 6a–6r,** and **7a–7n** (Supplementary Figures S1–S54).

***2-pentylquinoline-4-carboxylic acid* (1a):** Neon yellow powder, yield 51.0%, Rf = 0.27 (5:1 EtOAc-MeOH). ^1^H NMR (600 MHz, MeOH-*d_4_*) δ 8.44 (s, 1H), 7.96 (d, *J* = 7.9 Hz, 1H), 7.69 (t, 1H), 7.65–7.48 (m, 2H) 2.94 (t, 2H), 1.90–1.73 (m, 2H), 1.51–1.30 (m, 4H), 0.89 (t, 3H). ^13^C NMR (151 MHz, MeOH-*d_4_*) δ 173.9, 162.9, 147.5, 146.6, 129.5, 127.05, 126.4, 125.9, 123.6, 119.2, 38.1, 31.4, 29.5, 22.9, 13.0.

***2-phenethylquinoline-4-carboxylic acid* (1b):** Beige crystals, yield 92.0%, Rf = 0.25 (6:1 EtOAc-MeOH). ^1^H NMR (600 MHz, DMSO-*d_6_*) δ 7.57 (dd, *J* = 8.5, 1.0 Hz, 1H), 7.03–6.98 (m, 1H), 6.81 (s 1H), 6.74 (ddd, *J* = 8.3, 6.8, 1.4 Hz, 1H), 6.59 (ddd, *J* = 8.4, 6.9, 1.3 Hz, 1H), 6.26–6.18 (m, 3H), 6.15–6.08 (m, 1H), 2.26–2.20 (m, 2H), 2.06 (dd, *J* = 9.4, 6.7 Hz, 2H. ^13^C NMR (151 MHz, DMSO-*d_6_*) δ 168.2, 162.0, 148.6, 141.8, 136.8, 130.2, 129.6, 129.0, 128.8, 127.6, 126.4, 125.9, 123.5, 122.9, 40.2, 35.1.

***2-benzyl-3-phenylquinoline-4-carboxylic acid* (1c):** Beige powder, yield 50.0%, Rf = 0.15 (6:1 EtOAc-MeOH). ^1^H NMR (600 MHz, DMSO-*d_6_*) δ 8.04 (d, *J* = 8.4 Hz,1H), 7.84–7.72 (m,2H), 7.62 (t, *J* = 7.4 Hz,1H), 7.47–7.31 (m,3H), 7.14 (d, *J* = 6.2 Hz,2H), 7.07 (dq, *J* = 14.0, 6.9 Hz,3H), 6.78 (d, *J* = 7.1 Hz,2H), 4.09 (s,2H). ^13^C NMR (151 MHz, DMSO-*d_6_*) δ 168.5, 159.7, 146.9, 139.3, 137.0, 130.5, 130.2, 130.1, 129.4, 129.0, 128.7, 128.6, 128.5, 128.4, 127.8, 126.5, 125.6, 122.3, 42.8.

***2-benzhydrylquinoline-4-carboxylic acid* (1d):** Beige crystals; yield 63.0%, Rf = 0.36 (6:1 EtOAc-MeOH). ^1^H NMR (600 MHz, DMSO-*d_6_*) δ 8.62 (dd, *J* = 8.6, 1.1 Hz, 1H), 8.01–7.98 (m, 1H), 7.79 (s, 1H), 7.76 (ddd, *J* = 8.4, 6.9, 1.4 Hz, 1H), 7.64 (ddd, *J* = 8.4, 6.9, 1.3 Hz, 1H), 7.30–7.27 (m, 4H), 7.26–7.23 (m, 4H), 7.22–7.18 (m, 2H), 5.97 (s, 1H). ^13^C NMR (151 MHz, DMSO- *d_6_*) δ 168.0, 163.2, 148.6, 142.8, 137.2, 130.5, 130.0, 129.7, 129.0, 128.2, 127.1, 126.0, 123.6, 123.5, 56.6. HRMS (ESI): calculated [M + H^+^]^+^ 340.1338, found 340.1334.

**2,6-dimethylquinoline-4-carboxylic acid (1e):** White flakes, yield 24.9%, Rf = 0.14 (6:1 EtOAc-MeOH). ^1^H NMR (600 MHz, CDCl_3_) δ 8.15 (s, 1H), 7.62 (d, *J* = 8.6 Hz, 1H), 7.48 (d, *J* = 1.6 Hz, 1H), 7.36 (d, *J* = 8.6 Hz, 1H), 2.50 (s, 3H), 2.26 (s, 3H).^13^C NMR (151 MHz, CDCl_3_) δ 168.8, 157.0, 144.2, 141.9, 138.0, 133.3, 129.4, 125.2, 123.8, 122.3, 22.9, 21.4.

**6-chloro-2-methylquinoline-4-carboxylic acid (1f):** Pale yellow powder, yield 76.0%, Rf = 0.14 (6:1 EtOAc-MeOH). ^1^H NMR (600 MHz, DMSO-*d_6_*) δ 8.69 (s, 1H), 7.93 (s, 1H), 7.84 (s,1H), 7.71 (s, 1H), 2.64 (s, 3H). ^13^C NMR (151 MHz, DMSO-*d_6_*) δ 167.7, 159.9, 147.1, 135.8, 132.0, 131.4, 130.5, 124.9, 124.6, 124.2, 25.2.

***2-phenylquinoline-4-carboxylic acid* (1g):** Yellow powder, yield 88.0%, Rf = 0.45 (5:1 EtOAc-MeOH). ^1^H NMR (600 MHz, MeOH-*d_4_*) δ 8.57 (d, *J* = 8.4 Hz, 1H), 8.18 (s, 1H), 8.10 (m, 3H), 7.78–7.73 (m, 1H), 7.61–7.41 (m, 4H). ^13^C NMR (151 MHz, MeOH-*d_4_*) δ 171.6, 157.4, 148.5, 144.0, 139.1, 129.7, 129.4, 128.6, 127.4, 126.7, 126.1, 124.1, 118.1.

**2-([1,1’-biphenyl]-4-yl)quinoline-4-carboxylic acid (1h):** Beige powder, yield 79.7%, Rf = 0.21 (6:1 EtOAc-MeOH). ^1^H NMR (600 MHz, DMSO-*d_6_*) δ 7.60 (dd, *J* = 8.5, 0.9 Hz, 1H), 7.46 (s, 1H), 7.36–7.32 (m, 2H), 7.12 (d, *J* = 8.1 Hz, 1H), 6.83–6.78 (m, 3H), 6.70 (dt, *J* = 2.8, 1.7 Hz, 2H), 6.64 (ddd, *J* = 8.3, 6.9, 1.3 Hz, 1H), 6.46–6.42 (m, 1H), 6.37–6.33 (m, 2H). ^13^C NMR (151 MHz, DMSO-*d_6_*) δ 168.2, 155.8, 148.9, 141.9, 139.9, 138.2, 137.4, 130.8, 130.2, 129.6, 128.4, 128.3, 127.7, 127.3, 125.9, 124.0, 119.5.

**2-(naphthalen-1-yl)quinoline-4-carboxylic acid (1i):** Beige powder, yield 54.5%, Rf = 0.38 (6:1 EtOAc-MeOH). ^1^H NMR (600 MHz, DMSO-*d_6_*) δ 8.78 (d, *J* = 2.2 Hz, 1H), 8.77 (d, *J* = 2.4 Hz, 1H), 8.59 (s, 1H), 8.41 (dd, *J* = 8.6, 1.8 Hz, 1H), 8.14 (d, *J* = 9.0 Hz, 1H), 8.12–8.08 (m, 1H), 8.05 (d, *J* = 8.6 Hz, 1H), 7.97–7.93 (m, 1H), 7.80 (dd, *J* = 9.0, 2.5 Hz, 1H), 7.61–7.54 (m, 2H). ^13^C NMR (151 MHz, DMSO-*d_6_*) δ 168.2, 156.7, 147.4, 139.9, 135.6, 134.2, 133.5, 132.4, 132.1, 131.0, 129.4, 129.1, 128.1, 127.8, 127.6, 127.2, 125.1, 124.9, 120.5.

**2-(naphthalen-2-yl)quinoline-4-carboxylic acid (1j):** Neon yellow powder, yield 70.0%, Rf = 0.26 (6:1 EtOAc-MeOH). ^1^H NMR (600 MHz, DMSO-*d_6_*) δ 8.91 (d, *J* = 1.2 Hz, 1H), 8.70 (dd, *J* = 8.5, 0.8 Hz, 1H), 8.68 (s, 1H), 8.53 (dd, *J* = 8.6, 1.8 Hz, 1H), 8.24 (d, *J* = 7.9 Hz, 1H), 8.19–8.15 (m, 1H), 8.12 (d, *J* = 8.7 Hz, 1H), 8.03–8.00 (m, 1H), 7.89 (ddd, *J* = 8.3, 6.8, 1.4 Hz, 1H), 7.74 (ddd, *J* = 8.3, 6.8, 1.3 Hz, 1H), 7.64–7.60 (m, 2H). ^13^C NMR (151 MHz, DMSO-*d_6_*) δ 168.3, 156.2, 149.0, 138.3, 135.8, 134.2, 133.6, 130.8, 130.3, 129.4, 129.0, 128.3, 128.1, 127.7, 127.6, 127.1, 126.02, 125.0, 124.0, 124.0, 119.8.

**2-(anthracen-2-yl)quinoline-4-carboxylic acid (1k):** Brown powder, yield 16.0%, Rf = 0.15 (6:1 EtOAc-MeOH). ^1^H NMR (600 MHz, DMSO-*d_6_*) δ 9.03 (s, 1H), 8.76 (s, 1H), 8.68 (s, 1H), 8.65 (d, *J* = 8.4 Hz,1H), 8.59 (s, 1H), 8.48 (d, *J* = 9.0 Hz,1H), 8.20 (dd, *J* = 15.3, 8.6 Hz,2H), 8.13–8.04 (m,2H), 7.84 (t, *J* = 7.5 Hz,1H), 7.68 (t, *J* = 7.6 Hz,1H), 7.56–7.45 (m,2H). ^13^C NMR (151 MHz, DMSO-*d_6_*) δ 168.3, 156.1, 149.0, 138.5, 135.2, 132.5, 132.1, 131.9, 131.6, 130.8, 130.3, 129.3, 128.8, 128.9, 128.3, 128.3, 128.1, 126.7, 126.5, 126.4, 126.1, 124.5, 124.1, 119.8.

**2-(phenanthren-3-yl)quinoline-4-carboxylic acid (1l):** Yellow powder, yield 33.0%, Rf = 0.15 (6:1 EtOAc-MeOH). ^1^H NMR (600 MHz, DMSO-*d_6_*) δ 9.64 (s,1H), 9.10 (d, *J* = 8.3 Hz,1H), 8.73 (s,1H), 8.61–8.55 (m,2H), 8.21 (d, *J* = 8.3 Hz,1H), 8.14 (d, *J* = 8.3 Hz,1H), 8.01 (d, *J* = 8.4 Hz,1H), 7.91 (s,2H), 7.86–7.80 (m,1H), 7.78–7.71 (m,1H), 7.67 (ddd, *J* = 8.1, 4.0, 1.9 Hz,2H). ^13^C NMR (151 MHz, DMSO-*d_6_*) δ 168.6, 156.4, 148.9, 136.9, 133.0, 132.4, 130.7, 130.6, 130.3, 130.3, 129.8, 129.2, 128.6, 128.1, 127.7, 127.0, 126.2, 126.1, 126.1, 124.1, 123.9, 123.9, 122.3, 119.6.

***2-(2,3-dihydrobenzo[b][1,4]dioxin-6-yl)quinoline-4-carboxylic acid* (1m):** Bright yellow powder, yield 18.6%, Rf = 0.19 (6:1 EtOAc-MeOH). ^1^H NMR (600 MHz, DMSO-*d_6_*) δ 8.56 (dd, *J* = 8.6, 1.0 Hz, 1H), 8.33 (s, 1H), 8.07 (d, *J* = 7.9 Hz, 1H), 7.80–7.76 (m, 2H), 7.75 (dd, *J* = 8.5, 2.2 Hz, 1H), 7.62 (ddd, *J* = 8.3, 6.8, 1.3 Hz, 1H), 6.99 (d, *J* = 8.4 Hz, 1H), 4.32–4.25 (m, 4H) ^13^C NMR (151 MHz, DMSO-*d_6_*) δ 168.2, 155.6, 148.8, 145.9, 144.3, 138.2, 131.7, 130.6, 130.1, 127.9, 125.9, 123.7, 121.0, 119.2, 118.0, 116.3, 64.9, 64.6.

**2-(4-methoxyphenyl)quinoline-4-carboxylic acid (1n):** Beige powder, 58.0%; ^1^H NMR (600 MHz,) δ 8.60 (dd, *J* = 15.7, 8.5 Hz, 1H), 8.05 (dd, *J* = 14.7, 8.4 Hz, 1H), 7.77 (dd, *J* = 11.1, 8.8 Hz, 2H), 7.65–7.62 (m, 1H), 7.25 (dd, *J* = 13.8, 8.4 Hz, 2H), 6.90–6.81 (m, 2H), 3.69 (d, *J* = 13.6 Hz, 3H). ^13^C NMR (151 MHz, DMSO-*d_6_*) δ 168.1, 161.9, 158.4, 148.6, 137.4, 131.5, 131.1, 130.9, 130.3, 129.7, 127.7, 125.9, 122.6, 114.6, 55.5.

**2-(4-fluoro-3-nitrophenyl)quinoline-4-carboxylic acid (1o):** Brown solid, yield 49.1%, Rf = 0.11 (6:1 EtOAc-MeOH). ^1^H NMR (600 MHz, DMSO-*d_6_*) δ 8.71 (d, *J* = 2.4 Hz, 1H), 8.59 (dd, *J* = 8.5, 0.9 Hz, 1H), 8.34 (dd, *J* = 8.8, 2.3 Hz, 1H), 8.18 (s, 1H), 8.01 (d, *J* = 8.1 Hz, 1H), 7.70 (ddd, *J* = 8.3, 6.8, 1.4 Hz, 1H), 7.53 (ddd, *J* = 8.2, 6.8, 1.2 Hz, 1H), 7.28 (d, *J* = 8.8 Hz, 1H). ^13^C NMR (151 MHz, DMSO-*d_6_*) δ 169.4, 156.4, 154.4, 148.7, 138.1, 138,1, 133.4, 130.1, 129.6, 128.1, 127.1, 126.8, 124.5, 124.4, 121.3, 117.1. HRMS (ESI): calculated [M + H^+^]^+^ 313.0625, found 313.0627

**2-(2,3-dihydrobenzo[b][1,4]dioxin-6-yl)-8-fluoroquinoline-4-carboxylic acid (1p):** Yellow powder, yield 62.0%, Rf = 0.15 (6:1 EtOAc-MeOH). ^1^H NMR (600 MHz, MeOH-*d_4_*)δ 8.47 (d, *J* = 8.6 Hz), 8.34 (s), 7.70 (d, *J* = 2.1 Hz), 7.64 (dd, *J* = 8.5, 2.1 Hz), 7.41 (td, *J* = 8.1, 5.3 Hz), 7.36–7.31 (m), 6.92 (d, *J* = 8.4 Hz), 3.57 (s). ^13^C NMR (151 MHz, CDCl_3_) δ 168.3, 158.9, 157.24, 156.3, 145.6, 144.0, 136.6, 132.0, 126.8, 121.4, 121.0, 117.7, 116.7, 116.4, 113.9, 113.8, 64.7, 64.3. HRMS (ESI): calculated [M + H^+^]^+^ 326.0829, found 326.0843

**2-([1,1’-biphenyl]-4-yl)-6-chloroquinoline-4-carboxylic acid (6a):** Neon yellow powder; 28.0% yield, Rf = 0.46 (6:1 EtOAc-MeOH). ^1^H NMR (600 MHz, DMSO-*d_6_*) δ 8.73 (t, *J* = 2.2 Hz, 1H), 8.55–8.53 (m, 1H), 8.36–8.31 (m, 2H), 8.13 (ddd, *J* = 8.9, 4.2, 1.5 Hz, 1H), 7.82 (dt, *J* = 5.3, 4.2 Hz, 3H), 7.75–7.70 (m, 2H), 7.50–7.44 (m, 2H), 7.38 (dd, *J* = 11.0, 3.7 Hz, 1H). ^13^C NMR (151 MHz, DMSO-*d_6_*) δ 167.6, 156.3, 147.5, 142.2, 139.8, 136.9, 136.6, 132.8, 132.4, 131.2, 129.6, 128.5, 128.3, 127.7, 127.2, 124.9, 124.8, 121.0.

**2-([1,1’-biphenyl]-4-yl)-8-chloroquinoline-4-carboxylic acid (6b):** Neon yellow powder, yield 15.5%, R_f_ = 0.24 (6:1 EtOAc-MeOH). ^1^H NMR (600 MHz, DMSO-*d_6_*) δ 8.52 (dd, *J* = 8.5, 0.9 Hz, 1H), 8.48 (s, 1H), 8.39 (d, *J* = 8.4 Hz, 1H), 7.96 (dd, *J* = 7.5, 1.0 Hz, 1H), 7.82 (d, *J* = 8.4 Hz, 2H), 7.71 (d, *J* = 7.4 Hz, 2H), 7.57 (dd, *J* = 8.4, 7.6 Hz, 1H), 7.45 (t, *J* = 7.7 Hz, 1H), 7.36 (t, *J* = 7.4 Hz, 1H). ^13^C NMR (151 MHz, DMSO-*d_6_*) δ 172.6, 168.2, 156.1, 144.6, 142.3, 141.0, 139.8, 137.1, 133.5, 130.7, 129.6, 128.50, 128.4, 127.9, 127.8, 127.3, 125.6, 119.6. HRMS (ESI): calculated [M + H^+^]^+^ 359.8090, found 360.0785

**2-([1,1’-biphenyl]-4-yl)-6-fluoroquinoline-4-carboxylic acid (6c):** Neon yellow powder, yield 75.0%, Rf = 0.13 (6:1 EtOAc-MeOH). ^1^H NMR (600 MHz, DMSO-*d_6_*) δ 8.60 (s, 1H), 8.51–8.43 (m, 1H), 8.38 (d, *J* = 8.3 Hz, 2H), 8.25 (dd, *J* = 9.1, 5.8 Hz, 1H), 7.87 (t, *J* = 7.2 Hz, 2H), 7.77 (d, *J* = 7.5 Hz, 2H), 7.51 (t, *J* = 7.6 Hz, 3H), 7.41 (t, *J* = 7.2 Hz, 1H). ^13^C NMR (151 MHz, DMSO- *d_6_*) δ 167.2, 159.7, 154.9, 145.9, 141.6, 139.3, 136.6, 132.6, 129.0, 127.9, 127.7, 127.4, 127.2, 127.1, 126.7, 124.5, 124.4, 120.4, 120.3, 120.2, 109.3, 109.1. HRMS (ESI): calculated [M + H^+^]^+^ 344.1087, found 344.1084

**2-([1,1’-biphenyl]-4-yl)-8-fluoroquinoline-4-carboxylic acid (6d):** Pale yellow powder, yield 82.3%, Rf = 0.39 (6:1 EtOAc-MeOH). ^1^H NMR (600 MHz, DMSO-*d_6_*) δ 8.59 (d, *J* = 3.5 Hz, 1H), 8.49–8.43 (m, 1H), 8.42–8.37 (m, 2H), 7.90–7.84 (m, 2H), 7.76 (td, *J* = 5.3, 1.5 Hz, 2H), 7.72–7.63 (m, 2H), 7.53–7.47 (m, 2H), 7.44–7.38 (m, 1H) ^13^C NMR (151 MHz, DMSO-*d_6_*) δ 167.8, 160.5, 155.6, 150.5, 141.4, 139.4, 137.5, 137.0, 129.1, 127.9, 127.7, 127.2, 126.8, 126.6, 120.5, 118.7, 116.7, 108.0, 55.6. HRMS (ESI): calculated [M + H^+^]^+^ 344.1087, found 344.1087

**2-([1,1’-biphenyl]-4-yl)-6-bromoquinoline-4-carboxylic acid (6e):** Brown solid, yield 61.6%, Rf = 0.38 (6:1 EtOAc-MeOH). ^1^H NMR (600 MHz, DMSO-*d_6_*) δ 9.03 (s, 1H), 8.78 (d, *J* = 6.0 Hz, 3H), 8.60 (s, 1H), 8.49–8.44 (m, 1H), 8.22 (dd, *J* = 13.7, 9.0 Hz, 2H), 8.10 (d, *J* = 8.2 Hz, 2H), 7.87 (dd, *J* = 8.9, 2.3 Hz, 1H), 7.58–7.53 (m, 2H). ^13^C NMR (151 MHz, DMSO-*d_6_*) δ 167.7, 156.6, 147.5, 134.8, 133.6, 133.0, 132.6, 132.4, 132.1, 131.9, 131.5, 131.3, 129.4, 128.8, 128.7, 128.4, 126.8, 126.5, 125.0, 124.9, 124.3, 121.3.

**2-([1,1’-biphenyl]-4-yl)-8-bromoquinoline-4-carboxylic acid (6f):** Beige powder, yield 87.6%, Rf = 0.26 (6:1 EtOAc-MeOH). ^1^H NMR (600 MHz, DMSO-*d_6_*) δ 8.64 (dd, *J* = 8.3, 1.0 Hz), 8.32–8.28 (m), 8.04 (s), 7.97 (d, *J* = 8.3 Hz), 7.84–7.80 (m), 7.74 (d, *J* = 7.6 Hz), 7.64 (ddd, *J* = 8.2, 7.0, 1.3 Hz), 7.47 (ddd, *J* = 9.3, 6.6, 1.4 Hz), 7.39–7.33 (m).^13^C NMR (151 MHz, DMSO- *d_6_*) δ 173.9, 169.6, 155.6, 151.2, 151.1, 150.1, 148.8, 148.7, 141.3, 140.1, 138.7, 129.6, 129.4, 128.3, 128.3, 128.1, 128.0, 127.6, 127.2, 125.8, 125.4, 116.3.

**2-([1,1’-biphenyl]-4-yl)-6-iodoquinoline-4-carboxylic acid (6g):** Yellow powder, yield 31.7%, Rf = 0.10 (6:1 EtOAc-MeOH). ^1^H NMR (600 MHz, DMSO-*d_6_*) δ 8.64 (d, *J* = 8.8 Hz, 1H), 8.50 (s, 1H), 8.39 (d, *J* = 8.5 Hz, 2H), 8.18 (d, *J* = 8.2 Hz, 1H), 7.86 (dd, *J* = 19.3, 8.4 Hz, 3H), 7.77 (d, *J* = 7.9 Hz, 2H), 7.70 (t, *J* = 7.7 Hz, 1H), 7.51 (t, *J* = 7.7 Hz, 2H), 7.41 (t, *J* = 7.4 Hz, 1H). ^13^C NMR (151 MHz, DMSO-*d_6_*) δ 167.7, 155.4, 148.5, 141.6, 139.4, 138.0, 136.9, 130.4, 129.8, 129.1, 128.0, 127.9, 127.3, 127.0, 126.9, 126.8, 125.5, 123.5, 119.0, 21.8, 18.6, 14.0. HRMS (ESI): calculated [M + H^+^]^+^ 452.0147, found 452.0143

**2-([1,1’-biphenyl]-4-yl)-6-nitroquinoline-4-carboxylic acid (6h):** Dark brown powder, yield 38.3%, Rf = 0.20 (6:1 EtOAc-MeOH). ^1^H NMR (600 MHz, DMSO-*d_6_*) δ 8.33 (s, 1H), 8.28 (dt, *J* = 8.7, 1.6 Hz, 2H), 7.87 (d, 1H), 7.83–7.81 (m, 2H), 7.75 (dt, *J* = 8.3, 1.3 Hz, 2H), 7.68 (d, *J* = 2.6 Hz, 1H), 7.51–7.48 (m, 2H), 7.41–7.38 (m, 1H), 7.26 (dd, *J* = 9.0, 2.6 Hz, 1H). ^13^C NMR (151 MHz, DMSO-*d_6_*) δ 168.8, 149.6, 149.2, 143.7, 140.8, 140.1, 138.1, 133.9, 131.4, 129.6, 128.2, 127.6, 127.4, 127.2, 126.7, 122.8, 119.6, 103.0. HRMS (ESI): calculated [M + H^+^]^+^ 371.1032, found 371.1032

**2-([1,1’-biphenyl]-4-yl)-7-methoxyquinoline-4-carboxylic acid(6i)**: Bright yellow powder, 59.2% (586mg); Rf = 0.31 (6:1 EtOAc-MeOH, Yellow spot with vanillin–sulfuric acid). ^1^H NMR (600 MHz, DMSO-*d_6_*) δ 8.57 (d, *J* = 9.3 Hz, 1H), 8.41–8.37 (m, 2H), 8.35 (s, 1H), 7.90–7.85 (m, 2H), 7.80–7.76 (m, 2H), 7.56 (d, *J* = 2.7 Hz, 1H), 7.54–7.49 (m, 2H), 7.45–7.39 (m, 1H), 7.35 (dd, *J* = 9.3, 2.7 Hz, 1H), 3.98 (s, 3H). ^13^C NMR (151 MHz, DMSO-D6) δ 167.8, 160.5, 155.6, 150.5, 141.4, 139.4, 137.5, 137.0, 129.1, 127.9, 127.7, 127.2, 126.8, 126.6, 120.5, 118.7, 116.7, 108.0, 55.6. HRMS (ESI): calculated [M + H^+^]^+^ 356.1287, found 356.1282

**6-chloro-2-(phenanthren-3-yl)quinoline-4-carboxylic acid (6j):** Yellow powder, yield 36.6%, Rf = 0.31 (6:1 EtOAc-MeOH). ^1^H NMR (600 MHz, DMSO- *d_6_*) δ 9.20 (s, 1H), 8.63 (d, *J* = 13.5 Hz, 2H), 8.42 (s, 1H), 8.07 (d, *J* = 6.9 Hz, 1H), 7.93 (d, *J* = 7.9 Hz, 1H), 7.77 (d, *J* = 7.3 Hz, 1H), 7.64 (d, *J* = 6.2 Hz, 1H), 7.53 (s, 2H), 7.49–7.41 (m, 2H), 7.36 (d, *J* = 6.4 Hz, 2H). ^13^C NMR (151 MHz, DMSO-*d_6_*) δ 167.8, 156.9, 147.5, 137.2, 136.3, 133.2, 132.9, 132.4, 132.4, 131.2, 130.5, 130.4, 129.8, 129.2, 128.7, 127.7, 127.7, 126.9, 126.0, 124.8, 124.8, 123.9, 122.5, 121.5. HRMS (ESI): calculated [M + H^+^]^+^ 384.0791, found 384.0801

**2-(anthracen-2-yl)-6-chloroquinoline-4-carboxylic acid (6k):** Yellow solid, yield 19.8%. Rf = 0.38 (6:1 EtOAc-MeOH). ^1^H NMR (600 MHz, DMSO-*d_6_*) δ 8.96 (s, 1H), 8.73 (s, 1H), 8.62 (s, 1H), 8.56 (s, 1H), 8.46–8.43 (m, 1H), 8.40 (s, 1H), 8.18 (d, *J* = 9.0 Hz, 1H), 7.65 (dd, *J* = 8.6, 1.8 Hz, 1H), 7.53–7.46 (m, 2H). ^13^C NMR (151 MHz, DMSO-*d_6_*) δ 168.4, 156.4, 148.9, 138.9, 136.8, 133.1, 132.4, 130.8, 130.6, 130.5, 130.4, 129.8, 129.2, 128.6, 128.3, 127.7, 127.0, 126.1, 125.9, 123.9, 122.4, 120.0. HRMS (ESI): calculated [M + H^+^]^+^ 384.0791, found 384.0793

**6-chloro-2-(naphthalen-1-yl)quinoline-4-carboxylic acid (6l):** Bright yellow crystals, yield 93.0%, Rf = 0.39 (6:1 EtOAc-MeOH). ^1^H NMR (600 MHz, DMSO-*d_6_*) δ 8.91 (d, *J* = 2.3 Hz, 1H), 8.26 (s, 1H), 8.18 (d, *J* = 9.0 Hz, 1H), 8.14–8.04 (m, 3H), 7.89 (dd, *J* = 9.0, 2.4 Hz, 1H), 7.79 (dd, *J* = 7.1, 1.3 Hz, 1H), 7.66 (dd, *J* = 8.2, 7.0 Hz, 1H), 7.60–7.52 (m, 2H). ^13^C NMR (151 MHz, DMSO-*d_6_*) δ 166.9, 158.9, 146.9, 136.9, 135.5, 133.5, 132.8, 131.9, 130.7, 130.4, 129.6, 128.5, 128.2, 127.0, 126.2, 125.5, 125.1, 125.0, 124.4, 124.2.

**6-chloro-2-(naphthalen-2-yl)quinoline-4-carboxylic acid (6m):** Bright yellow crystals, yield 96.6%, Rf = 0.39 (6:1 EtOAc-MeOH). ^1^H NMR (600 MHz, DMSO-*d_6_*) δ 8.78 (d, *J* = 2.2 Hz, 1H), 8.77 (d, *J* = 2.4 Hz, 1H), 8.59 (s, 1H), 8.41 (dd, *J* = 8.6, 1.8 Hz, 1H), 8.14 (d, *J* = 9.0 Hz, 1H), 8.12–8.08 (m, 1H), 8.05 (d, *J* = 8.6 Hz, 1H), 7.97–7.93 (m, 1H), 7.80 (dd, *J* = 9.0, 2.5 Hz, 1H), 7.61–7.54 (m, 2H). ^13^C NMR (151 MHz, DMSO-*d_6_*) δ 168.2, 156.7, 147.4, 139.9, 135.6, 134.2, 133.5, 132.4, 132.1, 131.0, 129.4, 129.1, 128.1, 127.8, 127.6, 127.2, 125.1, 124.9, 120.5.

**8-chloro-2-(naphthalen-2-yl)quinoline-4-carboxylic acid (6n):** Yellow solid yield 16.5%, Rf = 0.38 (6:1 EtOAc-MeOH). ^1^H NMR (600 MHz, DMSO-*d_6_*) δ 8.90 (s, 1H), 8.72 (s, 1H), 8.57 (ddd, *J* = 15.8, 8.6, 1.3 Hz, 2H), 8.11 (dd, *J* = 15.3, 9.0 Hz, 2H), 8.06–8.02 (m, 1H), 8.01–7.97 (m, 1H), 7.66 (s, 1H), 7.60 (d, *J* = 4.1 Hz, 2H). ^13^C NMR (151 MHz, DMSO-*d_6_*) δ 168.0, 156.5, 144.7, 139.4, 135.4, 134.3, 133.7, 133.5, 130.9, 129.5, 129.2, 128.3, 128.2, 128.0, 127.9, 127.3, 125.5, 125.4, 124.9, 120.4. HRMS (ESI): calculated [M + H^+^]^+^ 334.0635, found 334.0626

**2-(anthracen-2-yl)-8-chloroquinoline-4-carboxylic acid (6o):** Yellow solid, yield 28.9%. Rf = 0.40 (6:1 EtOAc-MeOH). ^1^H NMR (600 MHz, DMSO-*d_6_*) δ 9.69 (s, 1H), 9.14 (d, *J* = 8.3 Hz, 1H), 8.83 (s, 1H), 8.65–8.61 (m, 2H), 8.27 (d, *J* = 8.3 Hz, 1H), 8.18 (d, *J* = 8.3 Hz, 1H), 8.04 (d, *J* = 8.5 Hz, 1H), 7.94 (s, 2H), 7.88 (s, 1H), 7.78 (t, *J* = 8.1 Hz, 1H), 7.72 (dt, *J* = 14.0, 7.3 Hz, 2H). ^13^C NMR (151 MHz, DMSO-*d_6_*) δ 167.7, 167.6, 156.6, 151.6, 147.5, 134.8, 133.2, 133.0, 132.3, 132.1, 131.5, 131.3, 130.8, 129.4, 128.8, 128.7, 128.4, 126.5, 125.8, 125.0, 124.3, 123.9, 121.3, 79.7. HRMS (ESI): calculated [M + H^+^]^+^ 384.0791, found 384.0790

**8-fluoro-2-(naphthalen-1-yl)quinoline-4-carboxylic acid (6p):** Yellow powder, yield 7.5%, Rf = 0.22 (6:1 EtOAc-MeOH). ^1^H NMR (600 MHz, DMSO-*d_6_*) δ 8.50–8.46 (m), 8.16 (dd, *J* = 25.3, 8.7 Hz), 8.14 (d, *J* = 8.8 Hz), 8.05 (d, *J* = 8.1 Hz), 7.97 (d, *J* = 2.4 Hz), 7.72 (t, *J* = 7.5 Hz), 7.49 (d, *J* = 7.9 Hz). ^13^C NMR (151 MHz, DMSO-*d_6_*) δ 168.5, 159.05, 157.0, 155.2, 140.9, 134.0, 131.0, 129.9, 129.6, 129.0, 128.6, 127.4, 126.7, 126.0, 125.7, 123.5, 123.0, 117.8, 114.3, 110.4, 99.0. HRMS (ESI): calculated [M + H^+^]^+^ 318.0930, found 318.0931

**6-bromo-2-(naphthalen-2-yl)quinoline-4-carboxylic acid (6q):** Brown solid, yield 26.8%, Rf = 0.65 (6:1 EtOAc-MeOH). ^1^H NMR (600 MHz, DMSO-*d_6_*) δ 8.89 (s, 1H), 8.70–8.67 (m, 1H), 8.67 (s, 1H), 8.51 (dd, *J* = 8.6, 1.8 Hz, 1H), 8.22 (d, *J* = 8.1 Hz, 1H), 8.15 (dd, *J* = 6.0, 3.4 Hz, 1H), 8.09 (d, *J* = 8.6 Hz, 1H), 7.99 (dq, *J* = 6.4, 3.2 Hz, 1H), 7.86 (dd, *J* = 8.3, 1.3 Hz, 1H), 7.76–7.69 (m, 1H), 7.60 (dt, *J* = 6.3, 3.5 Hz, 2H). ^13^C NMR (151 MHz, DMSO-*d_6_*) δ 168.2, 156.2, 149.0, 138.3, 135.8, 134.2, 133.6, 130.8, 130.3, 129.4, 129.0, 128.3, 128.1, 127.7, 127.6, 127.2, 126.0, 125.0, 124.0, 119.9.

**6-bromo-2-(phenanthren-3-yl)quinoline-4-carboxylic acid (6r):** Brown solid yield 86.0%. Rf = 0.16 (6:1 EtOAc-MeOH). ^1^H NMR (600 MHz, DMSO-*d_6_*) δ 8.66 (d, *J* = 8.0 Hz, 1H), 8.52 (s, 1H), 8.41 (d, *J* = 8.4 Hz, 2H), 8.19 (d, *J* = 8.3 Hz, 1H), 7.87 (dd, *J* = 15.6, 8.4 Hz, 3H), 7.79–7.75 (m, 2H), 7.73–7.69 (m, 1H), 7.51 (t, *J* = 7.7 Hz, 2H), 7.42 (t, *J* = 7.4 Hz, 1H). ^13^C NMR (151 MHz, DMSO-*d_6_*) δ 168.2, 155.9, 149.0, 142.1, 139.9, 138.3, 137.4, 130.8, 130.3, 129.6, 128.5, 128.3, 128.3, 127.7, 127.3, 126.0, 124.0, 119.6. HRMS (ESI): calculated [M + H^+^]^+^ 428.0286, found 428.0285

**2-([1,1’-biphenyl]-4-yl)-6-methylquinoline-4-carboxylic acid (7a):** Beige powder, yield 63.8%, Rf = 0.33 (6:1 EtOAc-MeOH). ^1^H NMR (600 MHz, DMSO-*d_6_*) δ 8.47 (s, 1H), 8.43, (s, 1H), 8.39–8.34 (m, 2H), 8.07 (d, *J* = 8.5 Hz, 1H), 7.88–7.83 (m, 2H), 7.79–7.74 (m, 2H), 7.69 (dd, *J* = 8.7, 2.1 Hz, 1H), 7.53–7.47 (m, 2H), 7.44–7.38 (m, 1H), 2.54 (s, 3H). ^13^C NMR (151 MHz, DMSO-*d_6_*) δ 168.3, 154.9, 147.7, 141.8, 139.9, 138.0, 137.5, 137.4, 132.9, 130.1, 129.6, 128.4, 128.15, 127.7, 127.2, 124.6, 124.0, 119.5, 22.2.

**2-([1,1’-biphenyl]-4-yl)-6-ethylquinoline-4-carboxylic acid (7b):** Yellow powder, yield 57.5%, Rf = 0.10 (6:1 EtOAc-MeOH). ^1^H NMR (600 MHz, DMSO-*d_6_*) δ 8.58 (s), 8.26 (d, *J* = 8.2 Hz), 7.93 (d, *J* = 8.5 Hz), 7.66 (d, *J* = 7.5 Hz), 7.55 (d, *J* = 9.9 Hz), 7.44 (t, *J* = 7.7 Hz), 7.35 (t, *J* = 7.3 Hz), 2.76–2.59 (m), 1.18 (t, *J* = 7.3 Hz). ^13^C NMR (151 MHz, DMSO- *d_6_*) δ 171.0, 154.7, 147.7, 146.9, 142.0, 141.2, 140.0, 138.3, 130.7, 129.6, 129.5, 128.3, 128.0, 128.0, 127.8, 127.5, 127.1, 125.4, 125.3, 125.3, 125.1, 117.7, 29.1, 16.1. HRMS (ESI): calculated [M + H^+^]^+^ 354.1494, found 354.1491

**2-([1,1’-biphenyl]-4-yl)-6-isopropylquinoline-4-carboxylic acid (7c):** Beige powder, yield 39.8%, Rf = 0.15 (6:1 EtOAc-MeOH). ^1^H NMR (600 MHz, MeOH-*d_4_*) δ 8.61 (s, 1H), 8.45 (s, 1H), 8.26 (d, *J* = 8.3 Hz, 2H), 8.13 (d, *J* = 8.7 Hz, 1H), 7.83 (d, *J* = 8.3 Hz, 2H), 7.78 (dd, *J* = 8.7, 2.0 Hz, 1H), 7.72 (d, *J* = 7.3 Hz, 2H), 7.48 (t, *J* = 7.7 Hz, 2H), 7.38 (t, *J* = 7.4 Hz, 1H), 3.19–3.12 (m, 1H), 1.67 (d, *J* = 15.6 Hz, 1H). ^13^C NMR (151 MHz, MeOH-*d_4_*) δ 170.4, 169.8, 157.1, 149.9, 149.0, 143.9, 141.6, 138.7, 131.2, 130.2, 130.1, 130.0, 129.1, 128.8, 128.5, 128.2, 128.2, 128.0, 127.9, 125.5, 123.2, 121.1, 35.8, 24.2. HRMS (ESI): calculated [M + H^+^]^+^ 368.1651, found 368.1650

**2-([1,1’-biphenyl]-4-yl)-6-butylquinoline-4-carboxylic acid (7d):** Yellow powder, yield 34.5%, Rf = 0.20 (6:1 EtOAc-MeOH). ^1^H NMR (600 MHz, MeOH-*d_4_*) δ 8.40 (s, 1H), 8.26 (s, 1H), 8.23 (d, *J* = 8.2 Hz, 1H), 7.82 (d, *J* = 8.2 Hz, 1H), 7.75 (d, *J* = 8.5 Hz, 1H), 7.30 (t, *J* = 7.3 Hz, 1H), 2.87–2.81 (m, 1H), 1.78–1.70 (m, 1H), 1.48–1.39 (m, 1H), 0.98 (t, *J* = 7.4 Hz, 1H). ^13^C NMR (151 MHz, MeOH-*d_4_*) δ 157.3, 147.3, 143.7, 141.7, 141.1, 137.1, 132.7, 130.1, 130.1, 130.0, 129.8, 129.4, 129.1, 128.8, 128.5, 128.2, 128.2, 128.0, 127.9, 127.0, 125.6, 36.9, 34.7, 26.7, 23.4, 14.3. HRMS (ESI): calculated [M + H^+^]^+^ 382.1807, found 382.1802

**6-methyl-2-(naphthalen-2-yl)quinoline-4-carboxylic acid (7e):** Yellow solid, yield 28.0%, Rf = 0.11 (6:1 EtOAc-MeOH. ^1^H NMR (600 MHz, DMSO-*d_6_*) δ 8.80 (s, 1H), 8.56 (s, 1H), 8.45–8.38 (m, 2H), 8.11–8.01 (m, 3H), 7.96–7.91 (m, 1H), 7.65 (d, *J* = 8.6 Hz, 1H), 7.56–7.51 (m, 2H). ^13^C NMR (151 MHz, DMSO-*d_6_*) δ 168.3, 155.2, 147.7, 138.0, 137.5, 135.9, 134.1, 133.6, 132.9, 130.1, 129.4, 129.0, 128.1, 127.6, 127.3, 127.1, 124.9, 124.7, 124.0, 119.8, 22.2.

**6-methyl-2-(naphthalen-1-yl)quinoline-4-carboxylic acid (7f):** Beige powder, yield 7.5%, Rf = 0.17 (6:1 EtOAc-MeOH). ^1^H NMR (600 MHz, DMSO-*d_6_*) δ 8.52 (s, 1H), 8.12 (d, *J* = 8.5 Hz, 1H), 8.09–7.91 (m, 4H), 7.76–7.70 (m, 1H), 7.70–7.61 (m, 2H), 7.59–7.54 (m, 1H), 7.54–7.48 (m, 1H), 2.56 (d, *J* = 3.8 Hz, 3H). ^13^C NMR (151 MHz, DMSO-*d_6_*) δ 168.7, 157.8, 147.5, 138.2, 137.5, 134.0, 132.5, 131.1, 129.8, 129.6, 129.0, 128.4, 127.3, 126.6, 126.0, 125.8, 125.3, 125.2, 124.1, 123.1, 22.2.

**6-ethyl-2-(naphthalen-2-yl)quinoline-4-carboxylic acid (7g):** Brown powder, yield 25.2%, Rf = 0.13 (6:1 EtOAc-MeOH). ^1^H NMR (600 MHz, DMSO-*d_6_*) δ 8.83 (s), 8.63 (s), 8.60 (s), 8.49–8.40 (m), 8.05 (d, *J* = 8.6 Hz), 7.86–7.81 (m), 7.72 (d, *J* = 8.6 Hz), 7.65–7.60 (m), 2.81 (dd, *J* = 15.1, 7.5 Hz), 1.26 (t, *J* = 7.6 Hz). ^13^C NMR (151 MHz, DMSO-*d_6_*) δ 168.3, 155.3, 147.9, 145.5, 144.1, 137.6, 135.8, 134.1, 133.6, 132.7, 131.8, 130.1, 128.8, 128.2, 127.3, 126.5, 124.9, 124.0, 123.7, 68.7, 29.1, 15.9. HRMS (ESI): calculated [M + H^+^]^+^ 328.1338, found 328.1335

**6-isopropyl-2-(naphthalen-2-yl)quinoline-4-carboxylic acid (7h):** White powder, yield 45.6%, Rf = 0.13 (6:1 EtOAc-MeOH). ^1^H NMR (600 MHz, MeOH-*d_4_*) δ 8.58 (s, 1H), 8.31–8.24 (m, 2H), 8.14 (s, 1H), 8.07 (d, *J* = 8.7 Hz, 1H), 7.93 (dd, *J* = 9.1, 4.8 Hz, 2H), 7.69 (ddd, *J* = 10.8, 8.7, 1.9 Hz, 2H), 7.50 (d, *J* = 4.4 Hz, 1H), 6.91 (dd, *J* = 8.0, 1.7 Hz, 2H), 6.69 (d, *J* = 8.1 Hz, 1H), 2.82–2.71 (m, 3H), 1.37 (d, *J* = 7.0 Hz, 6H). ^13^C NMR (151 MHz, MeOH-*d_4_*) δ 150.1, 148.7, 138.2, 135.3, 130.7, 129.8, 129.7, 129.5, 128.9, 128.7, 128.3, 127.9, 127.5, 126.1, 124.2, 124.0, 119.4, 118.5, 118.1, 35.7, 34.8, 24.7, 24.3. HRMS (ESI): calculated [M + H^+^]^+^ 342.1494, found 342.1504

**6-1-butyl-2-(naphthalen-2-yl)quinoline-4-carboxylic acid (7i):** Yellow powder, yield 33.3%, Rf = 0.13 (6:1 EtOAc-MeOH). ^1^H NMR (600 MHz, DMSO-*d_6_*) δ 8.75 (s, 1H), 8.44 (dd, *J* = 8.7, 1.3 Hz, 2H), 8.17 (s, 1H), 8.13 (d, *J* = 7.4 Hz, 2H), 8.05 (d, *J* = 8.6 Hz, 1H), 7.89–7.81 (m, 2H), 7.67 (t, *J* = 6.9 Hz, 1H), 7.62 (t, *J* = 7.4 Hz, 1H), 2.76 (t, *J* = 7.7 Hz, 2H), 1.65 (dt, *J* = 15.1, 7.6 Hz, 2H), 1.40–1.33 (m, 3H), 0.93 (t, *J* = 7.4 Hz, 3H). ^13^C NMR (151 MHz, DMSO-*d_6_*) δ 133.3, 133.1, 130.3, 129.5, 128.8, 128.8, 128.6, 128.2, 128.2, 127.7, 127.6, 127.5, 126.9, 126.7, 126.4, 126.2, 125.9, 124.6, 123.5, 123.2, 35.2, 33.1, 21.9, 13.8. HRMS (ESI): calculated [M + H^+^]^+^ 356.1651, found 356.1658

**6-methyl-2-(phenanthren-3-yl)quinoline-4-carboxylic acid (7j):** Yellow solid, yield 78.6%, Rf = 0.16 (6:1 EtOAc-MeOH) ^1^H NMR (600 MHz, CDCl_3_) δ 9.53 (d, *J* = 1.3 Hz, 1H), 8.91 (d, *J* = 8.2 Hz, 1H), 8.85 (d, *J* = 2.3 Hz, 1H), 8.64 (s, 1H), 8.40 (d, *J* = 1.7 Hz, 1H), 8.24 (d, *J* = 8.9 Hz, 1H), 8.05 (dd, *J* = 8.3, 5.0 Hz 1H), 7.93 (d, *J* = 7.0 Hz, 1H), 7.85–7.77 (m, 2H), 7.74 (ddd, *J* = 8.3, 5.2, 1.8 Hz, 2H), 7.65 (td, *J* = 7.4, 7.0, 1.0 Hz, 1H), 4.60 (q, *J* = 7.2 Hz, 2H), 1.55 (t, *J* = 7.2 Hz, 3H). ^13^C NMR (151 MHz, CDCl_3_) δ 166.1, 157.1, 147.9, 136.4, 135.2, 134.0, 133.1, 132.4, 131.9, 131.0, 130.7 130.6, 129.4, 128.8, 128.3, 127.0, 127.0, 126.6, 125.5, 124.8, 123.0, 122.2, 121.4, 62.3, 14.5. HRMS (ESI): calculated [M + H^+^]^+^ 364.1338, found 364.1339

**2-(anthracen-2-yl)-6-methylquinoline-4-carboxylic acid (7k):** Yellow solid, yield 59.6%, Rf = 0.15 (6:1 EtOAc-MeOH). ^1^H NMR (600 MHz, DMSO-*d_6_*) δ 8.96 (s, 1H), 8.73 (s, 1H), 8.62 (s, 1H), 8.56 (s, 1H), 8.46–8.43 (m, 1H), 8.40 (s, 1H), 8.18 (d, *J* = 9.0 Hz, 1H), 7.65 (dd, *J* = 8.6, 1.8 Hz, 1H), 7.53–7.46 (m, 2H). ^13^C NMR (151 MHz, DMSO-*d_6_*) δ 168.4, 156.4, 148.9, 138.9, 136.8, 133.1, 132.4, 130.8, 130.6, 130.5, 130.4, 129.8, 129.2, 128.6, 128.3, 127.7, 127.0, 126.1, 125.9, 123.9, 122.4, 120.0. HRMS (ESI): calculated [M + H^+^]^+^ 364.1338, found 364.1338

**6-ethyl-2-(phenanthren-3-yl)quinoline-4-carboxylic acid (7l):** Yellow powder, yield 14.1%, Rf = 0.10 (6:1 EtOAc-MeOH). ^1^H NMR (600 MHz, DMSO-*d_6_*) δ 9.55 (s), 9.00 (d, *J* = 8.3 Hz), 8.51 (s), 8.37 (s), 8.08 (d, *J* = 8.3 Hz), 8.03–7.96 (m), 7.87 (s), 7.71 (t, *J* = 7.5 Hz), 7.64 (t, *J* = 7.4 Hz), 2.75 (q, *J* = 7.6 Hz), 1.24 (t, *J* = 7.6 Hz). ^13^C NMR (151 MHz, DMSO-*d_6_*) δ 170.7, 155.3, 147.7, 141.5, 138.0, 132.6, 132.4, 130.5, 130.3, 129.5, 129.1, 128.1, 127.6, 127.6, 127.1, 127.0, 126.1, 126.0, 125.8, 125.6, 125.3, 123.7, 121.6, 79.5, 29.1, 16.1. HRMS (ESI): calculated [M + H^+^]^+^ 378.1494, found 378.1492

**6-isopropyl-2-(phenanthren-3-yl)quinoline-4-carboxylic acid (7m):** Yellow powder, yield 51.0%, Rf = 0.18 (6:1 EtOAc-MeOH). ^1^H NMR (600 MHz, DMSO-*d_6_*) δ 9.59 (s, 1H), 9.05 (d, *J* = 8.3 Hz, 1H), 8.65 (s, 1H), 8.52 (dd, *J* = 8.3, 1.5 Hz, 1H), 8.39 (s, 1H), 8.16 (dd, *J* = 12.7, 8.5 Hz, 3H), 8.01 (d, *J* = 8.4 Hz, 1H), 7.91 (s, 2H), 7.76 (t, *J* = 7.6 Hz, 1H), 7.69 (t, *J* = 7.4 Hz, 1H), 1.30 (d, *J* = 6.9 Hz, 6H), 1.25–1.14 (m, 2H). ^13^C NMR (151 MHz, DMSO-*d_6_*) δ 155.7, 148.5, 147.9, 136.9, 132.9, 132.3, 130.5, 130.4, 130.4, 130.2, 129.8, 129.2, 128.6, 127.8, 126.9, 126.0, 124.0, 123.7, 122.0, 119.6, 79.4, 56.7, 34.3, 33.9, 24.1, 22.2, 18.8, 14.4. HRMS (ESI): calculated [M + H^+^]^+^ 392.1651, found 392.1654

**6-butyl-2-(phenanthren-3-yl)quinoline-4-carboxylic acid (7n):** Yellow powder, yield 37.3%, Rf = 0.20 (6:1 EtOAc-MeOH). ^1^H NMR (600 MHz, MeOH-*d_4_*) δ 9.48 (s, 1H), 8.95 (d, *J* = 8.3 Hz, 1H), 8.33 (dd, *J* = 8.2, 1.4 Hz, 1H), 8.26 (s, 1H), 8.07 (dd, *J* = 20.4, 8.4 Hz, 2H), 7.94 (d, *J* = 8.1 Hz, 1H), 7.82 (s, 2H), 7.74–7.70 (m, 1H), 7.64 (dd, *J* = 7.9, 2.6 Hz, 2H), 2.85–2.81 (m, 2H), 1.73 (dt, *J* = 15.2, 7.6 Hz, 3H), 1.43 (dq, *J* = 14.3, 7.2 Hz, 3H), 0.97 (t, *J* = 7.4 Hz, 4H). ^13^C NMR (151 MHz, MeOH-*d_4_*) δ 158.2, 148.7, 142.7, 138.9, 134.1, 133.8, 132.5, 132.4, 132.0, 131.7, 130.2, 129.7, 129.6, 129.0, 128.1, 128.0, 127.5, 126.9, 126.2, 125.4, 124.0, 123.2, 118.6, 36.9, 34.7, 23.5, 23.3, 15.4, 14.3. HRMS (ESI): calculated [M + H^+^]^+^ 406.1807, found 406.1808

### 3.3. Antitubercular Assay

#### 3.3.1. Microplate Alamar Blue Assay

MABA cell stock preparation: *Mtb* H_37_Rv seed stock (ATCC 27294) was prepared by suspending colonies from 7H11 agar in 200 mL 7H9 broth in a 500 mL Nephelo flask that were then grown until log phase of 40–60 Klett units. The culture was transferred into centrifuge tubes and was centrifuged at 4000 RCF at 4 °C. The supernatant was discarded, and 1 mL of sterile phosphate buffer saline (PBS) was added into each of the centrifuge tubes. The pellet was resuspended by pipetting, and each tube was filled with 30 mL PBS. The pellets were resuspended by vortex. The supernatant was discarded, and then another 1 mL of the PBS was transferred into each centrifuge tube. The pellets were resuspended again by pipetting. An aliquot of 200 µL of the suspended cells was transferred into a 1.5 mL sterile screwcap microtube. The seed stock was stored at −80 °C.

The antitubercular inhibitory activity of the compounds against *Mtb* H_37_Rv (American Type Culture Collection, Rockville, MD, USA) was determined using a fluorescence reading at a 530 nm excitation wavelength and 590 nm emission wavelength in the Microplate Alamar Blue Assay (MABA), with Rifampin (MIC = 0.05 μM), isoniazid (MIC = 0.25 μM), linezolid (MIC = 1.16 μM), and bedaquiline (MIC = 0.23 μM) as positive drug controls. A 12.8 mM stock solution of the test compounds was first acquired by solubilizing the compounds in DMSO, and then it was diluted to its final test concentrations, which ranged from 128.0 µM to 0.5 µM. Then, in a Middlebrook 7H12 medium (7H9 broth constituted by 0.1% *w*/*v* casitone, 5.6 µg/mL of palmitic acid, 5 mg/mL bovine serum albumin, and 4mg/mL catalase, filter-sterilized), two-fold dilutions of the compounds were prepared in a 100 µL 96-well microplate. The *Mtb* strains were added to the microplate wells with a volume of 100 µL inoculum that contains 2 × 10^5^ cfu/mL, resulting in a total volume of 200 µL. This microplate was then incubated at 37 °C for seven days. Then, 12.5 µL of Tween 80 and 20 µL of Alamar Blue (Trek Diagnostic, Westlake, OH, USA) were added to each well on the seventh day and incubated for another 16–24 h. The fluorescence measurements were taken to obtain MIC_90_ values based on the average of two individual measurements of the wells. The inhibitory concentration was determined by looking at the lowest concentration exhibiting a 90% fluorescence inhibition compared to the untreated bacterial control [26].

#### 3.3.2. Low Oxygen Recovery Assay

LORA cell stock preparation: *Mtb* reporter strain harboring pFCA-luxAB from a 7H11 agar and was inoculated into 20 mL Middlebrook 7H9 medium with kanamycin. The culture was incubated at 37 °C (150) rpm for 5 days. From the culture, 2 mL was transferred into 7H9 medium plus kanamycin. The cultures were incubated for another 5–7 days. An aliquot of 1 mL of the cell suspension was transferred into tubes and stored at −80 °C. Then, an amount of 100 µL was taken from the stock, and serial dilutions were prepared (10^−4^ to 10^−6^), which were plated on 7H11 plates. After that, 770 mL of Dubos broth medium with kanamycin and methylene blue was inoculated with a volume of the seed stock in a culture flask. The culture flask was stirred at 120 rpm at 37 °C for 10–13 days. Culture cells were harvested by centrifugation. The pellet was resuspended in 1 mL of PBS, diluted with 30 mL of PBS, and centrifuged. The supernatant was discarded. The pellet was resuspended again in 1 mL of PBS. The seed stock was stored at −80 °C.

The LORA assay was performed under anaerobic conditions, with oxygen concentrations of less than 0.16%; this condition for microplate cultures was achieved by using Anoxomat model WS-8080, followed by subsequent cycling of air evacuation and filling of 10% H_2_ and 5% CO_2_ and then balanced with N_2_ gas thrice. The anaerobic conditions were confirmed upon the use of an anaerobic indicator strip. The plates were then incubated for 10 days at 37 °C and were recovered by transferring to a 5% CO_2_-enriched air incubator for 28 h. On the 11th day, 100 µL of the cultures was pipetted into 96-well microtiter plates. On the other hand, the microplate cultures for the aerobic assay were incubated in ambient gas conditions for 7 days, with the procedures performed for luminescence reading. In both microtiter wells, 100 µL of freshly prepared 10-fold dilution in PBS of *n*-decanal aldehyde from Sigma in ethanol was added, and the luminescence of these wells were read with a reading time of 1 s with a Victor multilabel reader from Perkin-Elmer Life Sciences. Rifampin (MIC = 0.22 μM), isoniazid (MIC > 128 μM), linezolid (MIC = 1.75 μM), and bedaquiline (MIC = 0.41 μM) were used as positive drug controls [27].

#### 3.3.3. DNA Gyrase Inhibition

A MIX solution of the assay was prepared: relaxed pBR322 (0.5 μL) and water 17.5 μL per assay. A total of 3 μL of 0.01 μM, 1.0 μM, and 100 μM test compounds was added into each assay and mixed gently. The enzyme was diluted in a dilution buffer (1:30), and then 3 μL was transferred into each assay. The sample mixtures were mixed and incubated for 60 min at 37 °C. This was followed by the addition of 30 μL of STEB (40 % (*w*/*v*) sucrose, 100 mM of Tris-HCl pH8, 10 mM of EDTA, and 0.5 mg/mL of Bromophenol Blue) and 30 μL of chloroform/isoamyl alcohol (*v*:*v*, 24:1). The mixtures were then mixed and centrifuged. After that, 20 μL of aqueous (upper blue) phase was loaded onto a 1% (*w*/*v*) agarose gel. Electrophoresis was then run at 70V for approximately 90 min. The obtained gel was stained with 50 µg/mL ethidium bromide in water and destained in water. Samples were viewed with a transilluminator or gel documentation system for analysis [28].

### 3.4. Molecular Docking Analysis

Quinoline-4-carboxylic acid derivatives were subjected to molecular docking simulations with the *Mtb* DNA gyrase binding site (PDB ID: 5BTN) to assess their binding characteristics. The enzymes were fetched from the protein data bank as co-crystallized structures. USCF Chimera (version 1.13.1) was used to facilitate the removal of bound residues and the minimization of structures. Dock-prepping of ligand and protein structures was performed using Antechamber, and molecular docking was performed using the BFGS algorithm of AutoDock Vina (version 1.1.2). Validation of the docking protocol was performed via a redocking experiment of the co-crystallized ligand, 5-methylmoxifloxacin. The conformational protein–ligand structure was visualized and analyzed using Biovia Discovery Studios (version 4.1) [29,30,31].

## 4. Conclusions

In summary, we can assert that quinoline carboxylic acids are endowed with anti-mycobacterial activity. Surprisingly, similar to what happens in the case of fluoroquinolones, the presence of substituents such as halogens and alkyl groups in the classic 6 position of fluoroquinolones involves an increase in antitubercular activity in vitro against fast and non-replicating *Mtb*. Out of the 48 compounds synthesized, 6-1-butyl-2-(naphthalen-2-yl)quinoline-4-carboxylic acid (**7i**) and 6-isopropyl-2-(phenanthren-3-yl)quinoline-4-carboxylic acid (**7m**) showed antitubercular activity. Both derivatives exhibited inhibition against *Mtb* DNA gyrase in vitro and in silico.

## Figures and Tables

**Table 1:** MABA minimum inhibitory concentration (MIC_90_) data of **1a–1p** versus *Mtb* H_37_Rv.

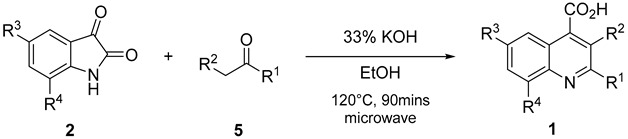					
Compound	R^1^	R^2^	R^3^	R^4^	MABA (µg/mL)
**1a**	1-pentyl	H	H	H	>64
**1b**	phenethyl	H	H	H	>64
**1c**	benzyl	phenyl	H	H	>64
**1d**	benzhydryl	H	H	H	>64
**1e**	methyl	H	methyl	H	>64
**1f**	methyl	H	Cl	H	>64
**1g**	phenyl	H	H	H	>64
**1h**	2-([1,1’-biphenyl]-4-yl)	H	H	H	62.57
**1i**	2-(naphthalen-1-yl)	H	H	H	>64
**1j**	2-(naphthalen-2-yl)	H	H	H	>64
**1k**	2-(anthracen-2-yl)	H	H	H	>64
**1l**	2-(phenanthren-3-yl)	H	H	H	>64
**1m**	2-(2,3-dihydrobenzo[b][1,4]dioxin-6-yl)	H	H	H	>64
**1n**	2-(4-methoxyphenyl)	H	H	H	>64
**1o**	2-(4-fluoro-3-nitrophenyl)	H	H	H	>64
**1p**	2-(2,3-dihydrobenzo[b][1,4]dioxin-6-yl)	H	H	F	>64
				RIF	0.05
				INH	0.25
				LZD	1.16
				BFQ	0.23

**Table 2:** MABA and LORA minimum inhibitory concentration (MIC_90_) data of **6a–6r** versus *Mtb* H_37_Rv.

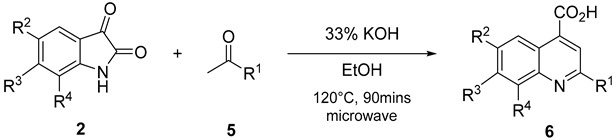						
Compound	R^1^	R^2^	R^3^	R^4^	MABA (µg/mL)	LORA (µg/mL)
**6a**	2-([1,1’-biphenyl]-4-yl)	Cl	H	H	30.45	31.21
**6b**	2-([1,1’-biphenyl]-4-yl)	H	H	Cl	61.08	-
**6c**	2-([1,1’-biphenyl]-4-yl)	F	H	H	31.65	39.52
**6d**	2-([1,1’-biphenyl]-4-yl)	H	H	F	62.49	-
**6e**	2-([1,1’-biphenyl]-4-yl)	Br	H	H	31.90	>64
**6f**	2-([1,1’-biphenyl]-4-yl)	H	H	Br	>64	-
**6g**	2-([1,1’-biphenyl]-4-yl)	I	H	H	>64	-
**6h**	2-([1,1’-biphenyl]-4-yl)	NO_2_	H	H	63.34	-
**6i**	2-([1,1’-biphenyl]-4-yl)	H	MeO	H	>64	-
**6j**	2-(phenanthren-3-yl)	Cl	H	H	52.01	-
**6k**	2-(anthracen-2-yl)	Cl	H	H	60.78	-
**6l**	2-(naphthalen-1-yl)	Cl	H	H	>64	-
**6m**	2-(naphthalen-2-yl)	Cl	H	H	55.68	-
**6n**	2-(naphthalen-2-yl)	H	H	Cl	60.02	-
**6o**	2-(anthracen-2-yl)	H	H	Cl	60.18	-
**6p**	2-(naphthalen-1-yl)	H	H	F	>64	-
**6q**	2-(naphthalen-2-yl)	Br	H	H	>32	>32
**6r**	2-(phenanthren-3-yl)	Br	H	H	>32	>32
				RIF	0.05	0.22
				INH	0.25	>128
				LZD	1.16	1.75
				BFQ	0.23	0.41

**Table 3:** MABA and LORA minimum inhibitory concentration (MIC_90_) data of **7a–7n** versus *Mycobacterium tuberculosis* H_37_Rv.

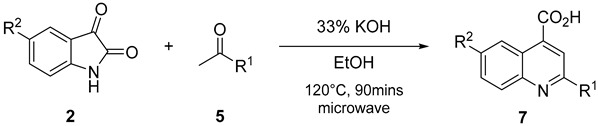				
Compound	R^1^	R^2^	MABA (µg/mL)	LORA (µg/mL)
**7a**	2-([1,1’-biphenyl]-4-yl)	Methyl	29.67	30.94
**7b**	2-([1,1’-biphenyl]-4-yl)	Ethyl	61.78	-
**7c**	2-([1,1’-biphenyl]-4-yl)	isopropyl	27.80	28.79
**7d**	2-([1,1’-biphenyl]-4-yl)	1-butyl	>32	19.84
**7e**	2-(naphthalen-2-yl)	Methyl	58.77	-
**7f**	2-(naphthalen-1-yl)	Methyl	>64	-
**7g**	2-(naphthalen-2-yl)	Ethyl	>64	-
**7h**	2-(naphthalen-2-yl)	isopropyl	>64	-
**7i**	2-(naphthalen-2-yl)	1-butyl	>16	>16
**7j**	2-(phenanthren-3-yl)	Methyl	31.26	>64
**7k**	2-(anthracen-2-yl)	Methyl	30.96	>64
**7l**	2-(phenanthren-3-yl)	Ethyl	51.63	-
**7m**	2-(phenanthren-3-yl)	isopropyl	>16	>16
**7n**	2-(phenanthren-3-yl)	1-butyl	30.78	59.36
		RIF	0.05	0.22
		INH	0.25	>128
		LZD	1.16	1.75
		BFQ	0.23	0.41

**Table 4:** Binding energies of QCA **7i** and **7m** against *Mtb* DNA Gyrase.

Compound	MABA (µg/mL)	LORA (µg/mL)	Docking Score Against *Mtb* DNA Gyrase (kcal/mol)
**7i**	>16	>16	−9.7
**7m**	>16	>16	−10.4

## Data Availability

The data that support the findings of this study are available from the corresponding author upon reasonable request.

## Supplementary Materials

The following supporting information can be downloaded at https://www.mdpi.com/article/10.3390/ijms241411632/s1.
