# Supplementary material for: Mycobacterium tuberculosis Inhibitors Based on Arylated Quinoline Carboxylic Acid Backbones with Anti-Mtb Gyrase Activity
Source: Int J Mol Sci. 2023 Jul 19;24(14):11632. doi: 10.3390/ijms241411632 (PMC10380224; doi:10.3390/ijms241411632)
Supplement: Supplementary file 1 [file ijms-24-11632-s001.zip › ijms-2454089-supplementary.pdf]

Supporting Information for

Mycobacterium tuberculosis inhibitors based on arylated quinoline carboxylic acid backbones with anti-*Mtb* gyrase activity

Mark Tristan J. Quimque, Adrian D. Go, Justin Allen K. Lim, Warren S. Vidar, and Allan Patrick G. Macabeo*

[1]The Graduate School, University of Santo Tomas, España Blvd., Manila 1015 Philippines
[2]Laboratory for Organic Reactivity, Discovery and Synthesis (Rm. 410) Research Center for the Natural and Applied Sciences University of Santo Tomas Espana Blvd., Manila 1015, Philippines
[3]Chemistry Department, College of Science and Mathematics, Mindanao State University – Iligan Institute of Technology, Tibanga, Iligan City, Philippines

*Corresponding author: A. P. G. Macabeo (agmacabeo@ust.edu.ph)

TABLE OF CONTENTS

Figure S1. 600 MHz ^{1}H NMR spectrum of **1d** (DMSO-d_6)....4
Figure S2. 151 MHz ^{13}C NMR spectrum of **1d** (DMSO-d_6)....4
Figure S3. 600 MHz ^{13}H NMR spectrum of **1o** (DMSO-d_6)....5
Figure S4. 151 MHz ^{13}C NMR spectrum of **1o** (DMSO-d_6)....5
Figure S5. 600 MHz ^{1}H NMR spectrum of **1p** (MeOH-d_4)....6
Figure S6. 151 MHz ^{13}C NMR spectrum of **1p** (MeOH-d_4)....6
Figure S7. 600 MHz ^{1}H NMR spectrum of **6b** (DMSO-d_6)....7
Figure S8. 151 MHz ^{13}C NMR spectrum of **6b** (DMSO-d_6)....7
Figure S9. 600 MHz ^{13}C NMR spectrum of **6c** (DMSO-d_6)....8
Figure S10. 151 MHz ^{13}C NMR spectrum of **6c** (DMSO-d_6)....8
Figure S11. 600 MHz ^{1}H NMR spectrum of **6d** (DMSO-d_6)....9
Figure S12. 151 MHz ^{13}C NMR spectrum of **6d** (DMSO-d_6)....9
Figure S13. 600 MHz ^{13}C NMR spectrum of **6g** (DMSO-d_6)....10
Figure S14. 151 MHz ^{13}C NMR spectrum of **6g** (DMSO-d_6)....10
Figure S15. 600 MHz ^{1}H NMR spectrum of **6h** (DMSO-d_6)....11
Figure S16. 151 MHz ^{13}C NMR spectrum of **6h** (DMSO-d_6)....11
Figure S17. 600 MHz ^{1}H NMR spectrum of **6i** (DMSO-d_6)....12
Figure S18. 151 MHz ^{13}C NMR spectrum of **6i** (DMSO-d_6)....12
Figure S19. 600 MHz ^{1}H NMR spectrum of **6j** (DMSO-d_6)....13
Figure S20. 151 MHz ^{13}C NMR spectrum of **6j** (DMSO-d_6)....13
Figure S21. 600 MHz ^{1}H NMR spectrum of **6k** (DMSO-d_6)....14
Figure S22. 151 MHz ^{13}C NMR spectrum of **6k** (DMSO-d_6)....14
Figure S23. 600 MHz ^{1}H NMR spectrum of **6l** (DMSO-d_6)....15
Figure S24. 151 MHz ^{13}C NMR spectrum of **6l** (DMSO-d_6)....15
Figure S25. 600 MHz ^{1}H NMR spectrum of **6n** (DMSO-d_6)....16
Figure S26. 151 MHz ^{13}C NMR spectrum of **6n** (DMSO-d_6)....16
Figure S27. 600 MHz ^{1}H NMR spectrum of **6p** (DMSO-d_6)....17
Figure S28. 151 MHz ^{13}C NMR spectrum of **6p** (DMSO-d_6)....17
Figure S29. 600 MHz ^{1}H NMR spectrum of **6q** (DMSO-d_6)....18
Figure S30. 151 MHz ^{13}C NMR spectrum of **6q** (DMSO-d_6....18
Figure S31. 600 MHz ^{1}H NMR spectrum of **6r** (DMSO-d_6)....19

Figure S32. 151 MHz ^{13}C NMR spectrum of **6r** (DMSO-d_6) 19
Figure S33. 600 MHz ^{1}H NMR spectrum of **7b** (DMSO-d_6) 20
Figure S34. 151 MHz ^{13}C NMR spectrum of **7b** (DMSO-d_6) 20
Figure S35. 600 MHz ^{1}H NMR spectrum of **7c** (MeOH-d_4) 21
Figure S36. 151 MHz ^{13}C NMR spectrum of **7c** (MeOH-d_4) 21
Figure S37. 600 MHz ^{1}H NMR spectrum of **7d** (MeOH-d_4) 22
Figure S38. 151 MHz ^{13}C NMR spectrum of **7d** (MeOH-d_4) 22
Figure S39. 600 MHz ^{1}H NMR spectrum of **7g** (DMSO-d_6) 23
Figure S40. 151 MHz ^{13}C NMR spectrum of **7g** (DMSO-d_6) 23
Figure S41. 600 MHz ^{1}H NMR spectrum of **7h** (MeOH-d_4) 24
Figure S42. 151 MHz ^{13}C NMR spectrum of **7h** (MeOH-d_4) 24
Figure S43. 600 MHz ^{1}H NMR spectrum of **7i** (DMSO-d_6) 25
Figure S44. 151 MHz ^{13}C NMR spectrum of **7i** (DMSO-d_6) 25
Figure S45. 600 MHz ^{1}H NMR spectrum of **7j** (DMSO-d_6) 26
Figure S46. 151 MHz ^{13}C NMR spectrum of **7j** (DMSO-d_6) 26
Figure S47. 600 MHz ^{1}H NMR spectrum of **7k** (DMSO-d_6) 27
Figure S48. 151 MHz ^{13}C NMR spectrum of **7k** (DMSO-d_6) 27
Figure S49. 600 MHz ^{1}H NMR spectrum of **7l** (DMSO-d_6) 28
Figure S50. 151 MHz ^{13}C NMR spectrum of **7l** (DMSO-d_6) 28
Figure S51. 600 MHz ^{1}H NMR spectrum of **7m** (DMSO-d_6) 29
Figure S52. 151 MHz ^{13}C NMR spectrum of **7m** (MeOH-d_4) 29
Figure S53. 600 MHz ^{1}H NMR spectrum of **7n** (MeOH-d_4) 30
Figure S54. 151 MHz ^{13}C NMR spectrum of **7n** (MeOH-d_4) 30
Figure S55. Agarose gel electrophoresis result showing inhibition of QCA **7i** and **7m** against *Mtb* DNA gyrase; (-) negative control without gyrase; (+) positive control with gyrase......32

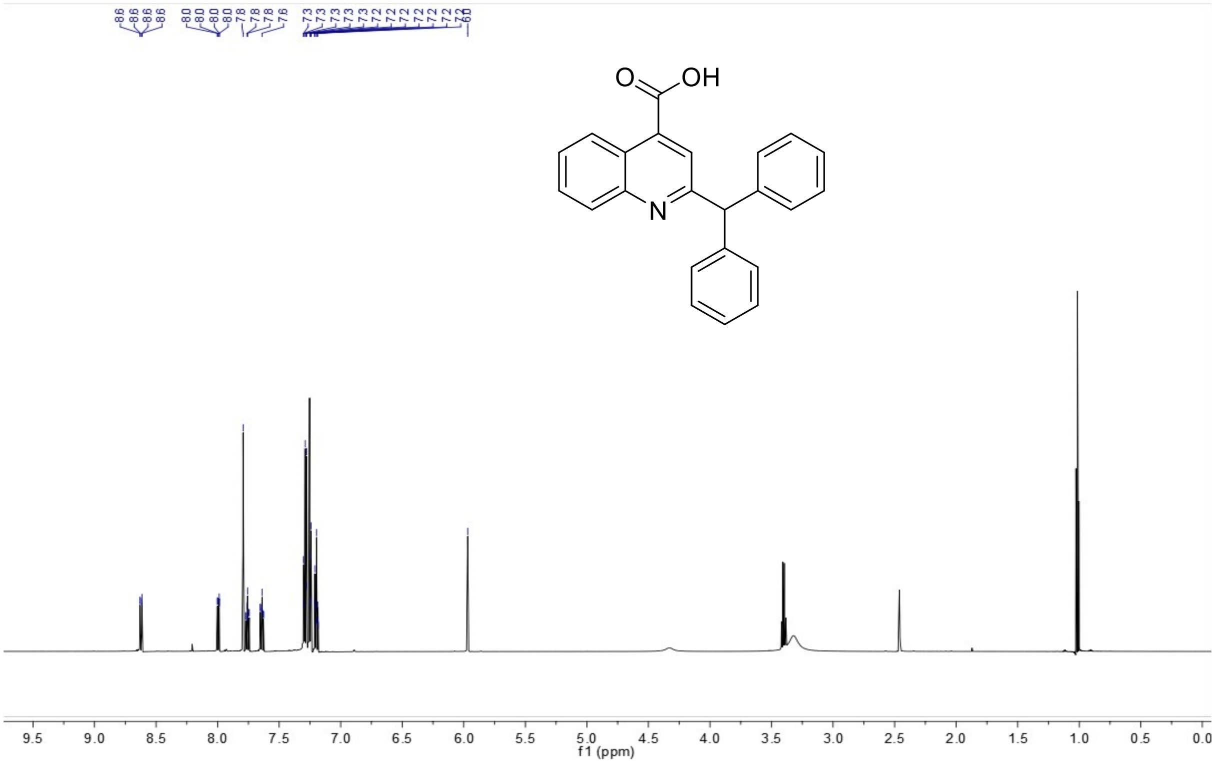

Figure S1. 600 MHz ^{1}H NMR spectrum of **1d** (DMSO-d_6)

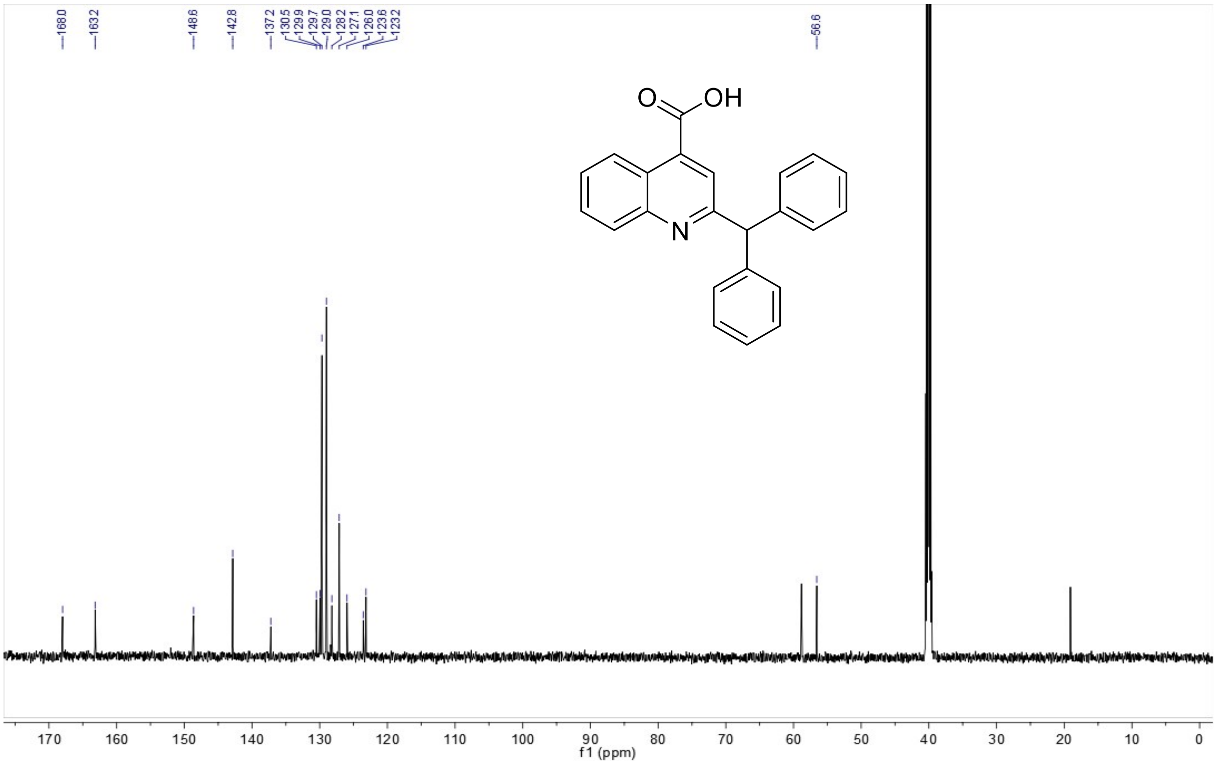

Figure S2. 151 MHz ^{13}C NMR spectrum of **1d** (DMSO-d_6)

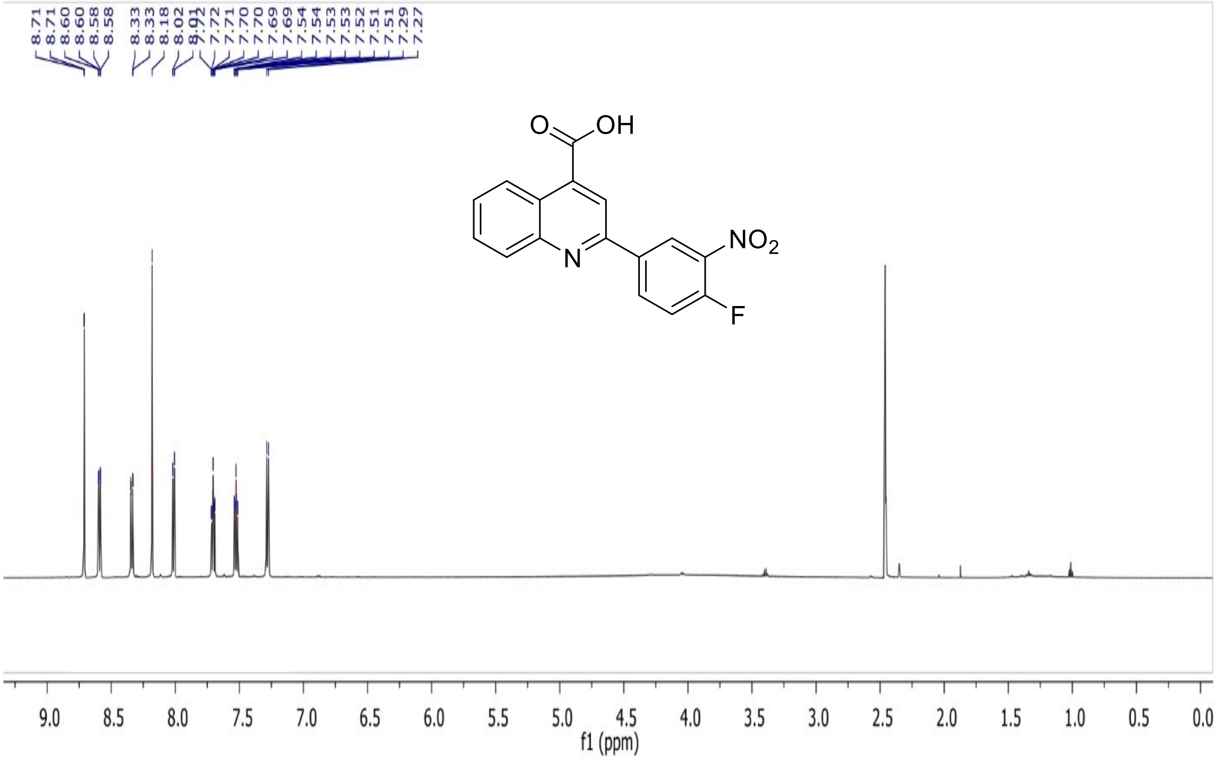

Figure S3. 600 MHz ^{13}H NMR spectrum of **1o** (DMSO-d_6)

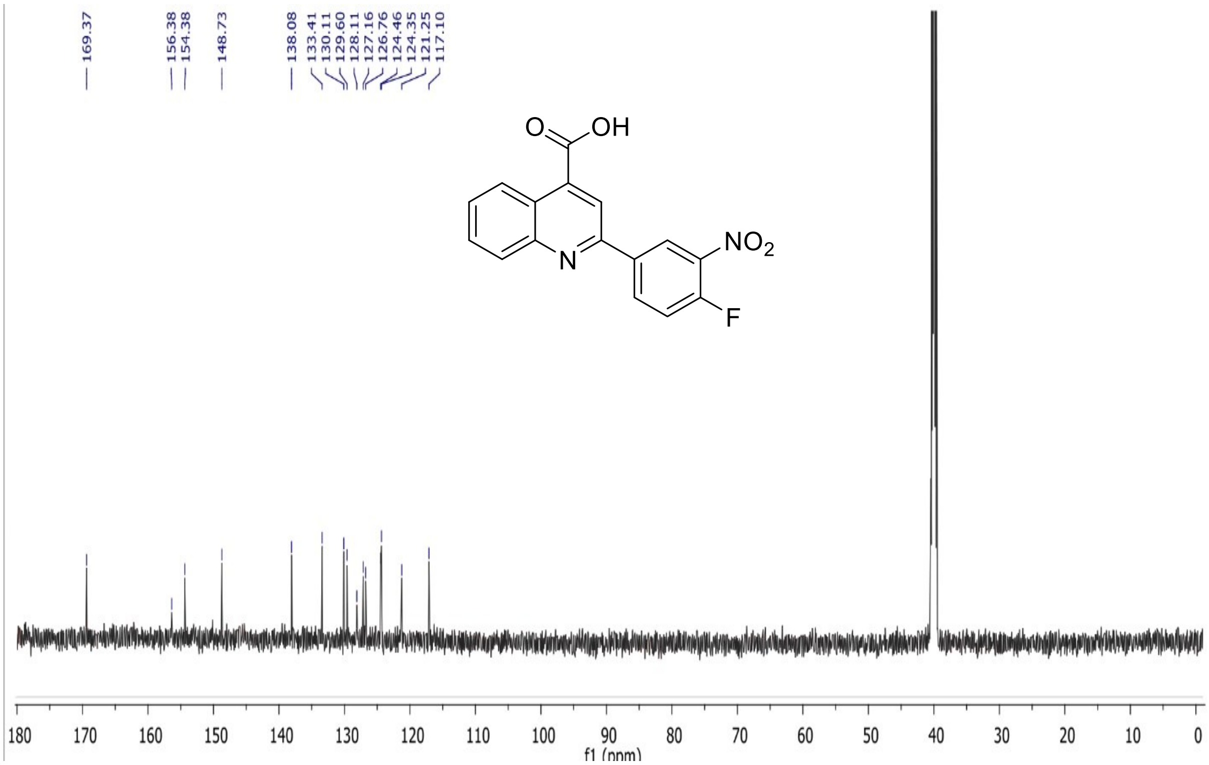

Figure S4. 151 MHz ^{13}C NMR spectrum of **1o** (DMSO-d_6)

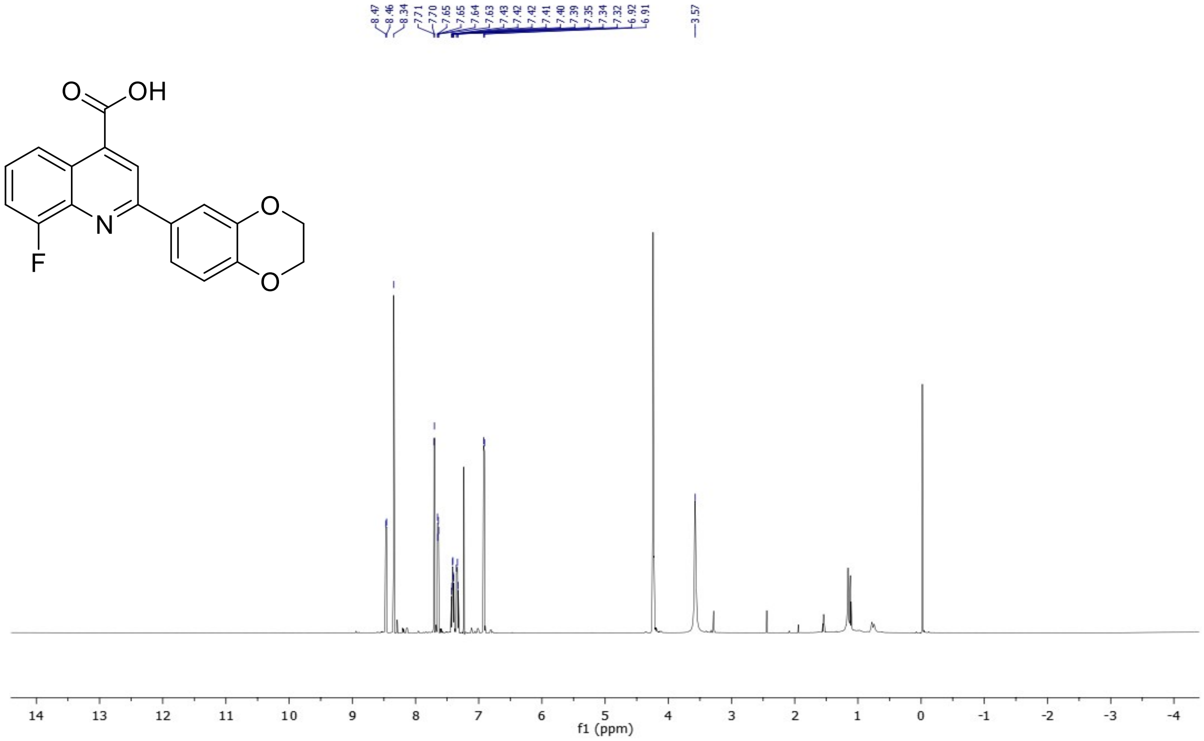

Figure S5. 600 MHz ^{1}H NMR spectrum of **1p** (MeOH-d_4)

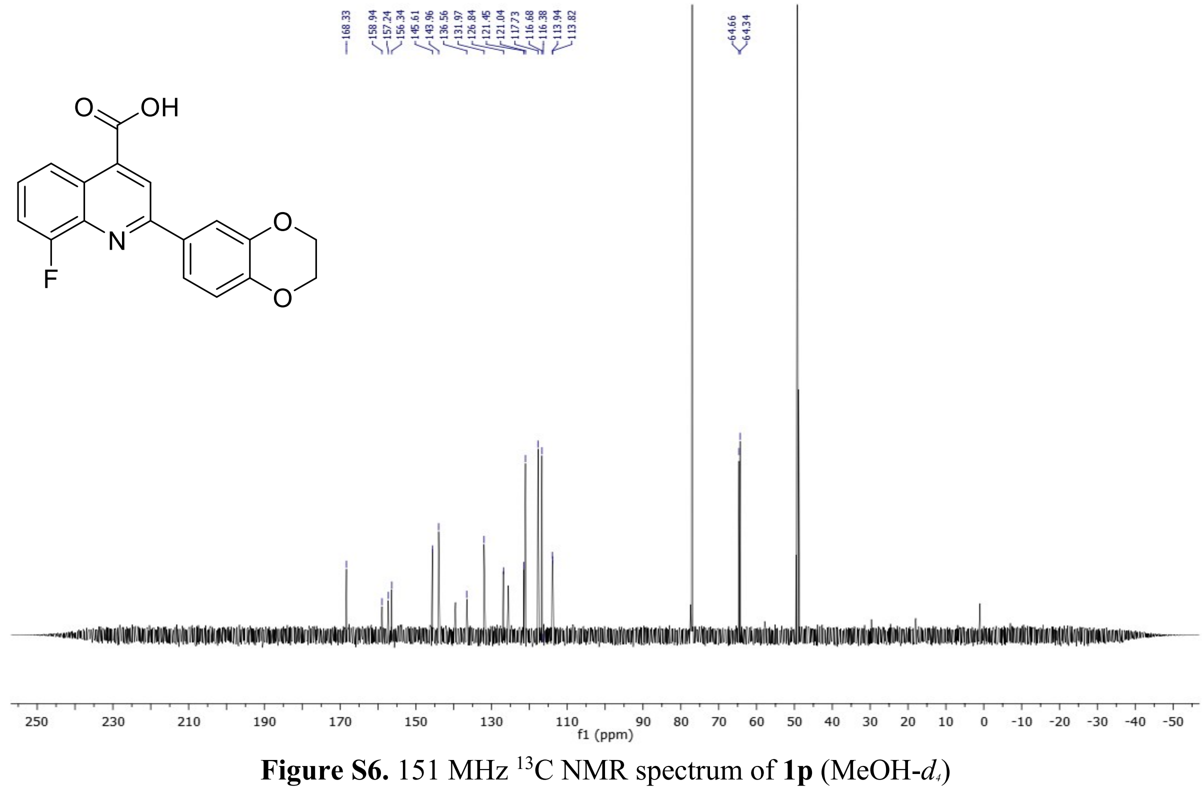

Figure S6. 151 MHz ^{13}C NMR spectrum of **1p** (MeOH-d_4)

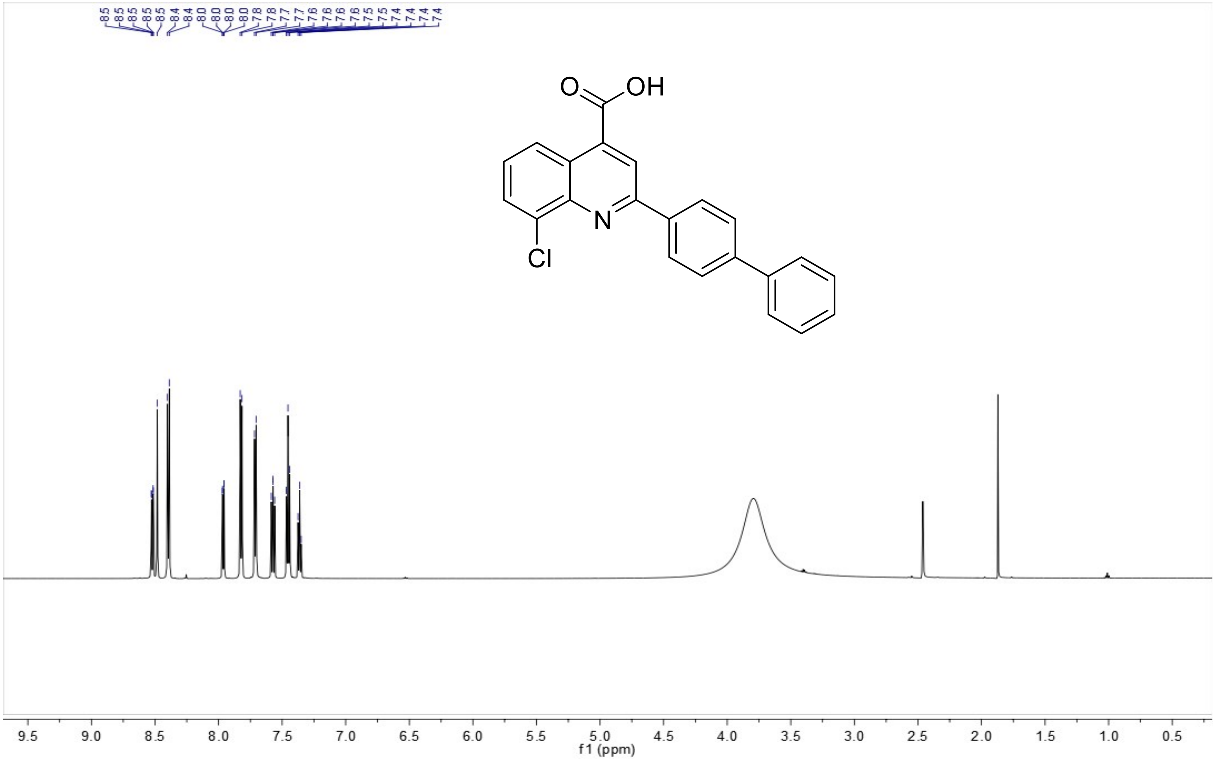

Figure S7. 600 MHz ^{1}H NMR spectrum of **6b** (DMSO-d_6)

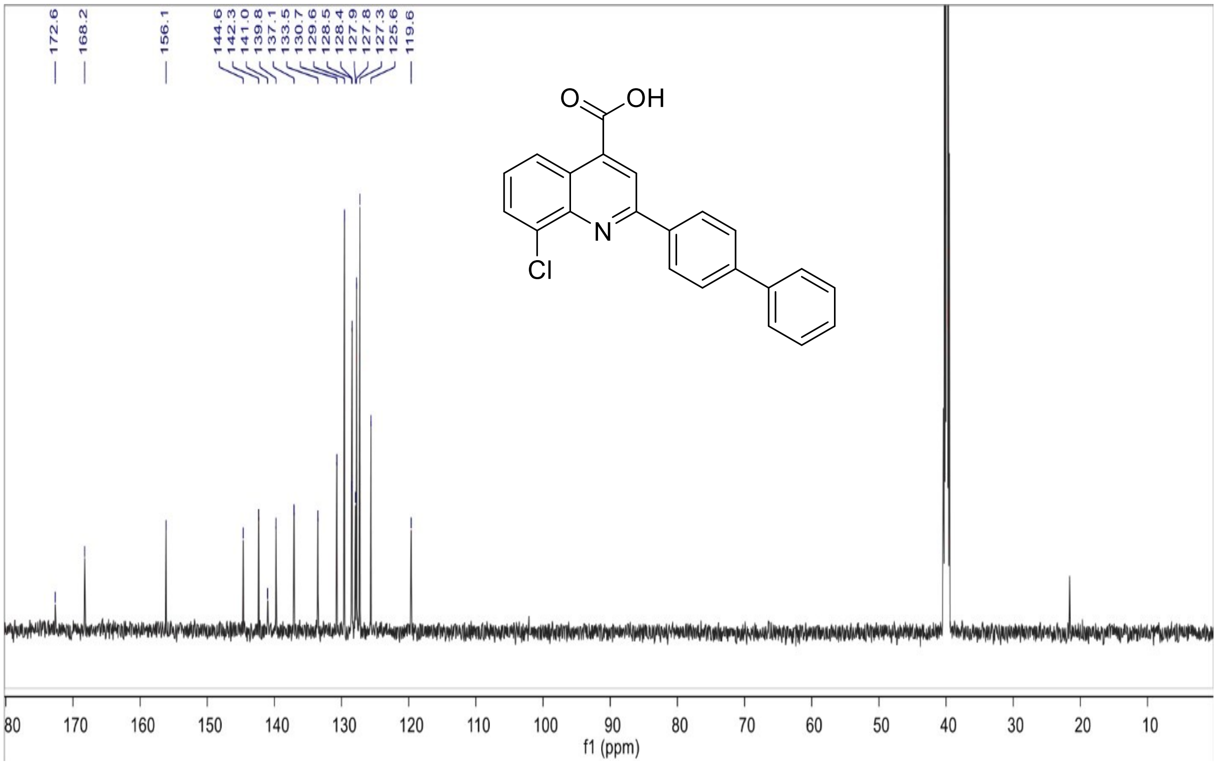

Figure S8. 151 MHz ^{13}C NMR spectrum of **6b** (DMSO-d_6)

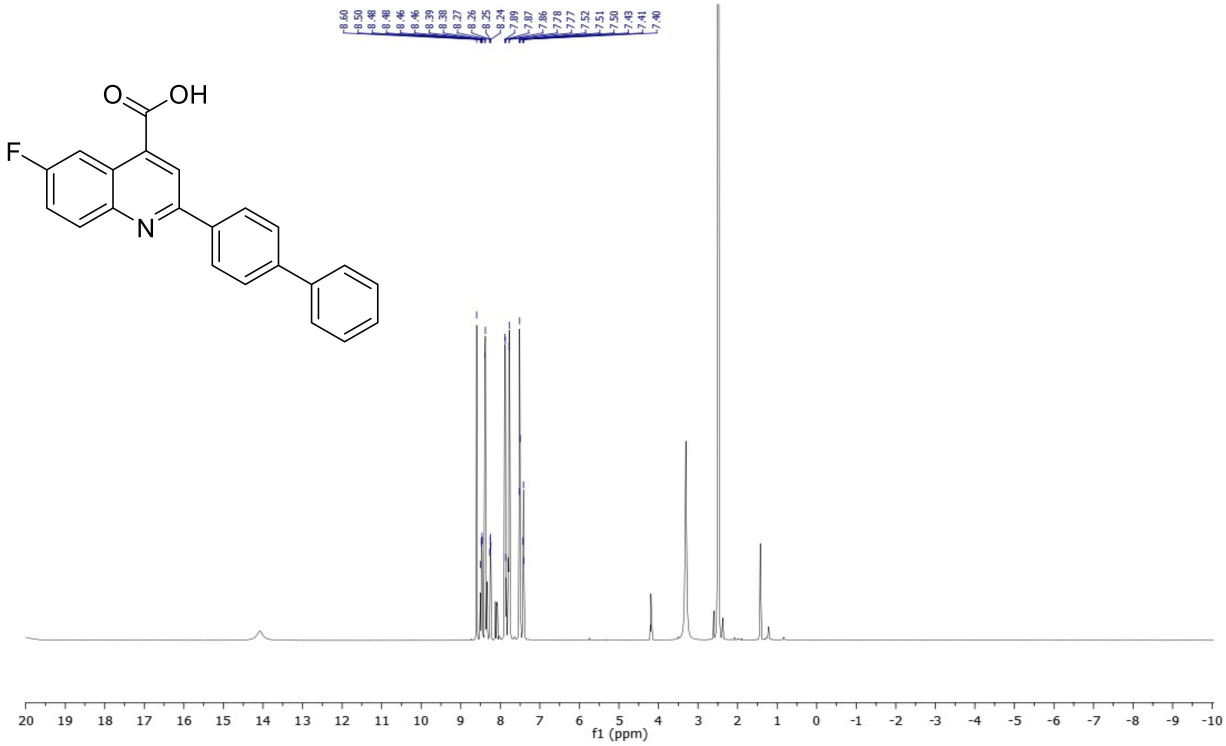

Figure S9. 600 MHz ^{13}C NMR spectrum of **6c** (DMSO-d_6)

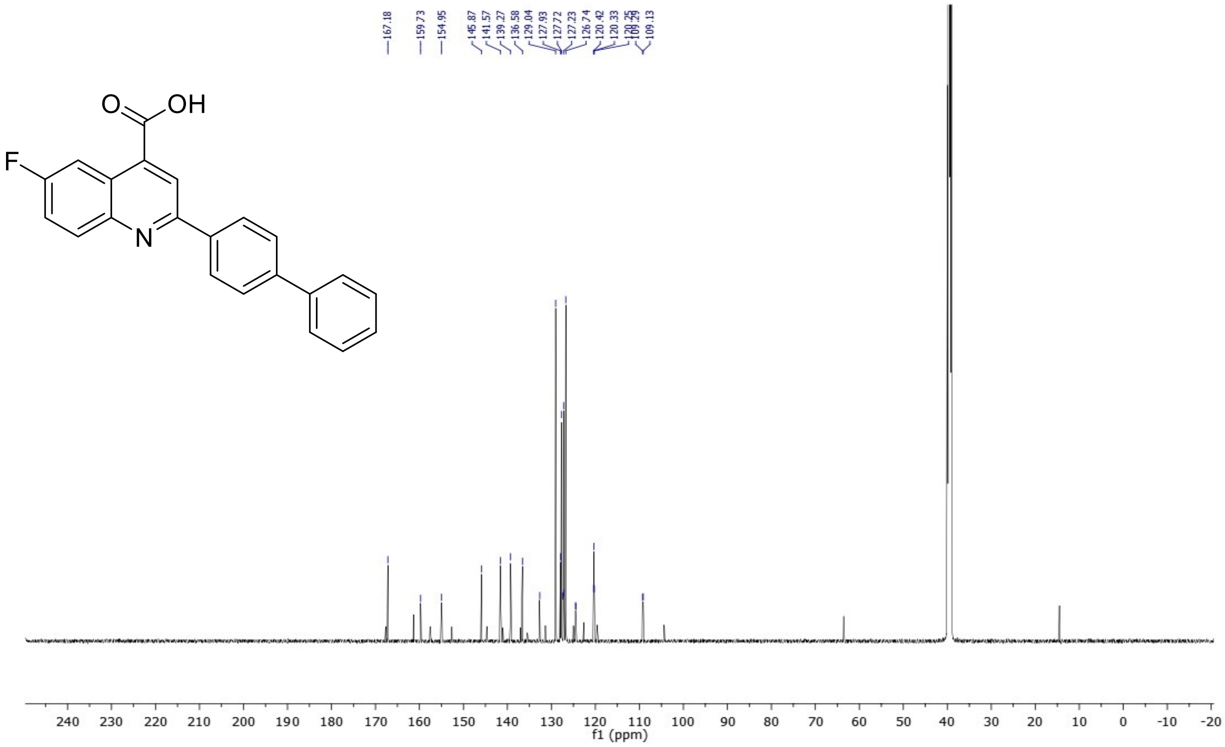

Figure S10. 151 MHz ^{13}C NMR spectrum of **6c** (DMSO-d_6)

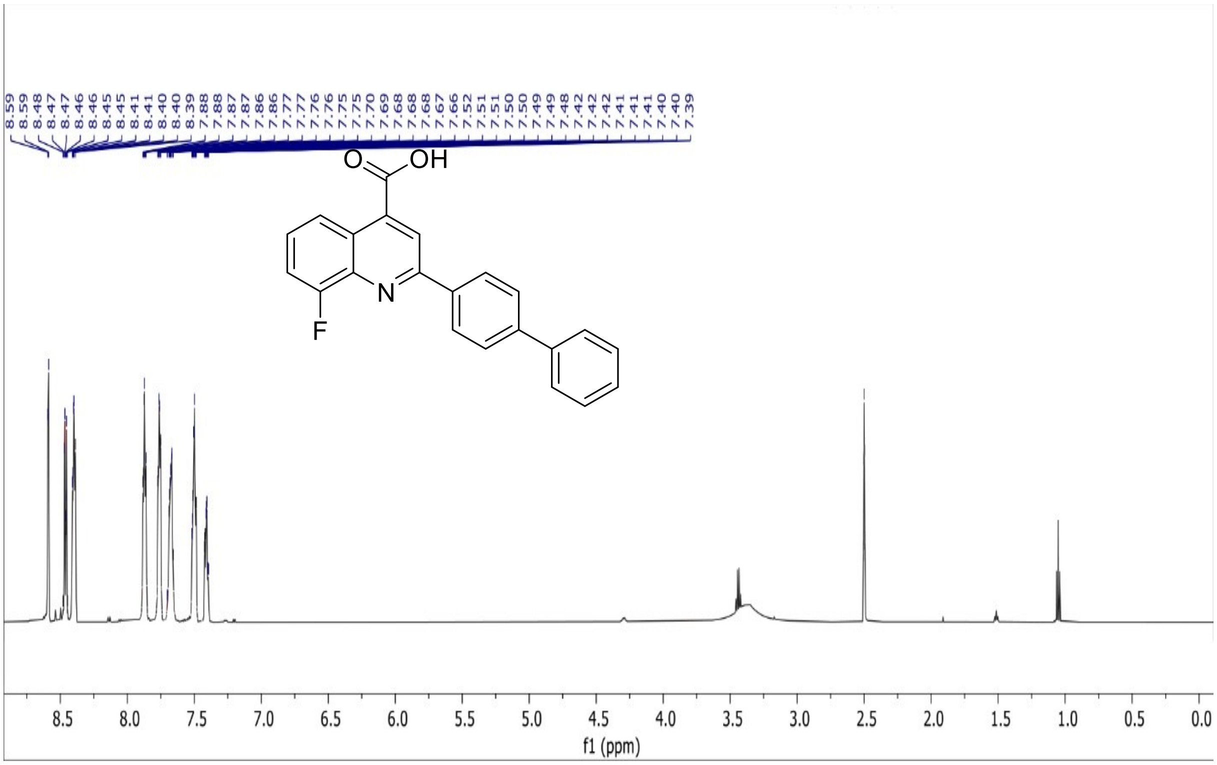

Figure S11. 600 MHz ^{1}H NMR spectrum of **6d** (DMSO-d_6)

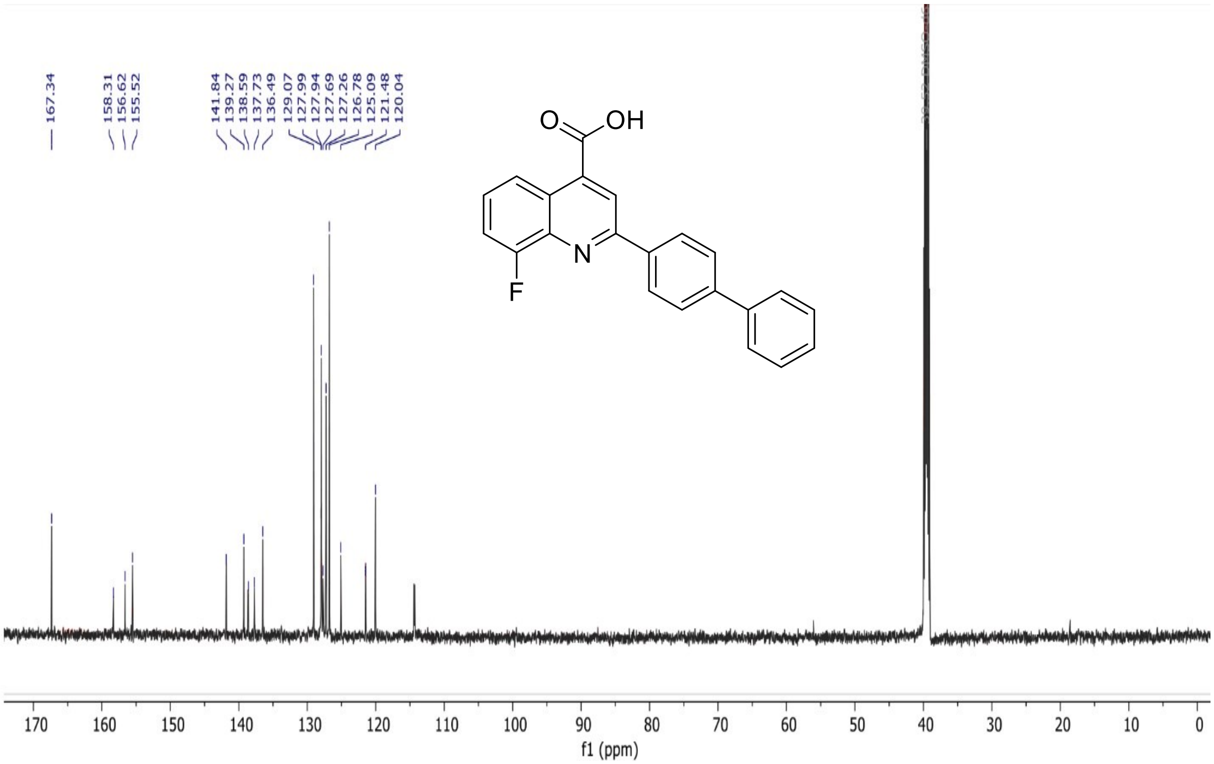

Figure S12. 151 MHz ^{13}C NMR spectrum of **6d** (DMSO-d_6)

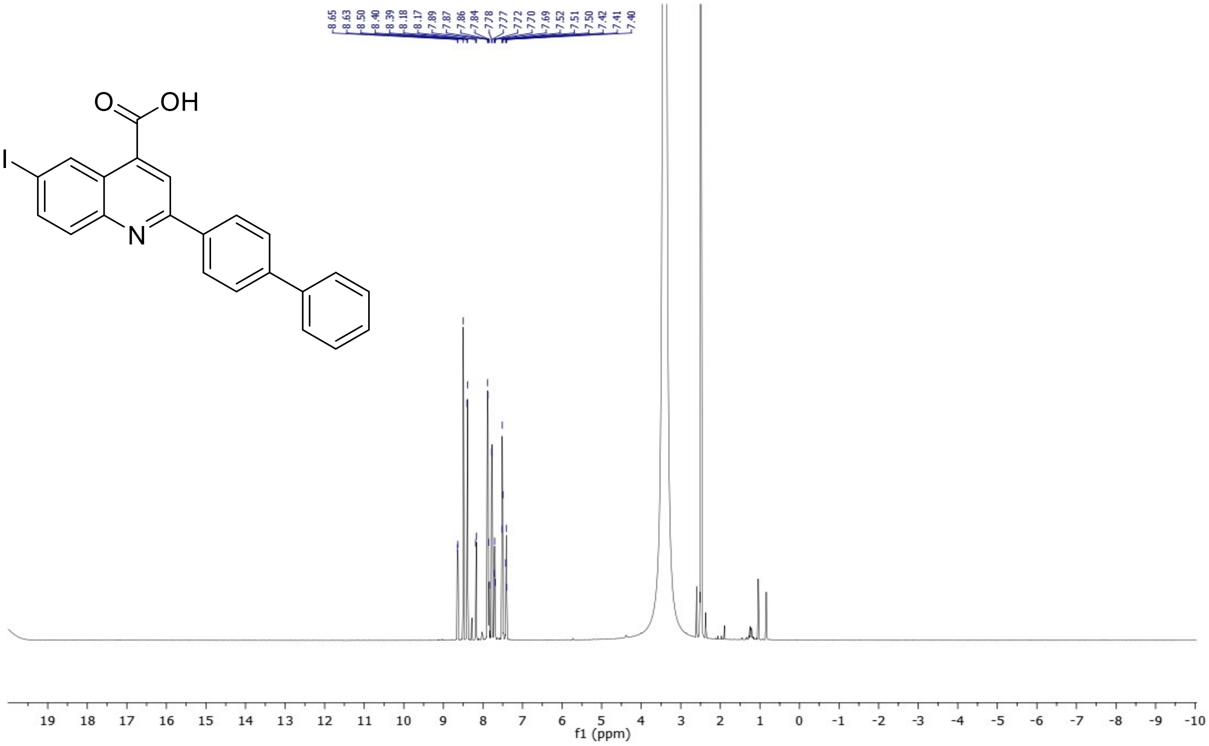

Figure S13. 600 MHz ^{13}C NMR spectrum of **6g** (DMSO-d_6)

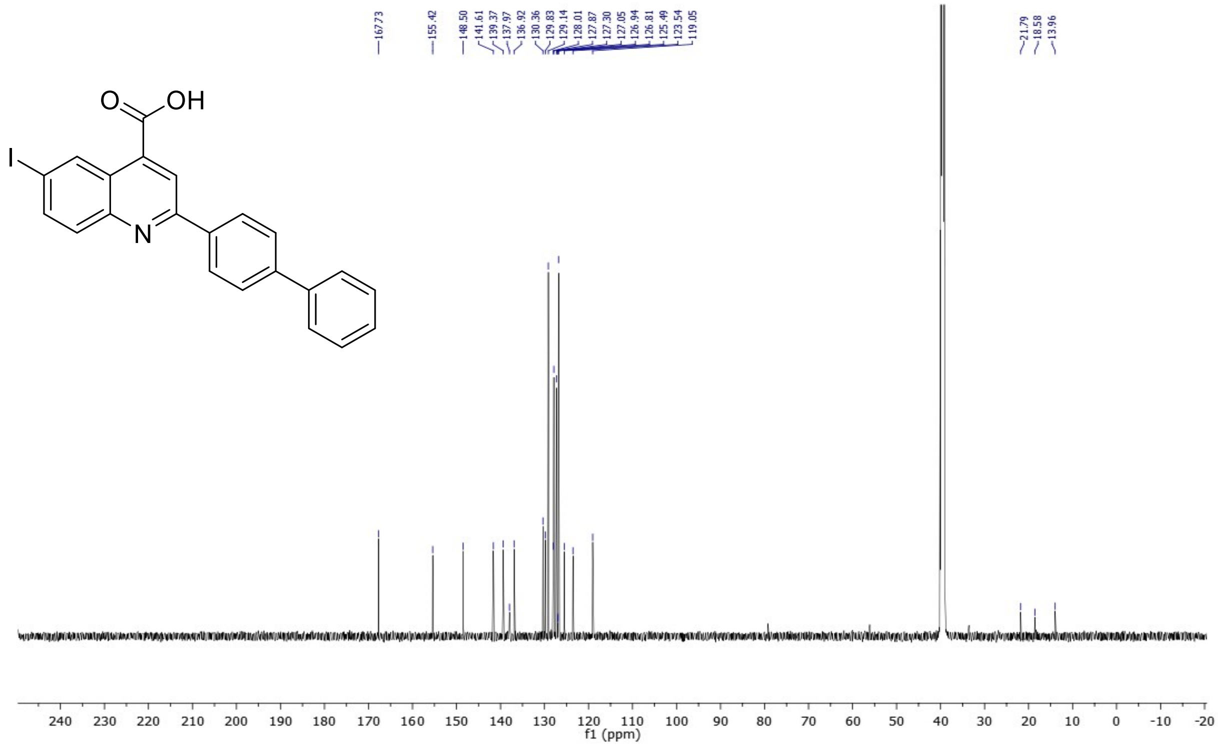

Figure S14. 151 MHz ^{13}C NMR spectrum of **6g** (DMSO-d_6)

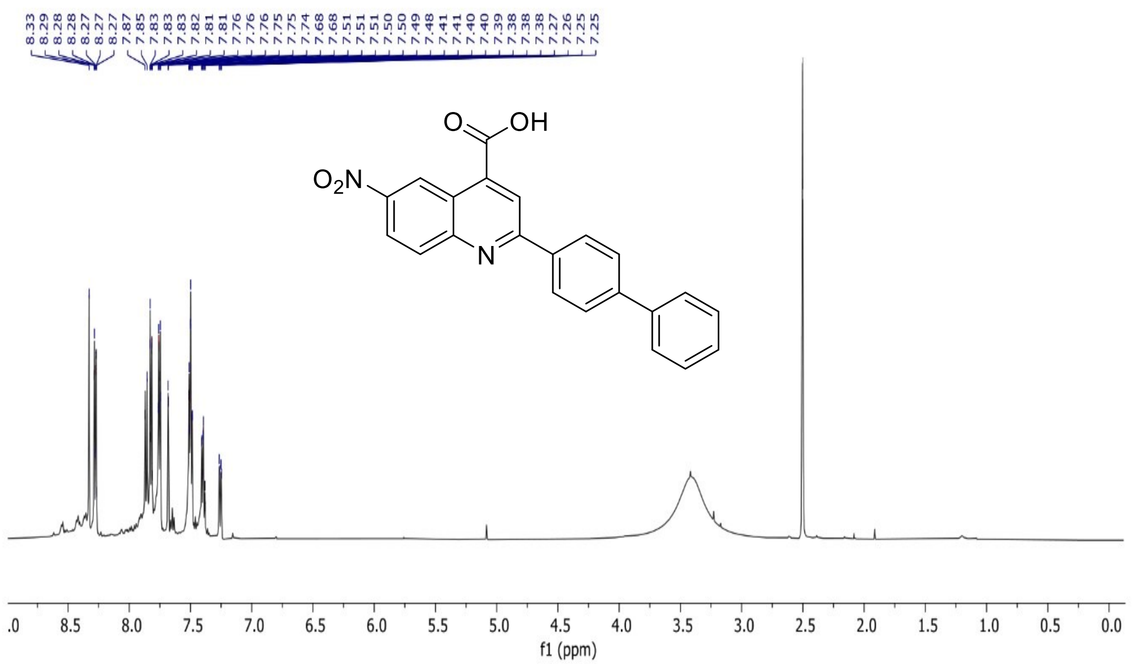

Figure S15. 600 MHz ^{1}H NMR spectrum of **6h** (DMSO-d_6)

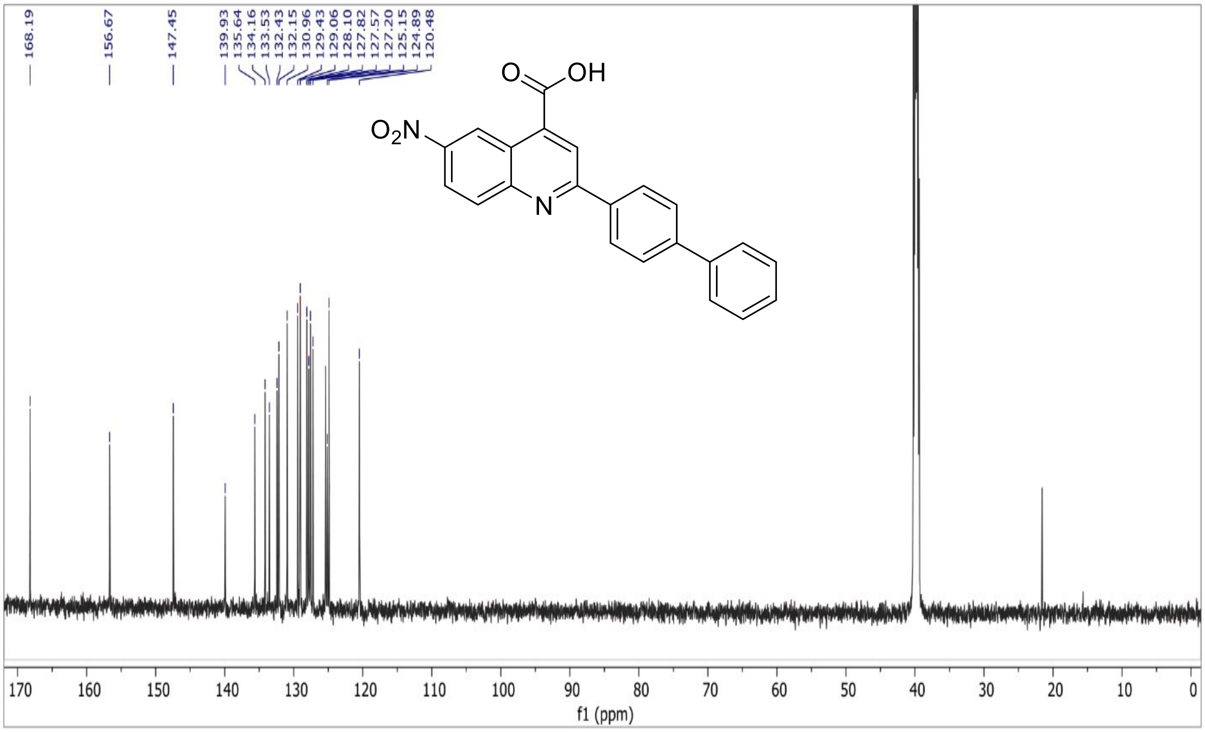

Figure S16. 151 MHz ^{13}C NMR spectrum of **6h** (DMSO-d_6)

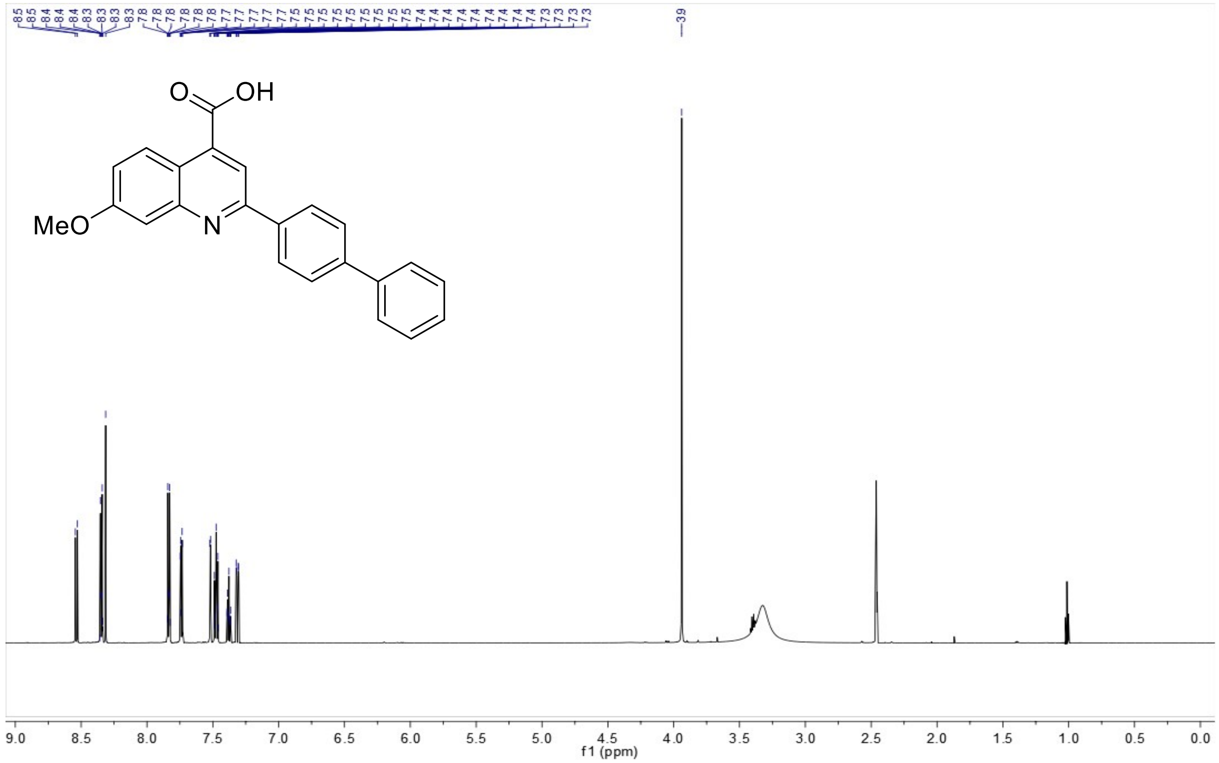

Figure S17. 600 MHz ^{1}H NMR spectrum of **6i** (DMSO-d_6)

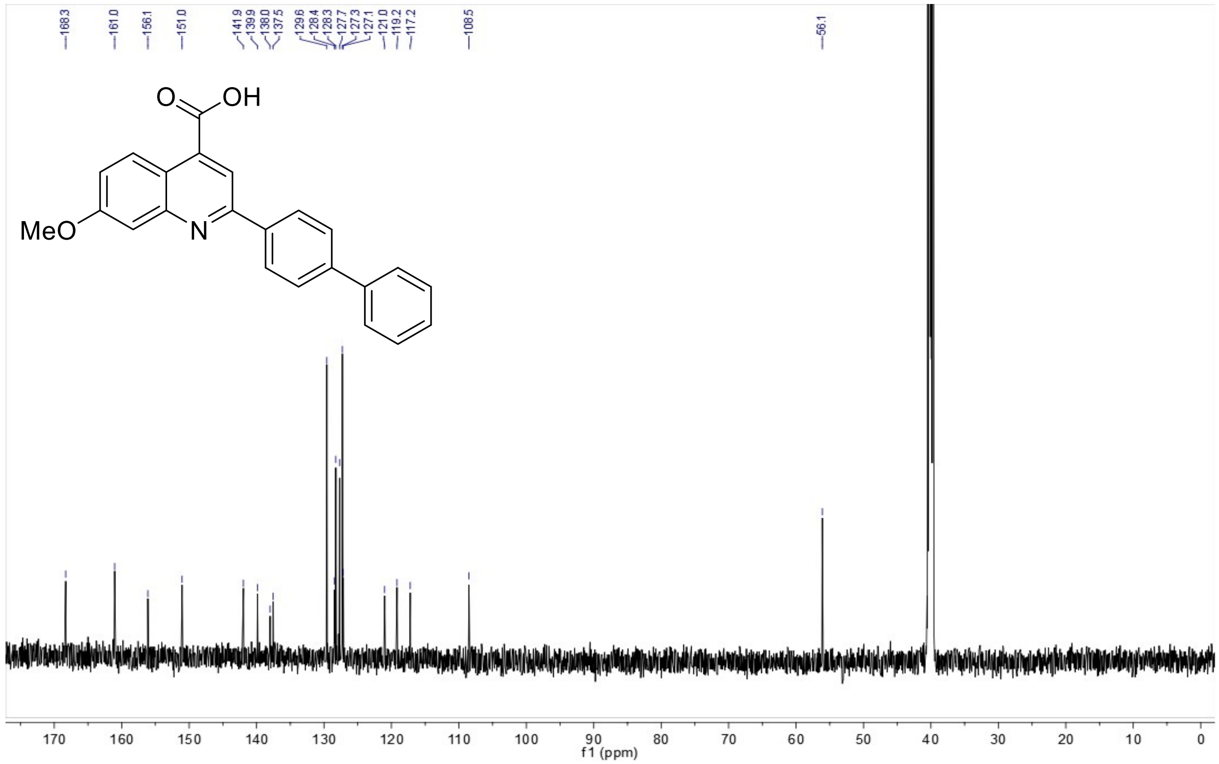

Figure S18. 151 MHz ^{13}C NMR spectrum of **6i** (DMSO-d_6)

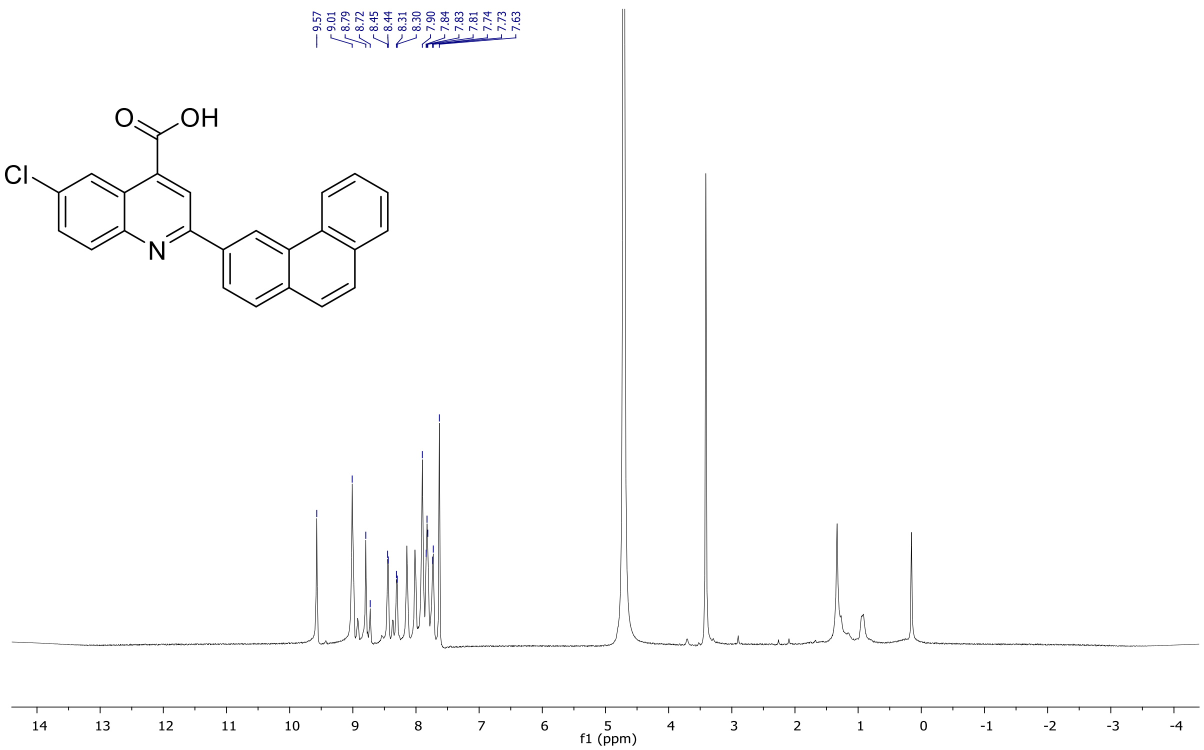

Figure S19. 600 MHz ^{1}H NMR spectrum of **6j** (DMSO-d_6)

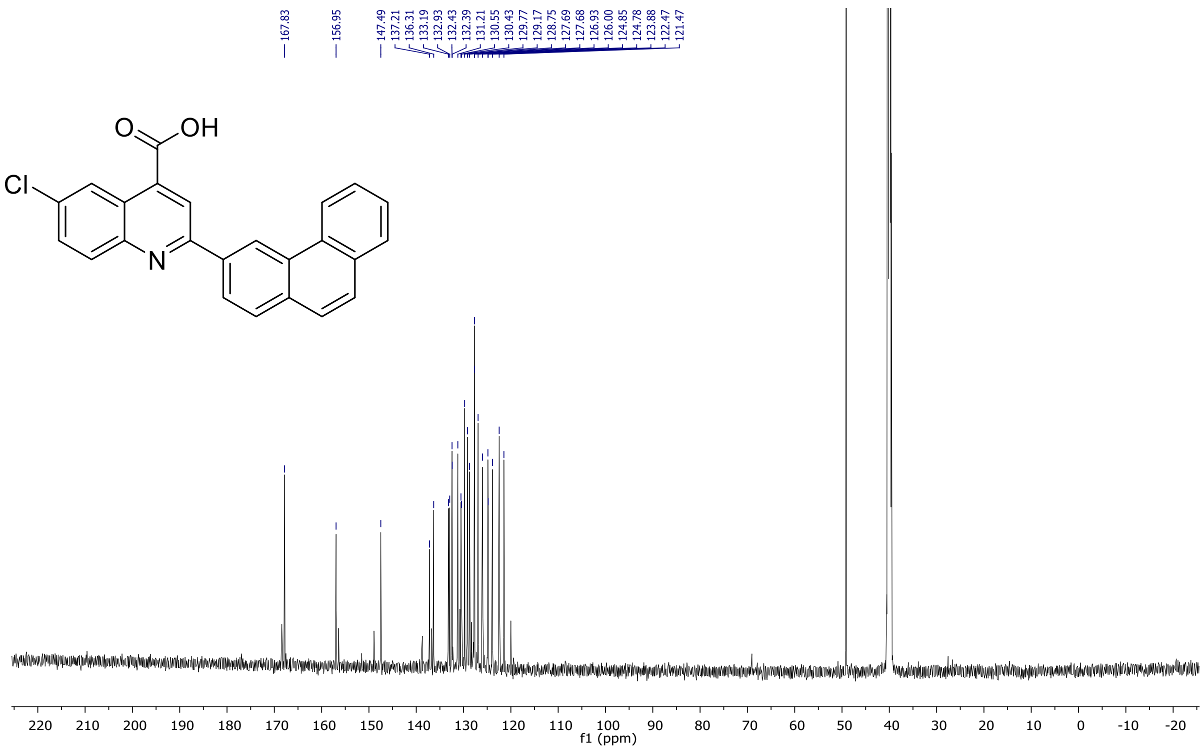

Figure S20. 151 MHz ^{13}C NMR spectrum of **6j** (DMSO-d_6)

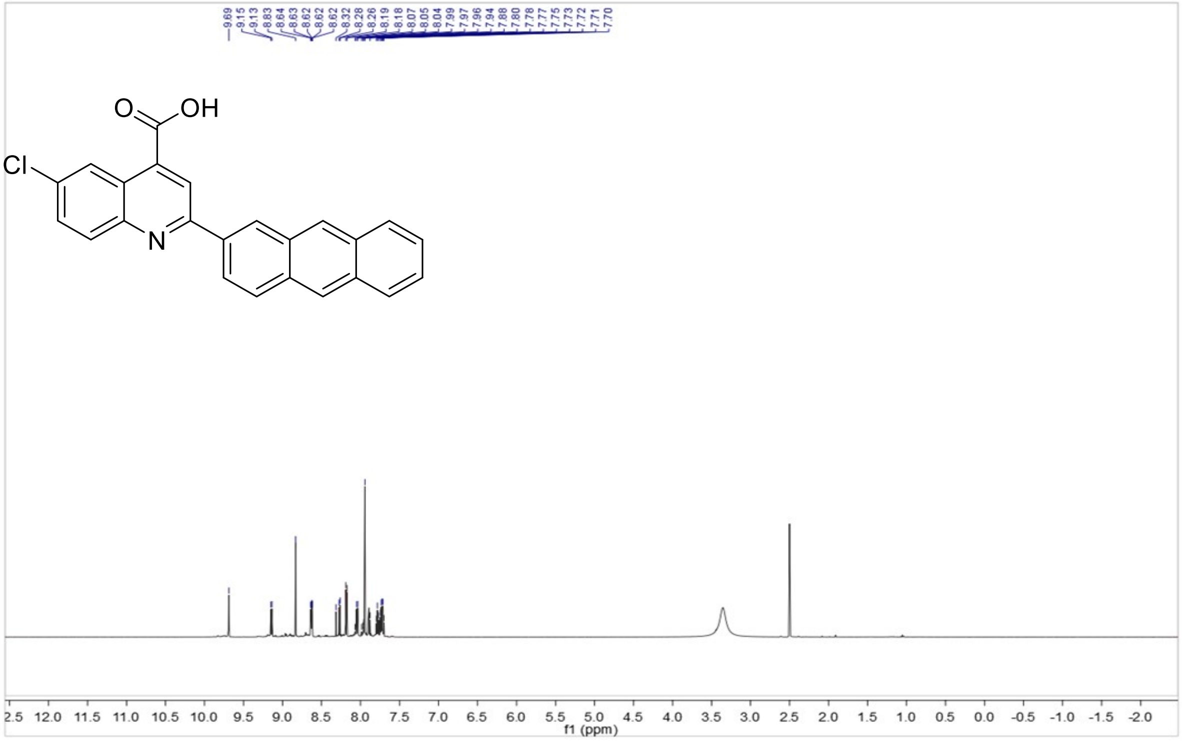

Figure S21. 600 MHz ^{1}H NMR spectrum of **6k** (DMSO-d_6)

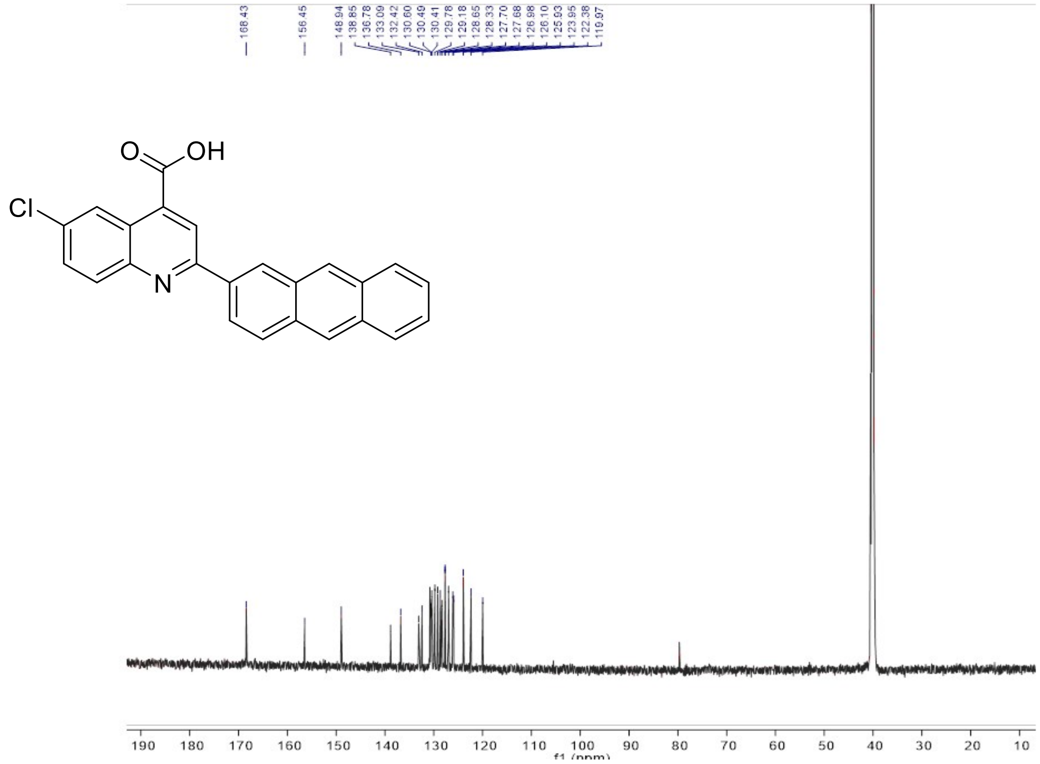

Figure S22. 151 MHz ^{13}C NMR spectrum of **6k** (DMSO-d_6)

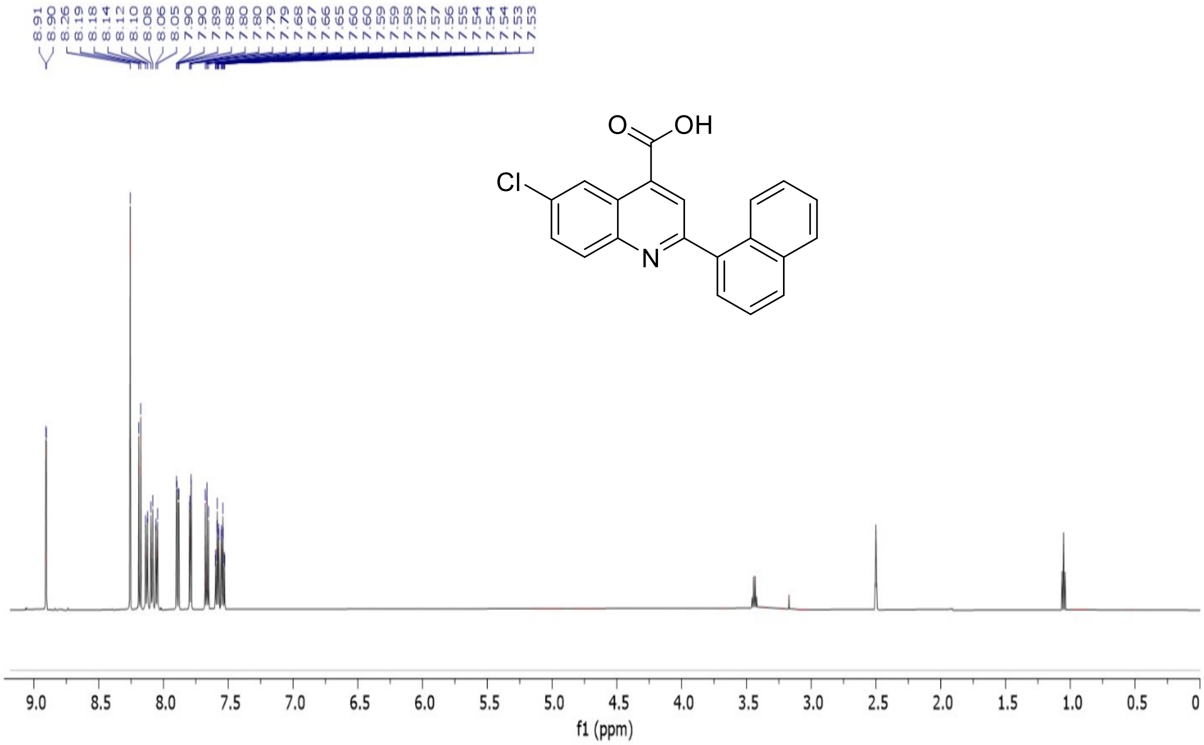

Figure S23. 600 MHz ^{1}H NMR spectrum of **6l** (DMSO-d_6)

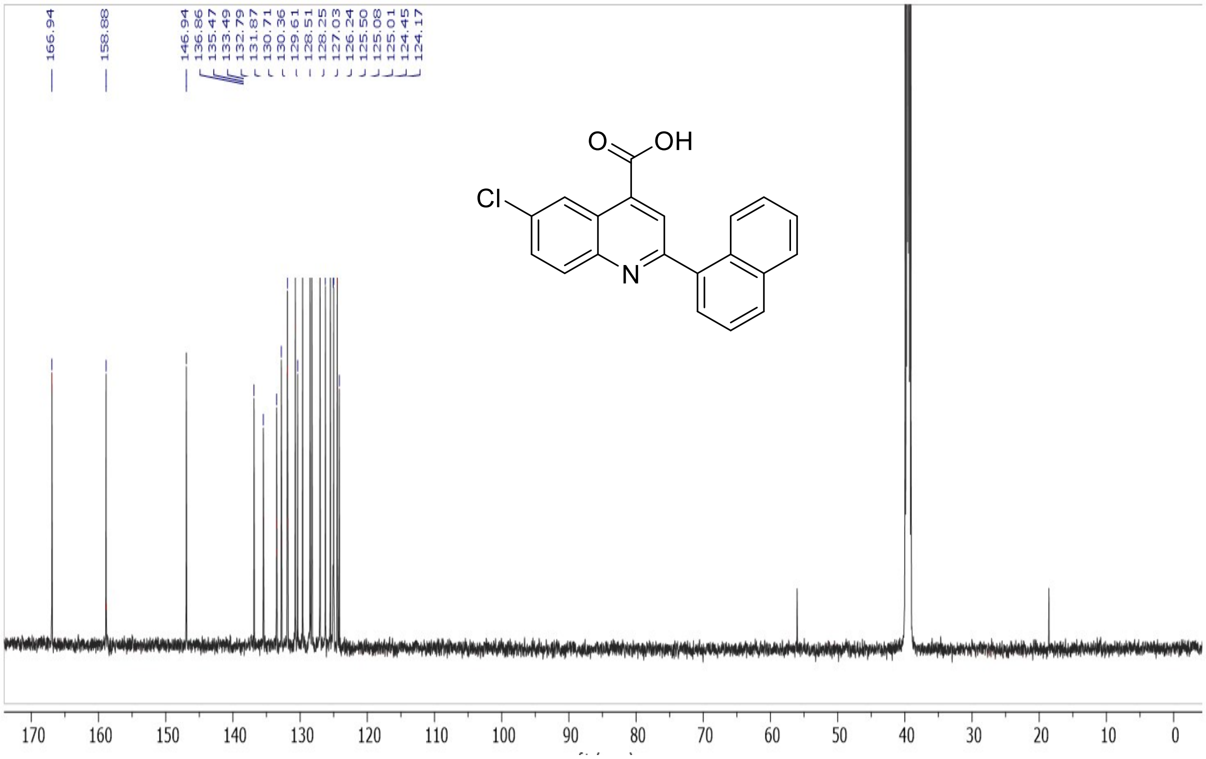

Figure S24. 151 MHz ^{13}C NMR spectrum of **6l** (DMSO-d_6)

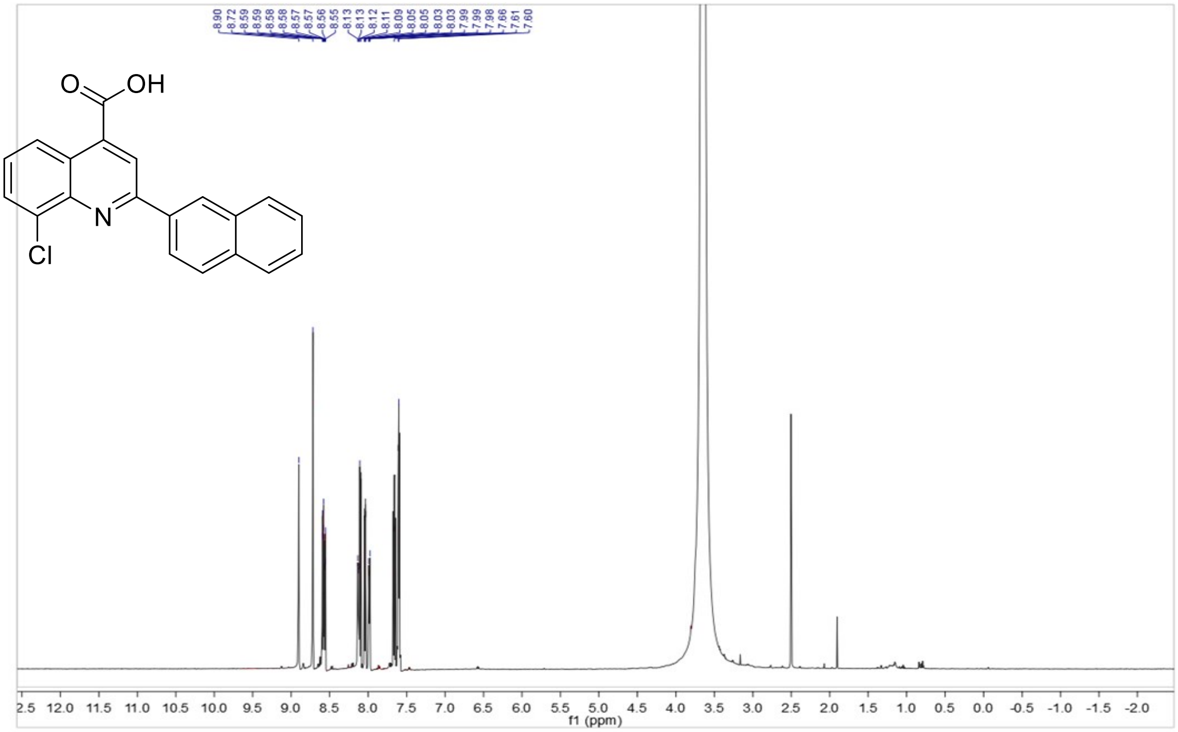

Figure S25. 600 MHz ^{1}H NMR spectrum of **6n** (DMSO-d_6)

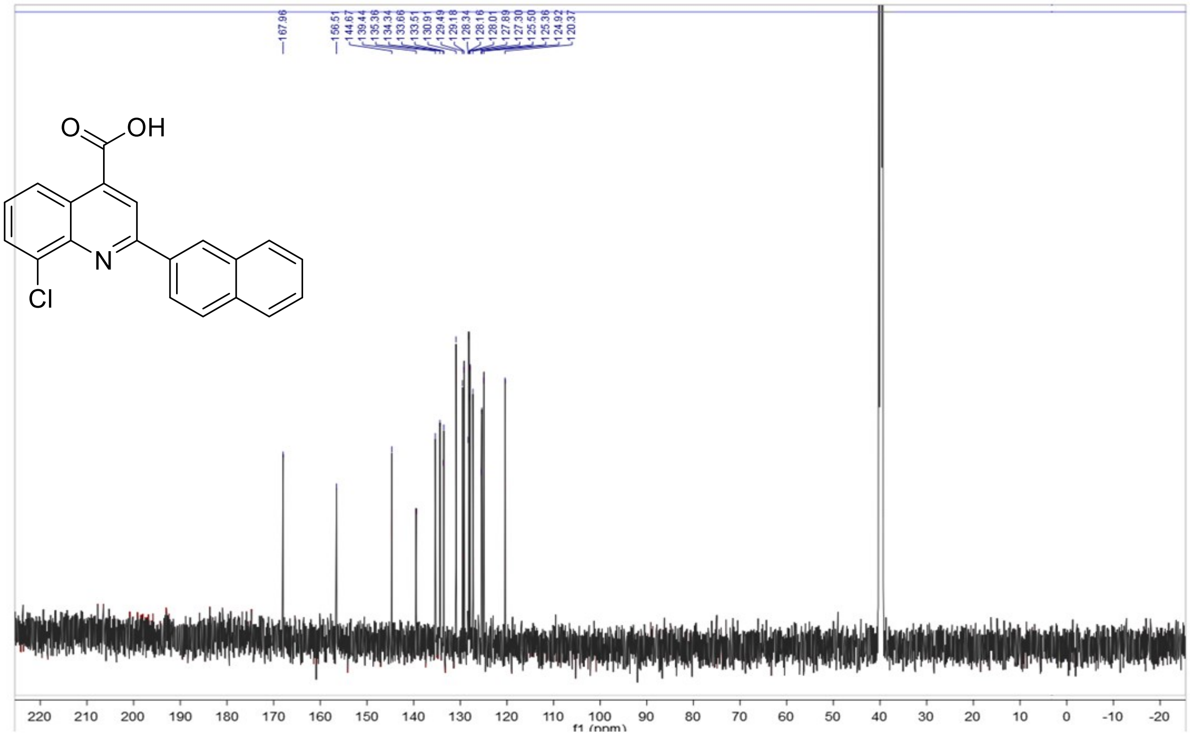

Figure S26. 151 MHz ^{13}C NMR spectrum of **6n** (DMSO-d_6)

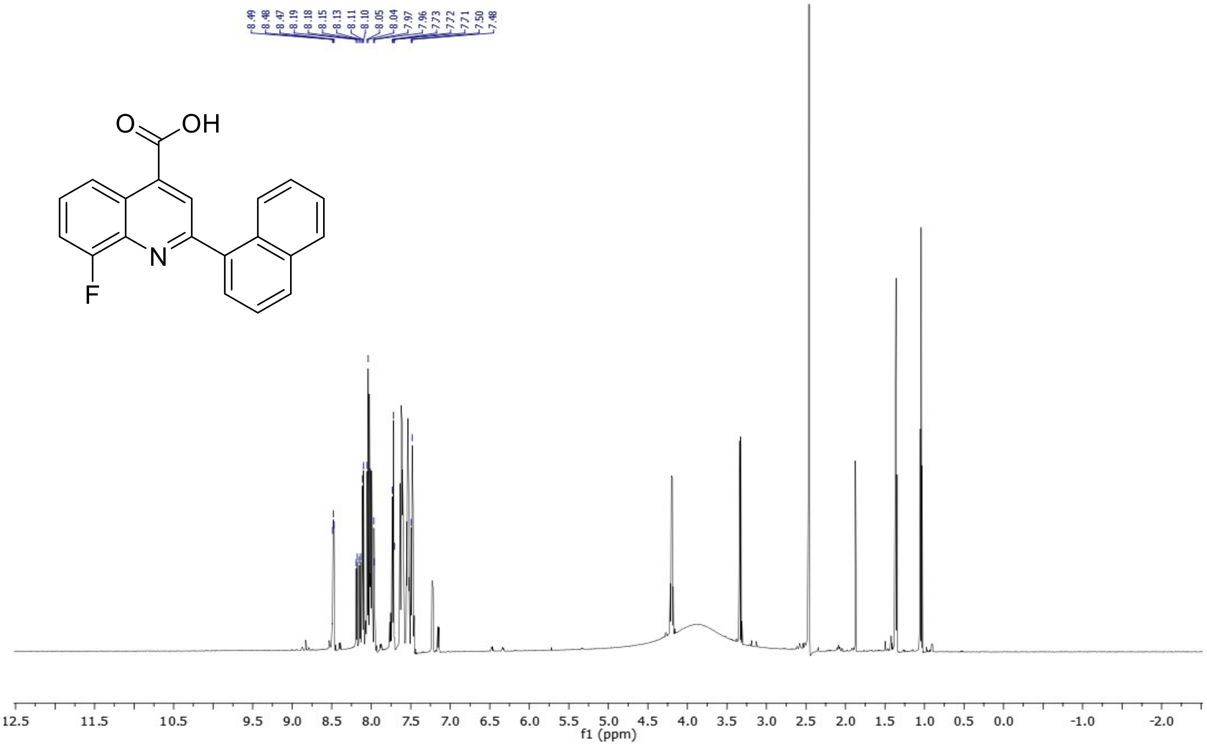

Figure S27. 600 MHz ^{1}H NMR spectrum of **6p** (DMSO-d_6)

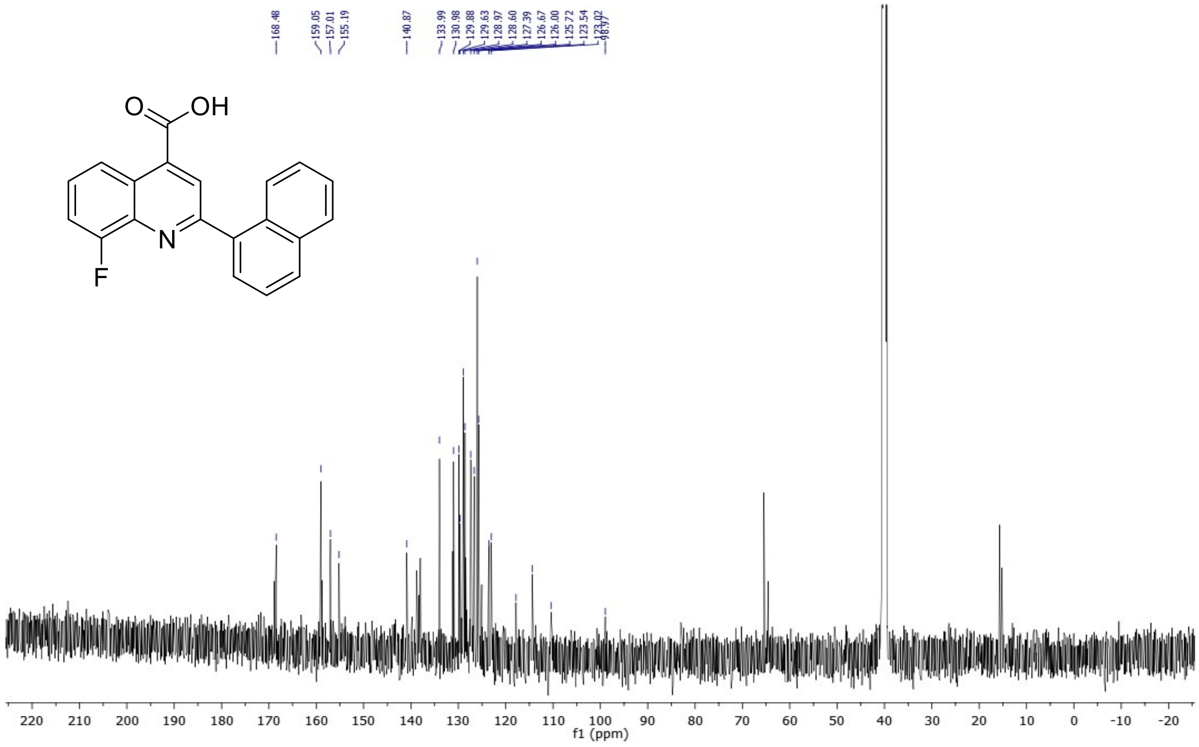

Figure S28. 151 MHz ^{13}C NMR spectrum of **6p** (DMSO-d_6)

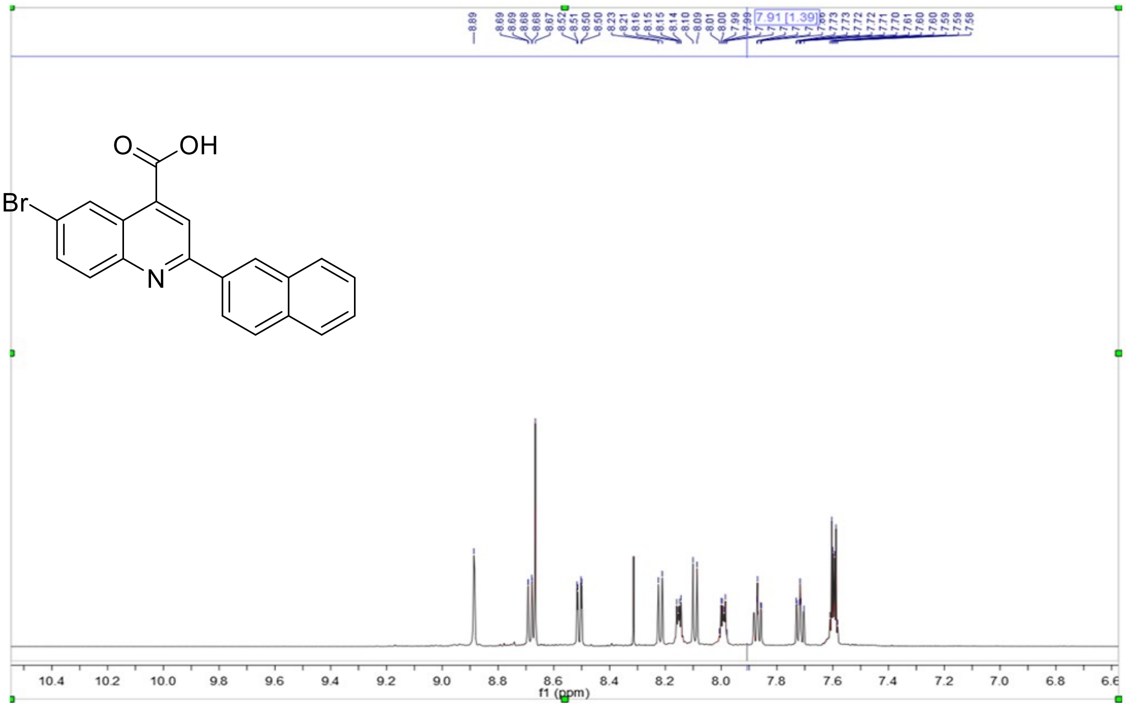

Figure S29. 600 MHz ^{1}H NMR spectrum of **6q** (DMSO-d_6)

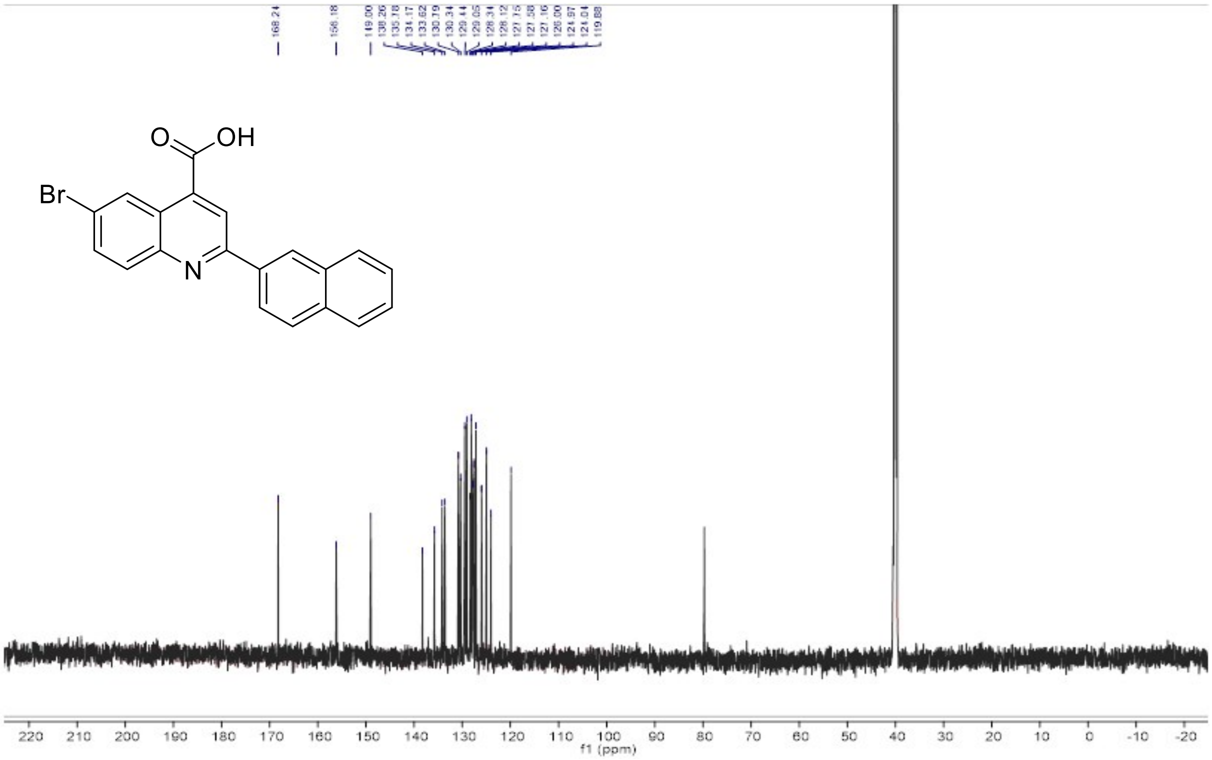

Figure S30. 151 MHz ^{13}C NMR spectrum of **6q** (DMSO-d_6

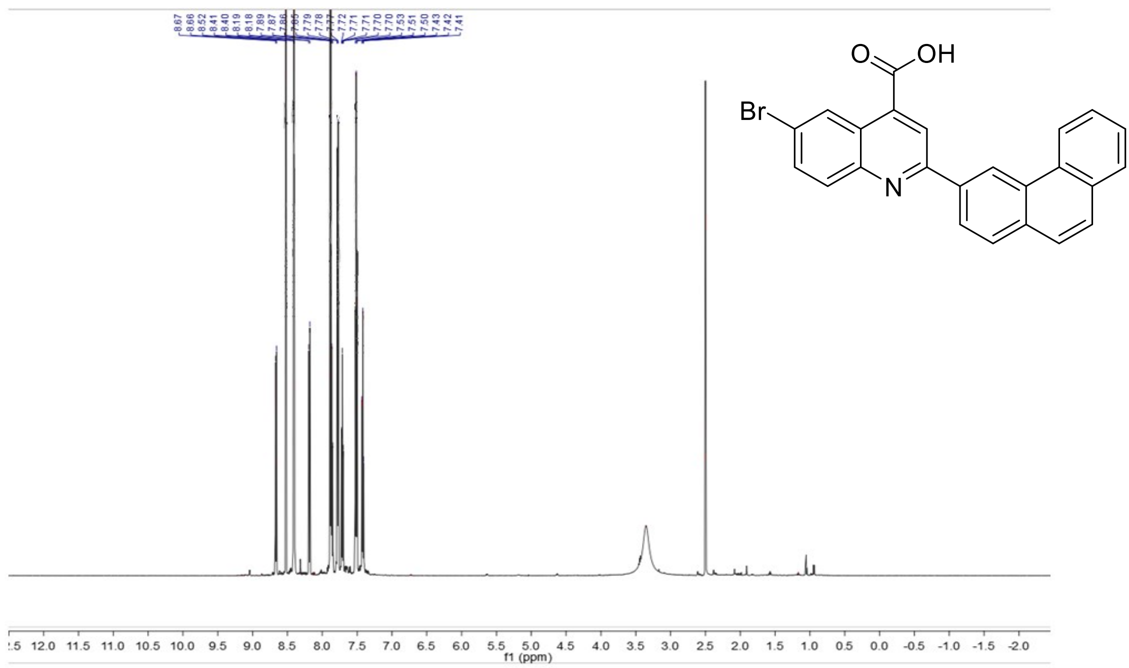

Figure S31. 600 MHz ^{1}H NMR spectrum of **6r** (DMSO-d_6)

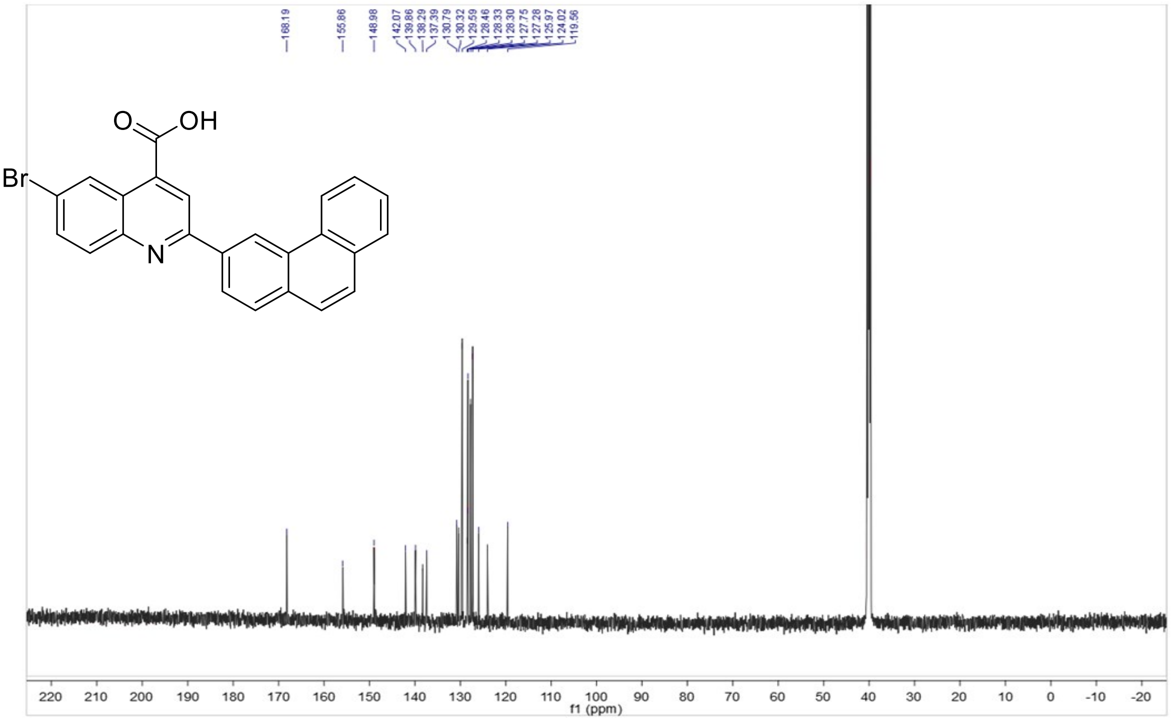

Figure S32. 151 MHz ^{13}C NMR spectrum of **6r** (DMSO-d_6)

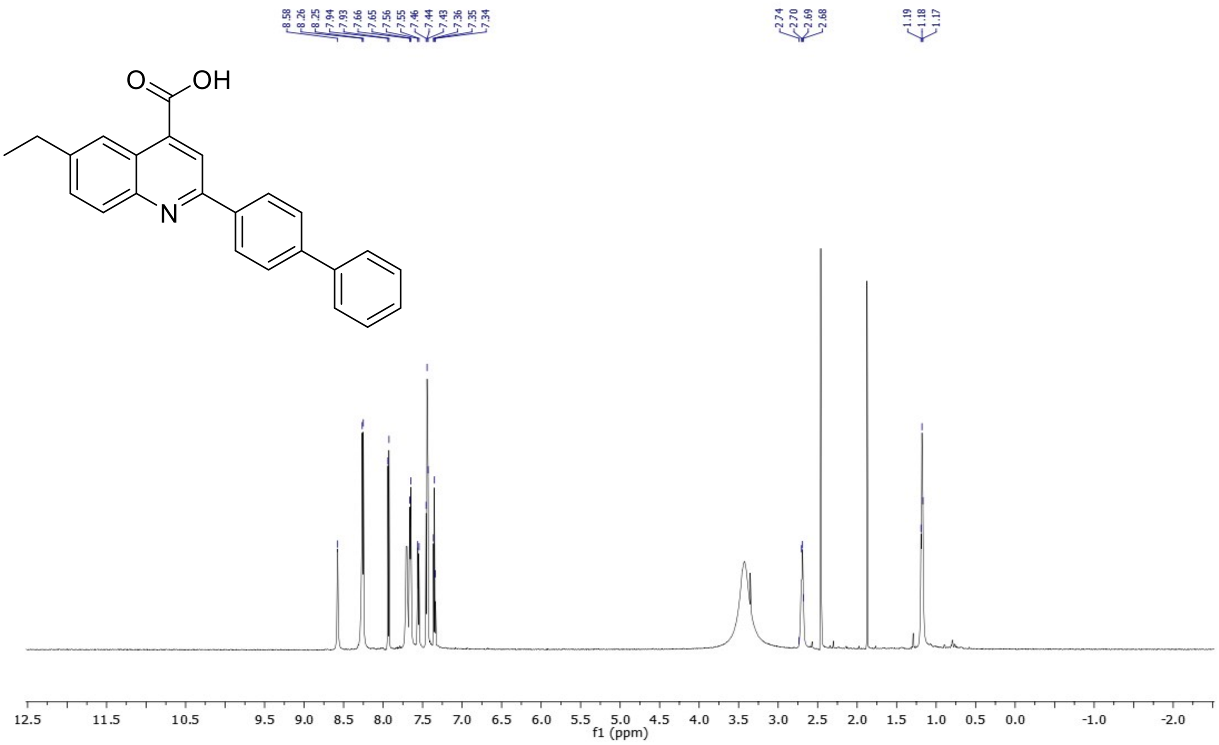

Figure S33. 600 MHz ^{1}H NMR spectrum of **7b** (DMSO-d_6)

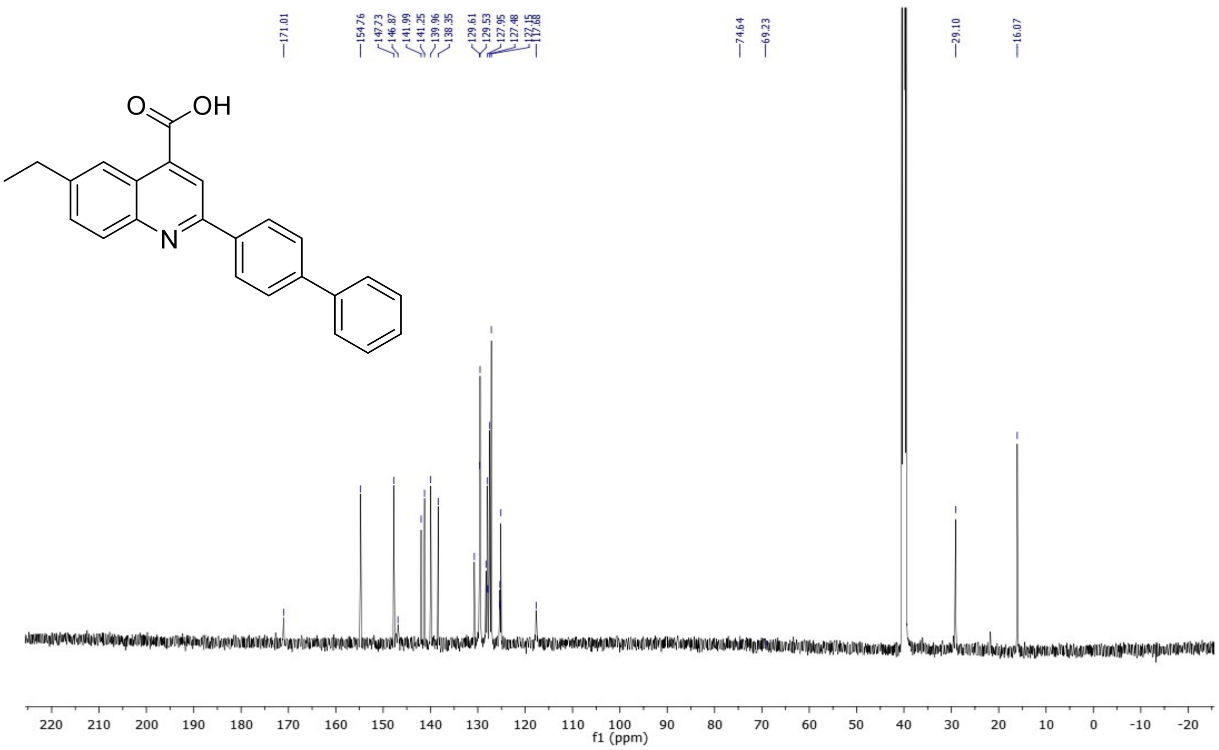

Figure S34. 151 MHz ^{13}C NMR spectrum of **7b** (DMSO-d_6)

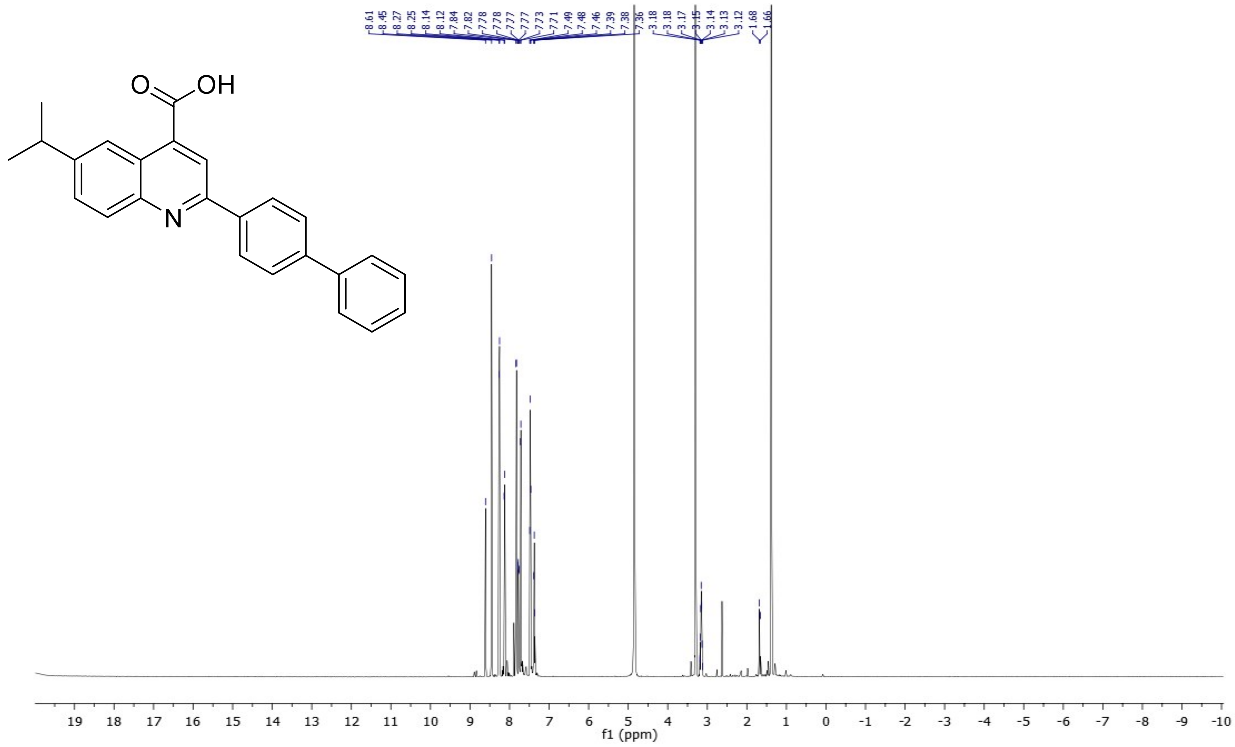

Figure S35. 600 MHz ^{1}H NMR spectrum of **7c** (MeOH-d_4)

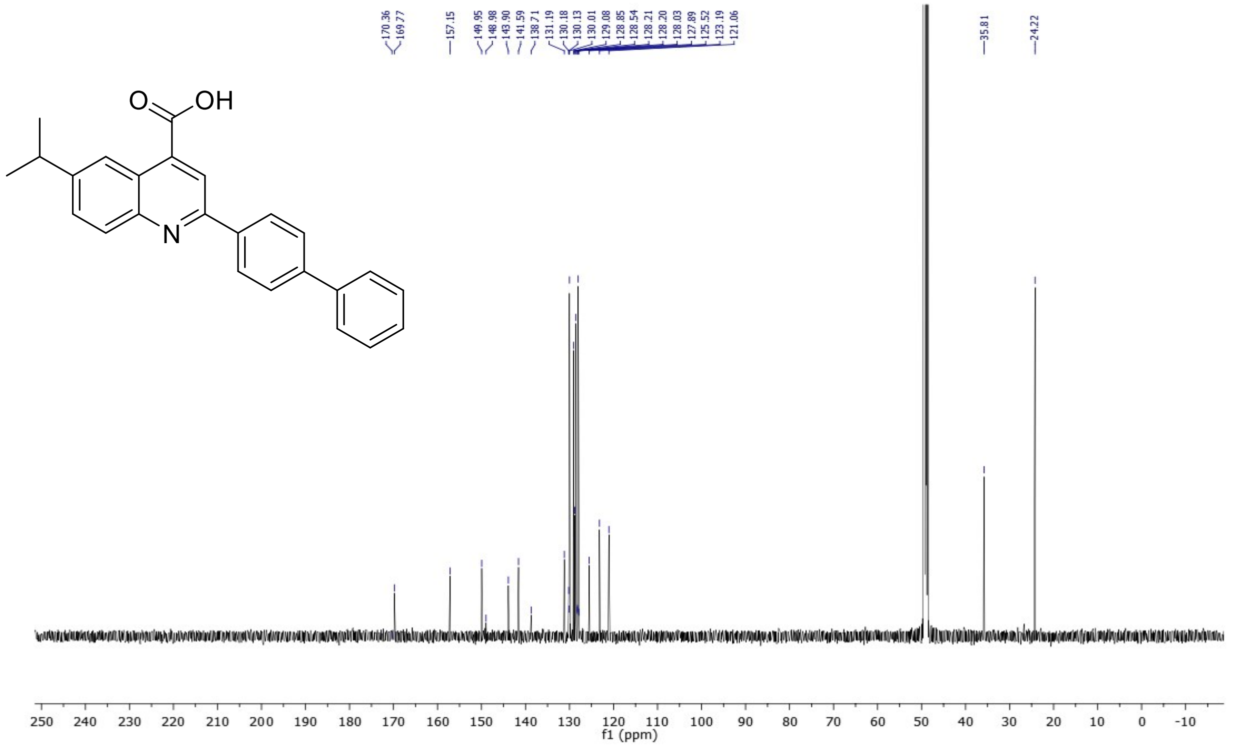

Figure S36. 151 MHz ^{13}C NMR spectrum of **7c** (MeOH-d_4)

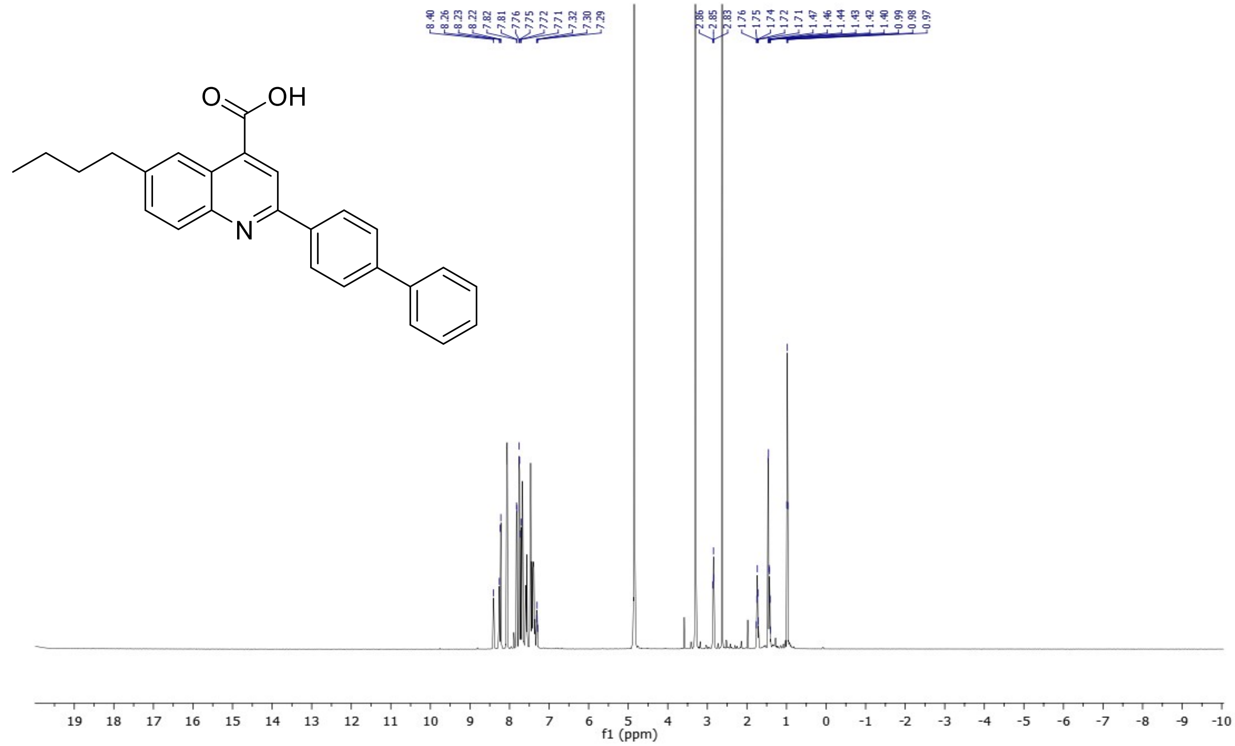

Figure S37. 600 MHz ^{1}H NMR spectrum of **7d** (MeOH-d_4)

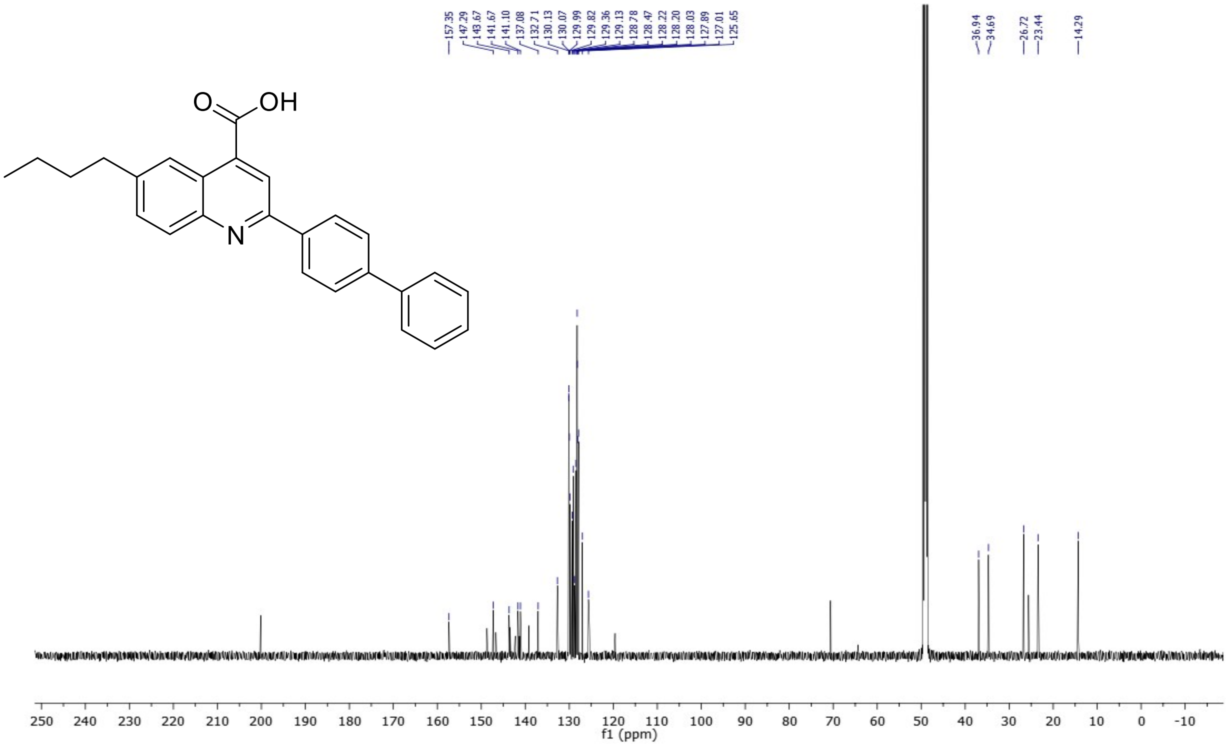

Figure S38. 151 MHz ^{13}C NMR spectrum of **7d** (MeOH-d_4)

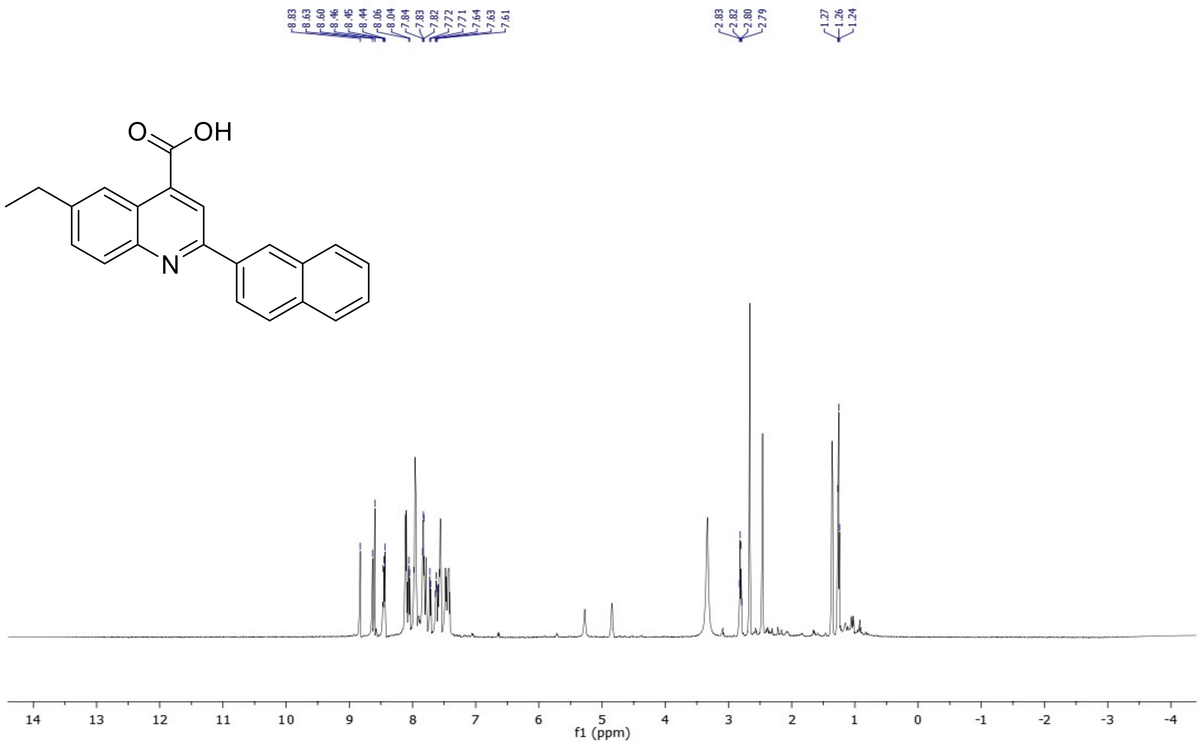

Figure S39. 600 MHz ^{1}H NMR spectrum of **7g** (DMSO-d_6)

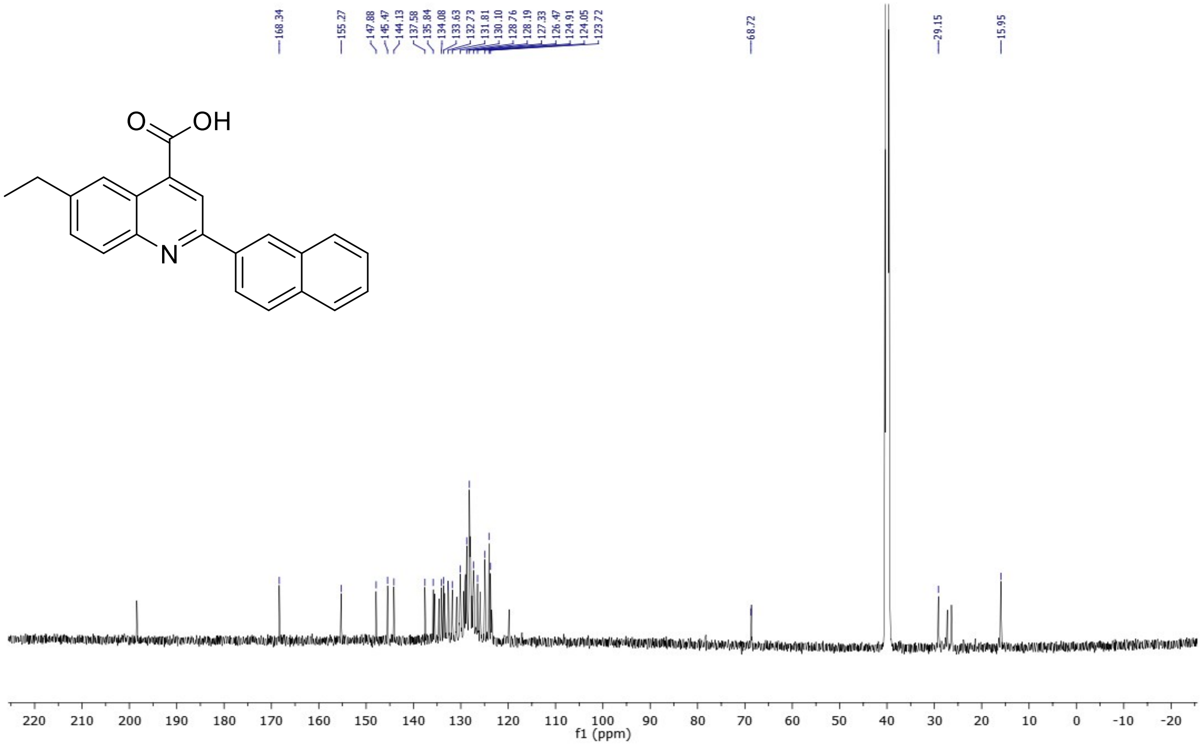

Figure S40. 151 MHz ^{13}C NMR spectrum of **7g** (DMSO-d_6)

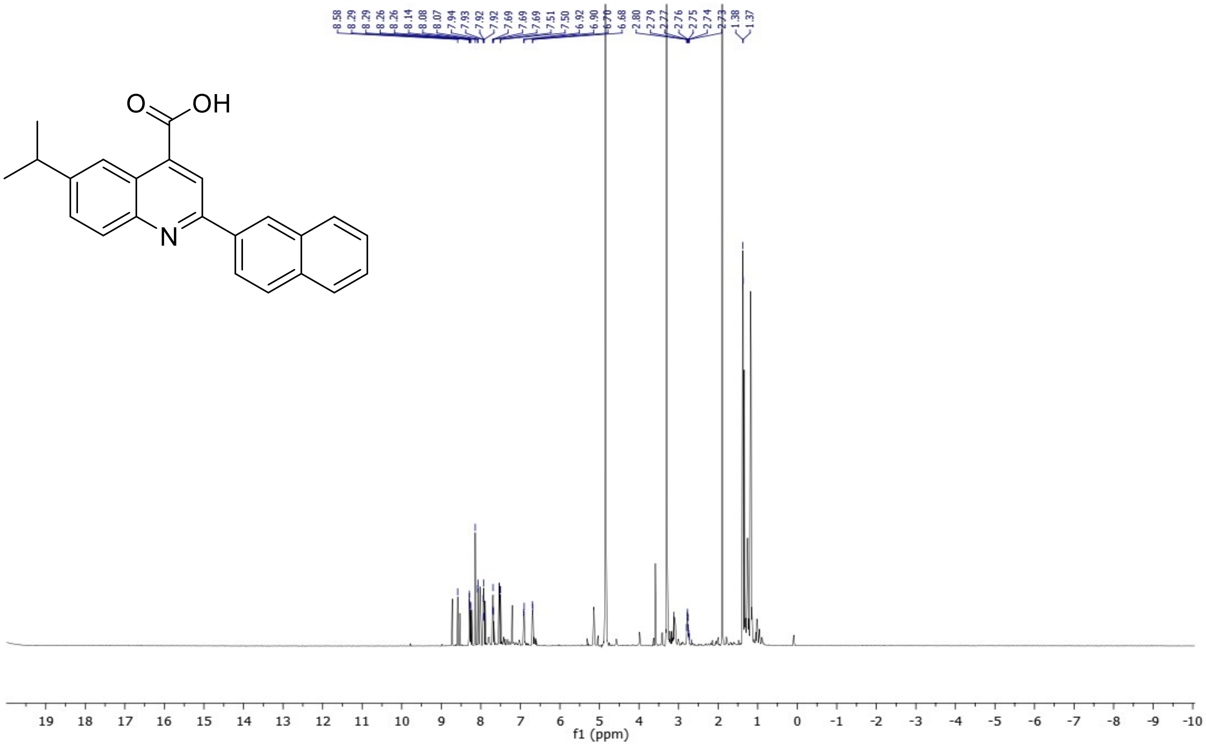

Figure S41. 600 MHz ^{1}H NMR spectrum of **7h** (MeOH-d_4)

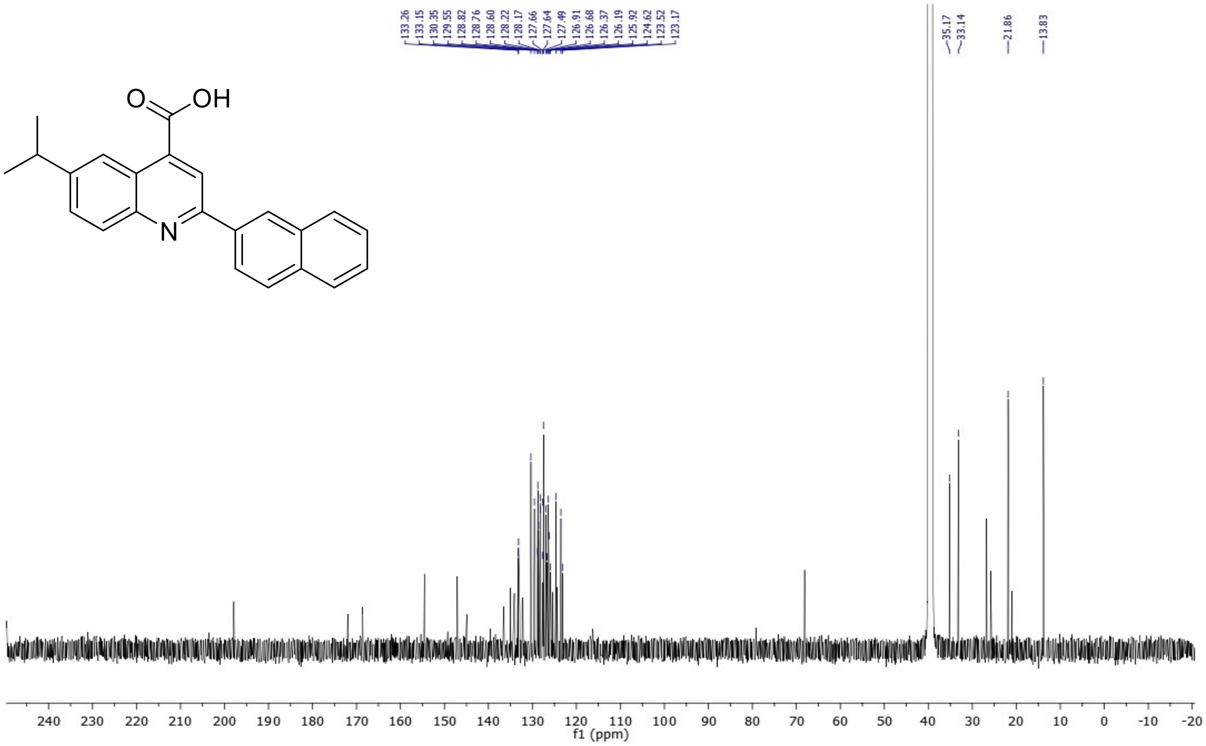

Figure S42. 151 MHz ^{13}C NMR spectrum of **7h** (MeOH-d_4)

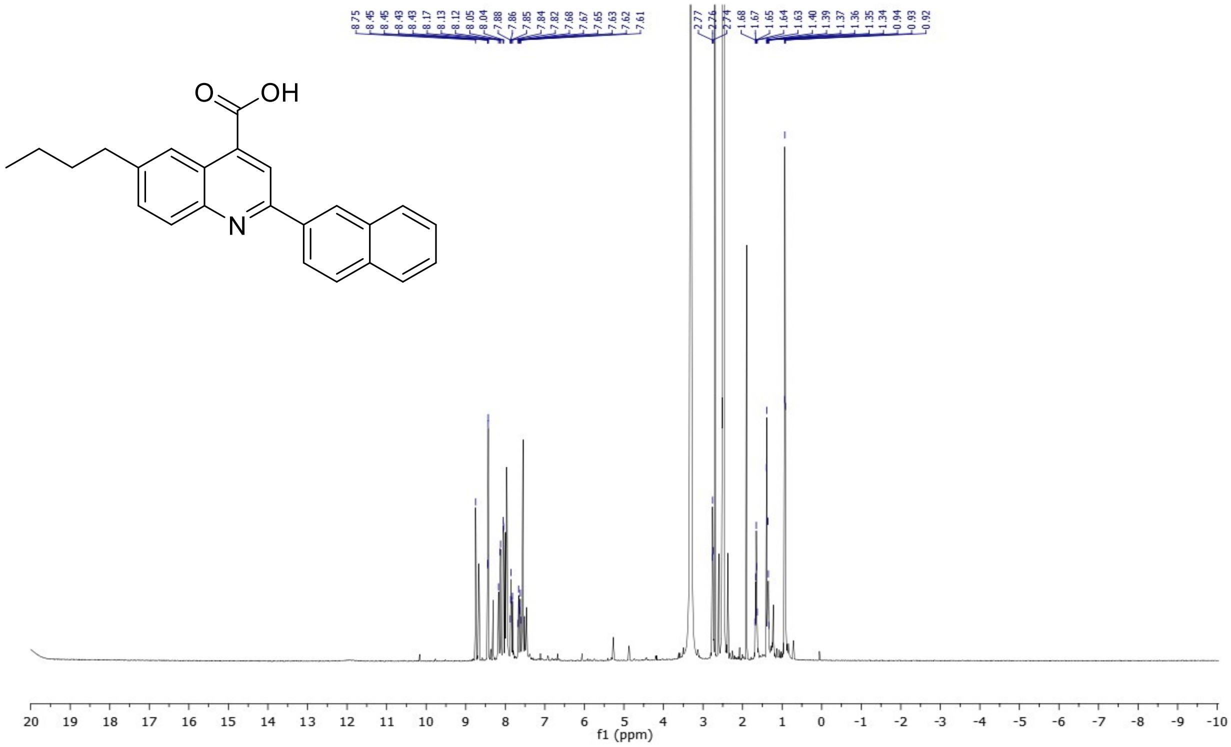

Figure S43. 600 MHz ^{1}H NMR spectrum of **7i** (DMSO-d_6)

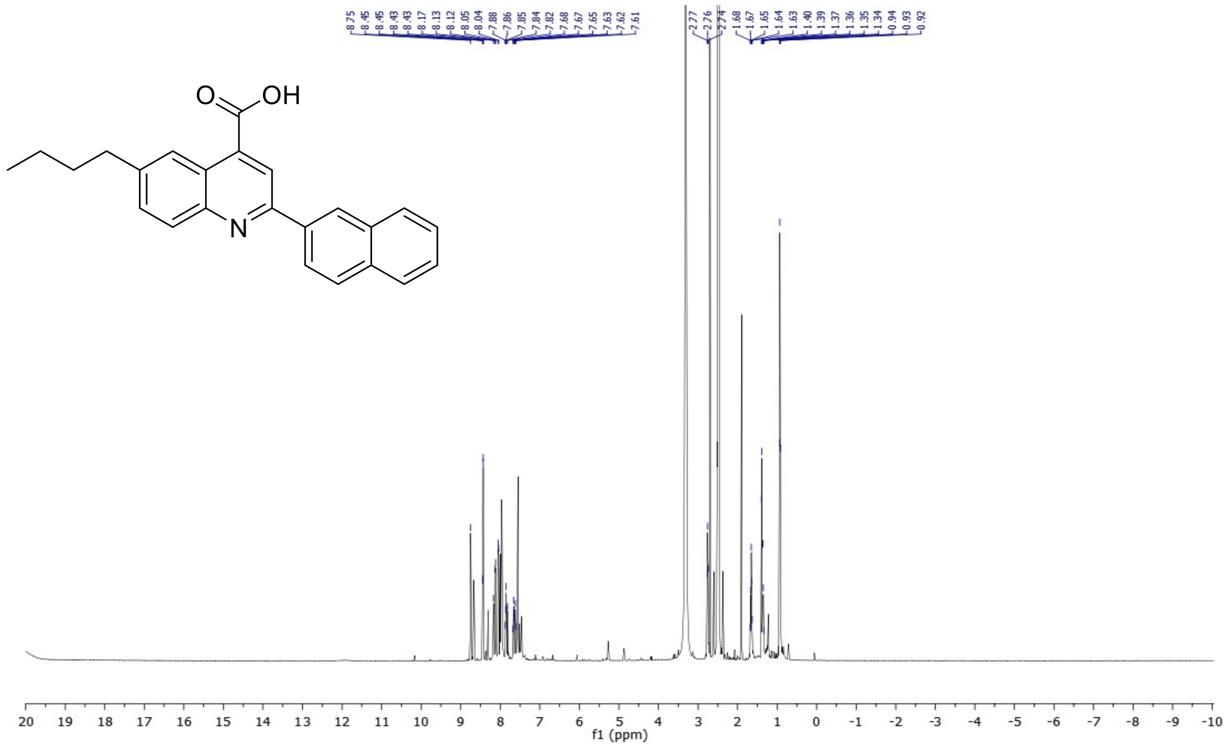

Figure S44. 151 MHz ^{13}C NMR spectrum of **7i** (DMSO-d_6)

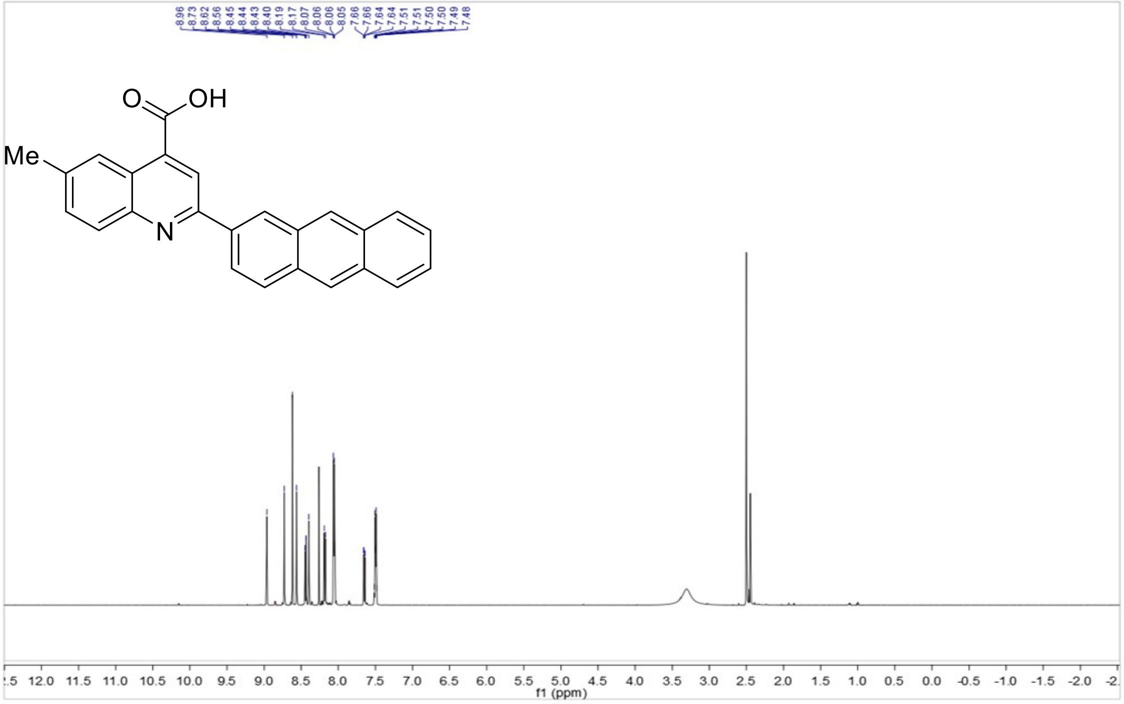

Figure S45. 600 MHz ^{1}H NMR spectrum of **7j** (DMSO-d_6)

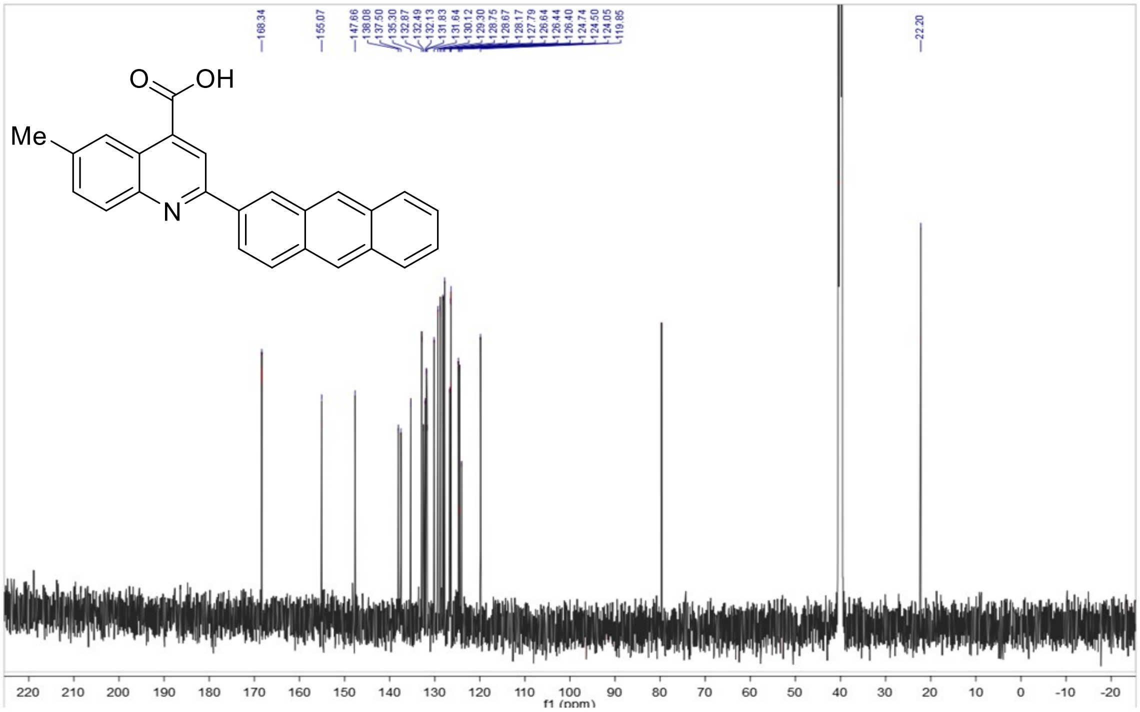

Figure S46. 151 MHz ^{13}C NMR spectrum of **7j** (DMSO-d_6)

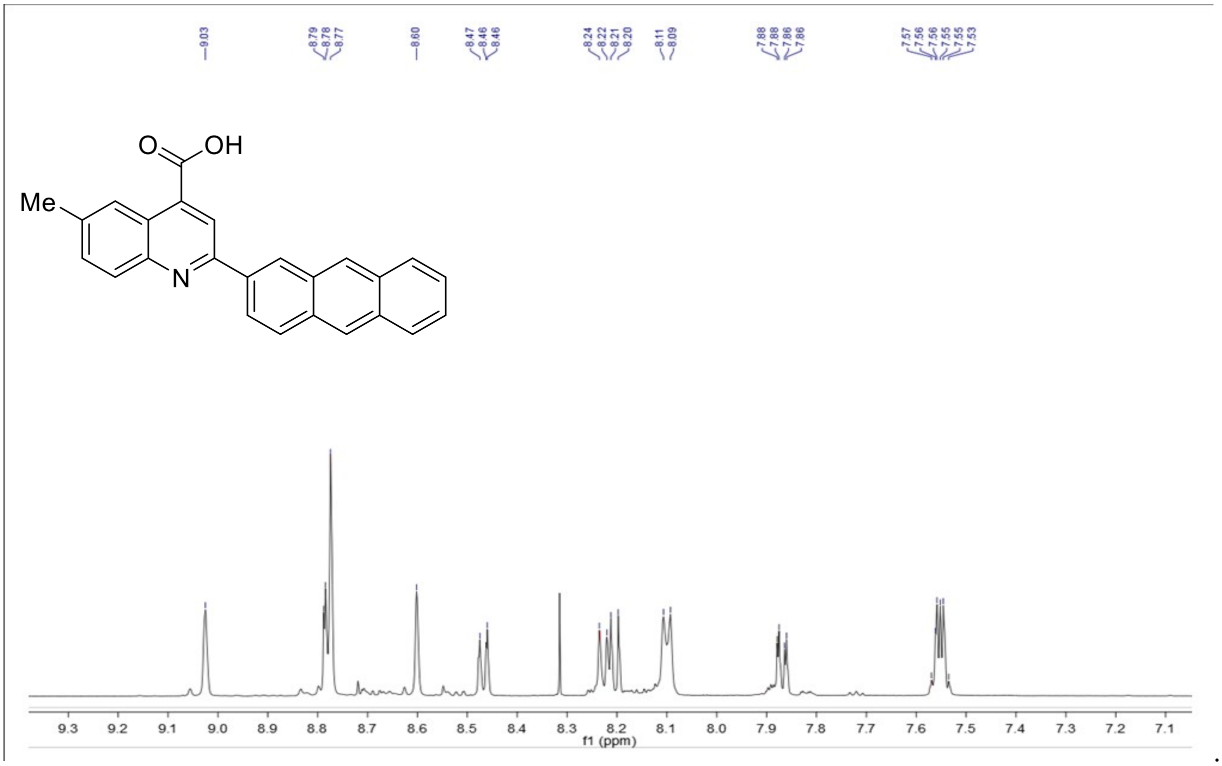

Figure S47. 600 MHz ^{1}H NMR spectrum of **7k** (DMSO-d_6)

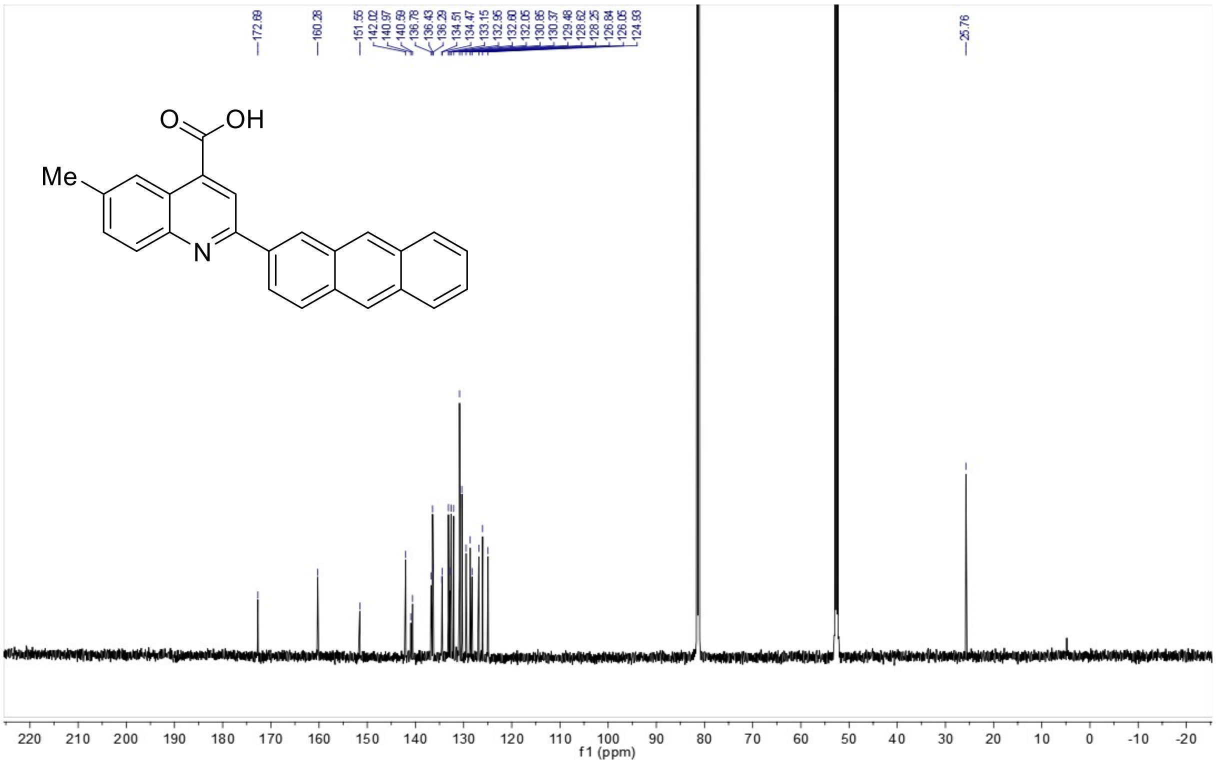

Figure S48. 151 MHz ^{13}C NMR spectrum of **7k** (DMSO-d_6)

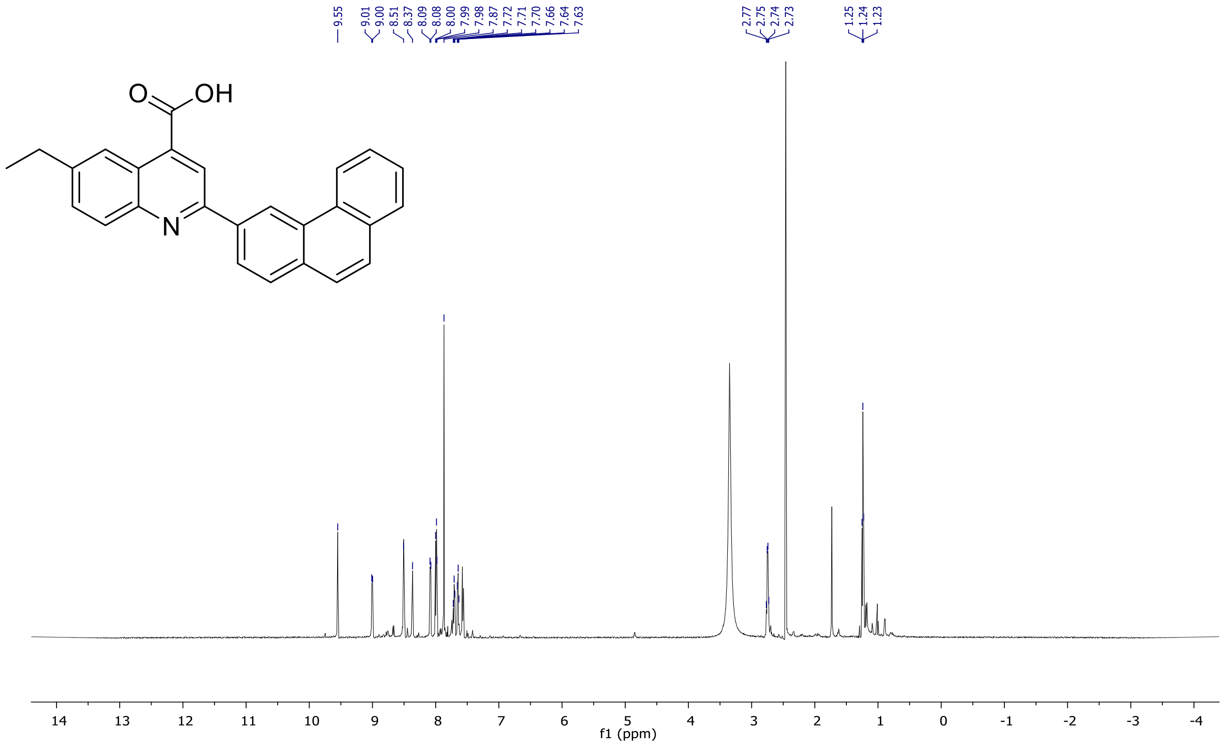

Figure S49. 600 MHz ^{1}H NMR spectrum of **7l** (DMSO-d$_6$)

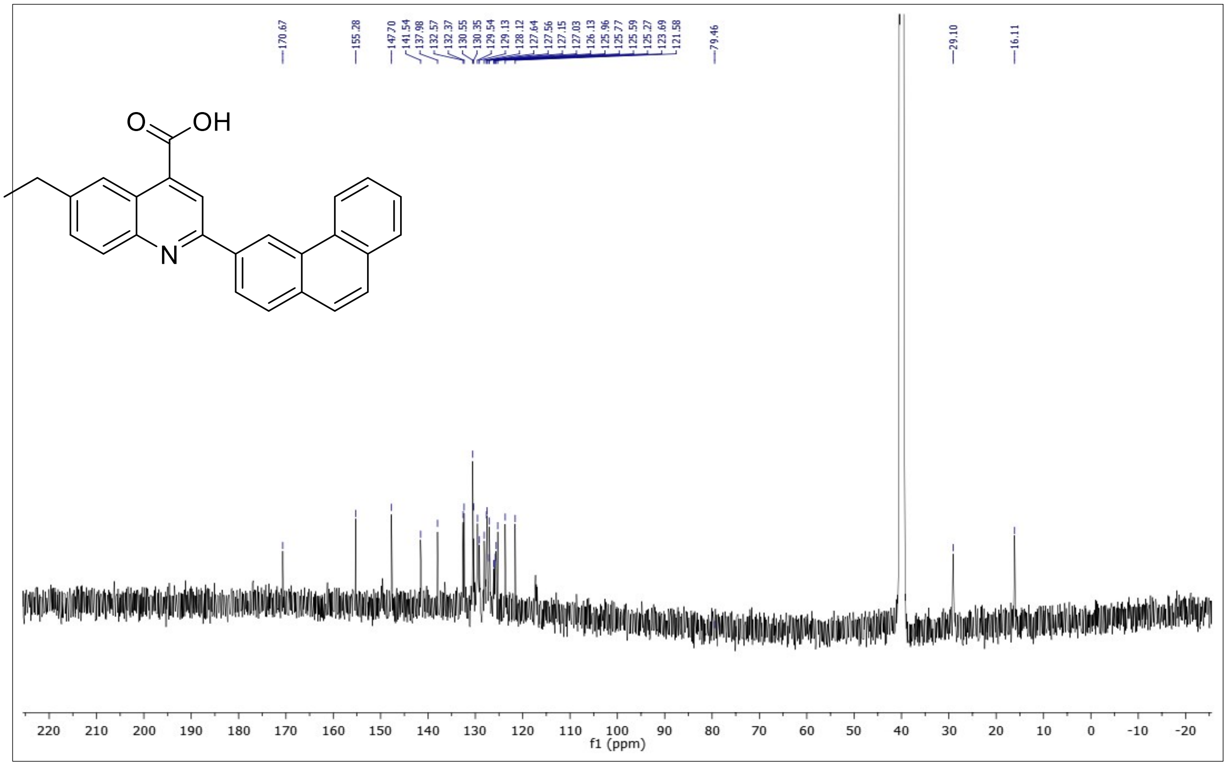

Figure S50. 151 MHz ^{13}C NMR spectrum of **7l** (DMSO-d_6)

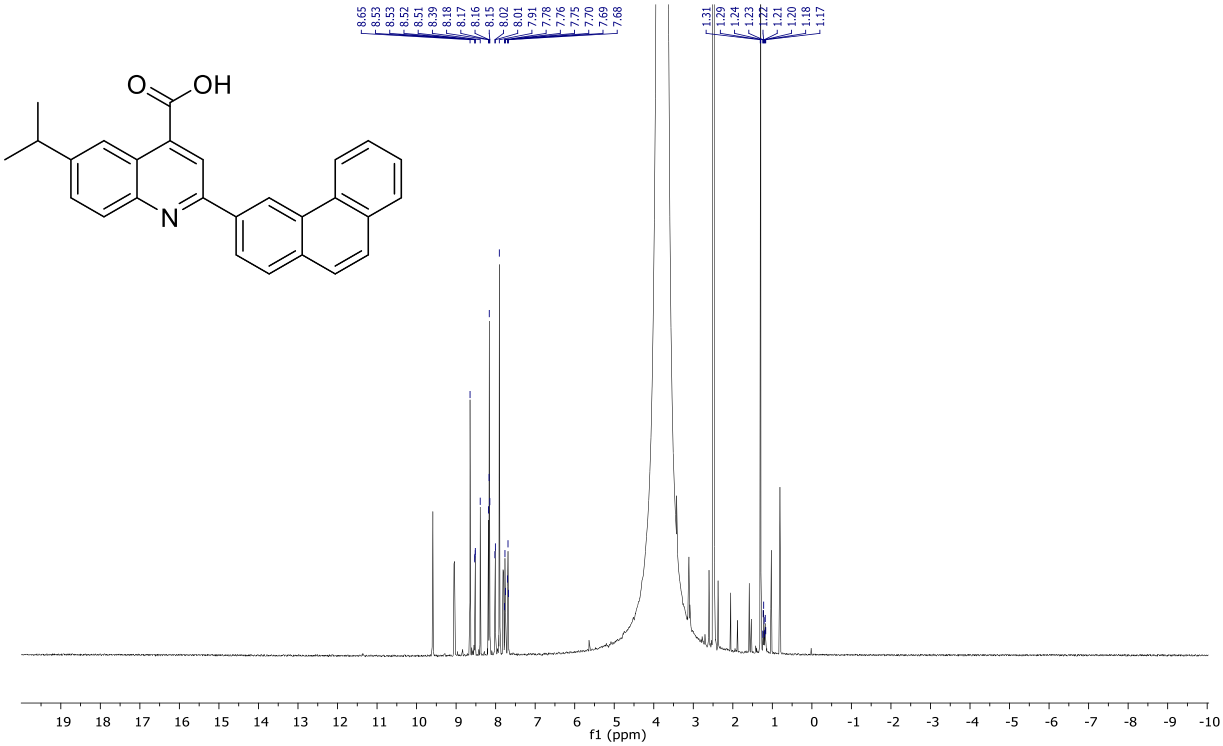

Figure S51. 600 MHz ^{1}H NMR spectrum of **7m** (DMSO-d_6)

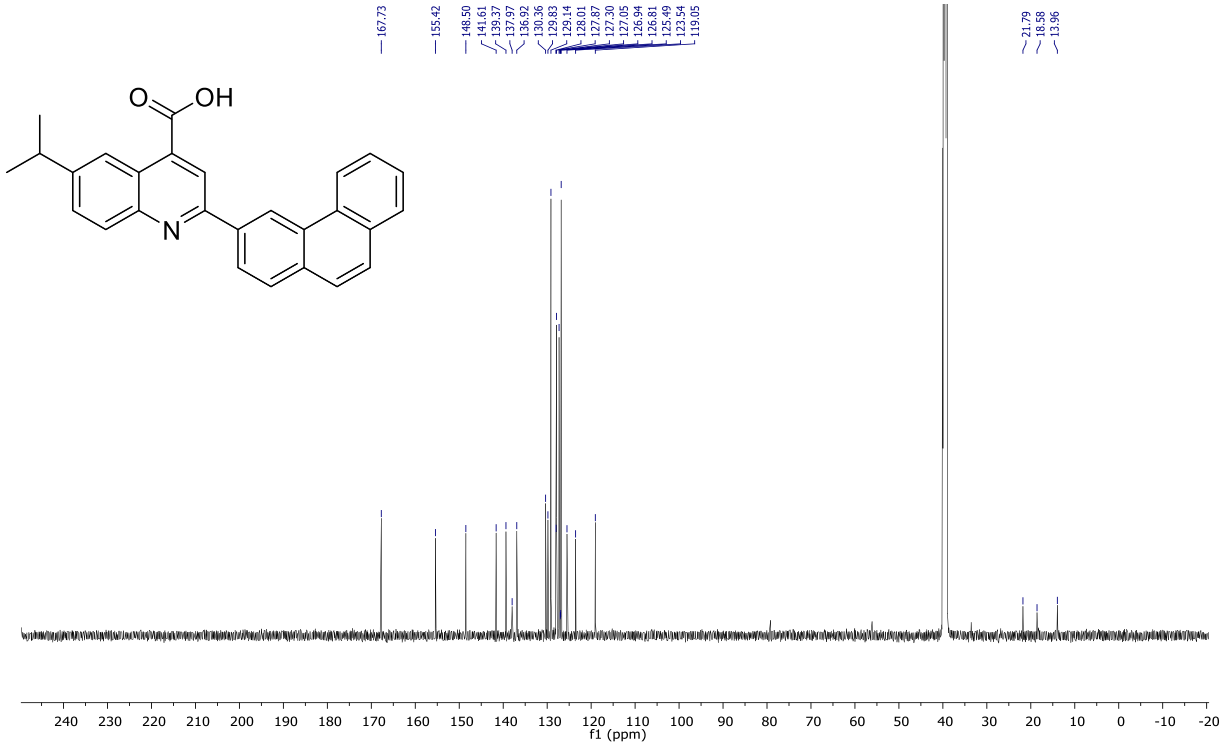

Figure S52. 151 MHz ^{13}C NMR spectrum of **7m** (MeOH-d_4)

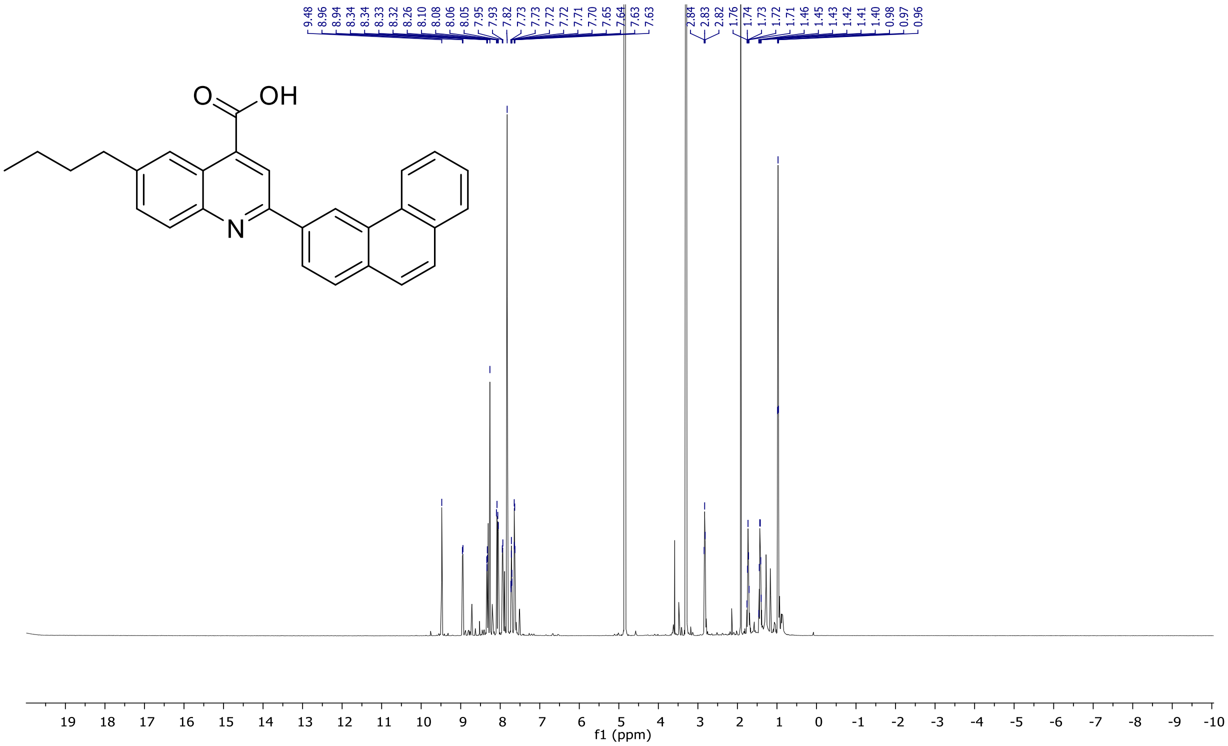

Figure S53. 600 MHz ^{1}H NMR spectrum of **7n** (MeOH-d_4)

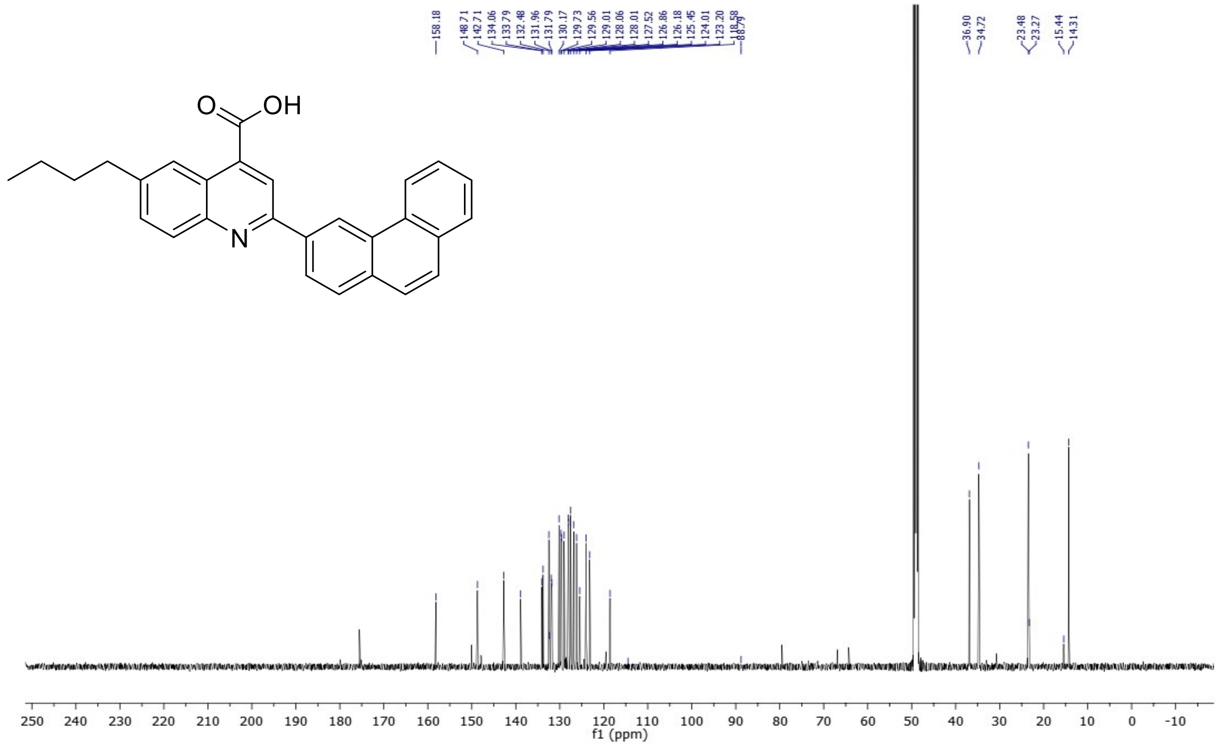

Figure S54. 151 MHz ^{13}C NMR spectrum of **7n** (MeOH-d_4)

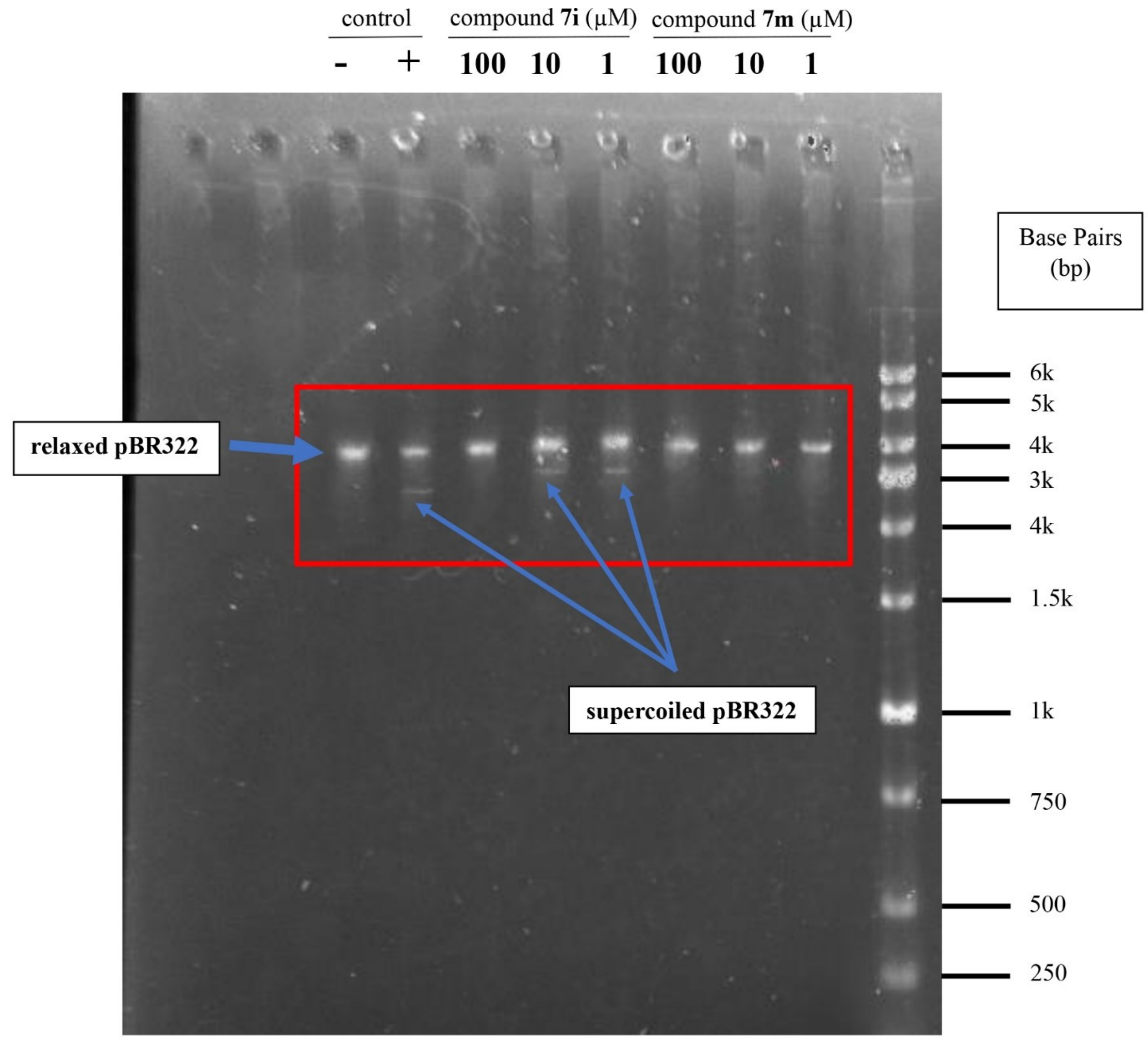

Figure S55. Agarose gel electrophoresis result showing inhibition of QCA **7i** and **7m** against *Mtb* DNA gyrase; (-) negative control without gyrase; (+) positive control with gyrase.
